# Housing and Support Intervention and Mortality Among Homeless Adults With Mental Illnesses: A Secondary Analysis of a Randomized Clinical Trial

**DOI:** 10.1001/jamanetworkopen.2025.24302

**Published:** 2025-07-31

**Authors:** James Lachaud, Rosane Nisenbaum, Cilia Mejia-Lancheros, Eric Latimer, Tim Aubry, Julia Woodhall-Melnik, Jino Distasio, Aynslie Hinds, Daniel Dutton, Julian Somers, Akm Moniruzzaman, Vicky Stergiopoulos, Patricia O’Campo, Stephen W. Hwang

**Affiliations:** 1 MAP Centre for Urban Health Solutions, St Michael’s Hospital, Unity Health Toronto, Toronto, Ontario, Canada; 2 College of Social Work, The Ohio State University, Columbus; 3 Dalla Lana School of Public Health, University of Toronto, Toronto, Ontario, Canada; 4 Applied Health Research Centre, St Michael’s Hospital, Unity Health Toronto, Toronto, Ontario, Canada; 5 Family Child Health Initiative, Institute for Better Health, Trillium, Health Partners, Mississauga, Ontario, Canada; 6 Department of Psychiatry, McGill University, Montreal, Québec, Canada; 7 School of Psychology, University of Ottawa, Ottawa, Ontario, Canada; 8 Department of Social Science, University of New Brunswick, St John, New Brunswick, Canada; 9 Department of Geography, University of Winnipeg, Winnipeg, Manitoba, Canada; 10 Department of Community Health and Epidemiology, Dalhousie University, Halifax, Nova Scotia, Canada; 11 Department of Psychiatry, Faculty of Health Sciences, Simon Fraser University, Burnaby, British Columbia, Canada; 12 Department of Psychiatry, University of Toronto, Toronto, Ontario, Canada; 13 Centre for Addiction and Mental Health, Toronto, Ontario, Canada; 14 Division of General Internal Medicine, Department of Medicine, University of Toronto, Toronto, Ontario, Canada

## Abstract

**Importance:**

Homelessness is an important risk factor for premature death, with individuals experiencing homelessness having substantially higher mortality rates than the general population.

**Objective:**

To assess the association of housing and support interventions with mortality among individuals experiencing homelessness and mental illnesses.

**Design, Setting, and Participants:**

This secondary analysis of a randomized clinical trial included 2255 homeless adults with mental illnesses. The study was conducted in 5 Canadian cities (Vancouver, Winnipeg, Toronto, Montreal, and Moncton). Recruitment took place from October 2009 to July 2011; mortality data were collected until March 30, 2019. Due to the complexity of accessing health administrative data, analyses were conducted and completed between February 2021 and December 2023.

**Exposure:**

Participants were randomized to receive either the Housing First (HF) intervention, which provided immediate permanent, scattered-site housing and support through intensive case management or assertive community treatment to chronically homeless individuals, or treatment as usual (TAU).

**Main Outcomes and Measures:**

Mortality rate ratios were ascertained at each site using health administrative databases. Adjusted hazard ratios were computed using Cox proportional hazard survival models. Random-effects meta-analysis was used to calculate pooled effect sizes across sites.

**Results:**

Of the 2255 total participants, 2108 (93.5%) were successfully linked with health administrative data; among them, 1434 (68.0%) were male, with a mean (SD) age of 40.6 (11.5) years. Mortality rates were not different in the HF compared with TAU groups (pooled log mortality rate ratio, −0.07; 95% CI, −0.36 to 0.22). The pooled adjusted hazard ratio comparing mortality in the HF and TAU groups was 0.83 (95% CI, 0.43-1.22).

**Conclusions and Relevance:**

In this secondary analysis of a randomized clinical trial, the HF intervention was not directly associated with mortality risk. Research is needed to determine whether adjunctive interventions could reduce mortality among homeless individuals with mental illnesses.

**Trial Registration:**

isrctn.org Identifier: ISRCTN42520374

## Key Points

**Question:**

What is the association of a Housing First (HF) intervention with the mortality rates of homeless individuals with mental illnesses in 5 Canadian cities?

**Findings:**

In this secondary analysis of data from 2108 participants in a randomized clinical trial, the difference in mortality rates in the HF and treatment as usual (TAU) groups was not statistically significant. The pooled adjusted hazard ratio comparing mortality in the HF and TAU groups was also not statistically significant.

**Meaning:**

Achieving significant reductions in mortality among homeless adults with mental illnesses will likely require additional interventions beyond the provision of stable housing.

## Teaser

This secondary analysis of a randomized clinical trial assesses the association of a housing and support intervention with mortality among homeless adults with mental illnesses.

## Introduction

Homelessness is a strong risk factor for premature death. Adults who experience homelessness have higher mortality rates than the general population, with mortality rate ratios ranging from 1.5 to 11.5,^1,2,3,4,5,6,7,8,9,10^ depending on age, gender, shelter status, and comorbidity levels.^4,11,12,13^ Homelessness exposes individuals to a number of adverse factors such as infections, extreme weather, substance misuse, and violence.^7,10,14,15^ Studies demonstrate a bimodal pattern of causes of death, with causes such as drug overdose, homicide, and suicide predominating among younger homeless adults, while heart disease and cancer are the leading causes among older homeless adults.^2,5,10,11^ These observations raise important questions about the potential effect of housing provision on all-cause mortality risks for people experiencing homelessness across the lifespan.

Some observational studies have found that moving from homelessness to stable housing is associated with reduced mortality risks^16^ compared with the general homeless population.^15^ However, a randomized clinical trial of permanent supportive housing for 423 chronically homeless persons in Santa Clara County, California, found similarly high mortality rates in both treatment and control groups.^17^ A French randomized clinical trial of a housing and support intervention for 703 homeless adults^18^ implemented in 4 large cities in France—Paris, Marseille, Toulouse, and Lille—showed no difference in the overall 2-year mortality rates in the treatment and control groups. These randomized studies have 2 main limitations. First, because of the relatively small number of participants and relatively short duration of observation, these studies identified a small number of deaths and had inadequate statistical power to reach definitive conclusions. In addition, difficulty in ascertaining the vital status of participants increased the risk of bias, in particular due to potential undercounting of deaths among control participants. For example, in the French study, the number of participants with uncertain vital status was 9.5% in the control group but only 0.6% in the intervention group.^18^

The objective of the present study was to determine the association of housing and support interventions with mortality among homeless adults with mental illnesses. The At Home/Chez Soi study randomized 2255 individuals experiencing homelessness and mental illnesses in 5 cities in Canada to receive either standard-care treatment as usual (TAU) or a Housing First (HF) intervention that provided immediate access to mostly scattered-site housing and supportive services.^19,20^ The vital status of participants was ascertained during follow-up periods ranging from 2 to 9 years after randomization using health administrative databases, and meta-analytic methods were used to pool data across study sites.

## Methods

### The At Home/Chez Soi Randomized Clinical Trial

This study is a secondary analysis from the At Home/Chez Soi study, a randomized clinical trial examining an HF intervention for homeless adults with mental illnesses implemented in 5 cities across Canada, including Vancouver, British Columbia; Winnipeg, Manitoba; Toronto, Ontario; Montreal, Québec; and Moncton, New Brunswick.^19,20^ The trial protocol is found in Supplement 1. Recruitment took place from October 2009 to July 2011, through various community agencies, including hospitals and drop-in centers, and participants were periodically interviewed every 3 or 6 months during the 24-month period following enrollment, until to October 2013.^19^ All participants were asked to provide written informed consent for linkage to health administrative data at the time of recruitment. The study was approved by the Research Ethics Board (REB) at St Michael’s Hospital in Toronto. The data linkage for each site was also approved by the REB of the University of Ottawa, Ottawa, Ontario, and Université de Moncton, Moncton (for the Moncton site); Montreal West-Island Integrated University Health and Social Services Centre REB (for the Montreal site); University of Winnipeg (for the Winnipeg site); and Simon Fraser University, Burnaby, British Colombia (for the Vancouver site).

In contrast with traditional approaches that require adherence to treatment for mental illness prior to moving into permanent housing, HF provides a rent subsidy that facilitates immediate access to permanent housing for chronically homeless individuals in conjunction with supportive services.^21,22^ Three main criteria were used to select study participants: (1) 18 years or older, (2) absolute homelessness or precarious housing, and (3) a diagnosis of a mental illnesses with or without a coexisting substance use disorder as determined by the Mini International Neuropsychiatric Interview at study entry.^20^ Individuals were excluded from the study if they did not have legal residency status in Canada or were relatively homeless (individuals who inhabit spaces that do not meet basic health and safety standards, such as living in overcrowded or hazardous conditions), and those who were already receiving intensive case management or assertive community treatment (ACT) at the time of study recruitment. All study participants were referred by shelters, drop-in centers, hospitals, outreach programs, other homeless services, or self-referral.

**Figure 1.**
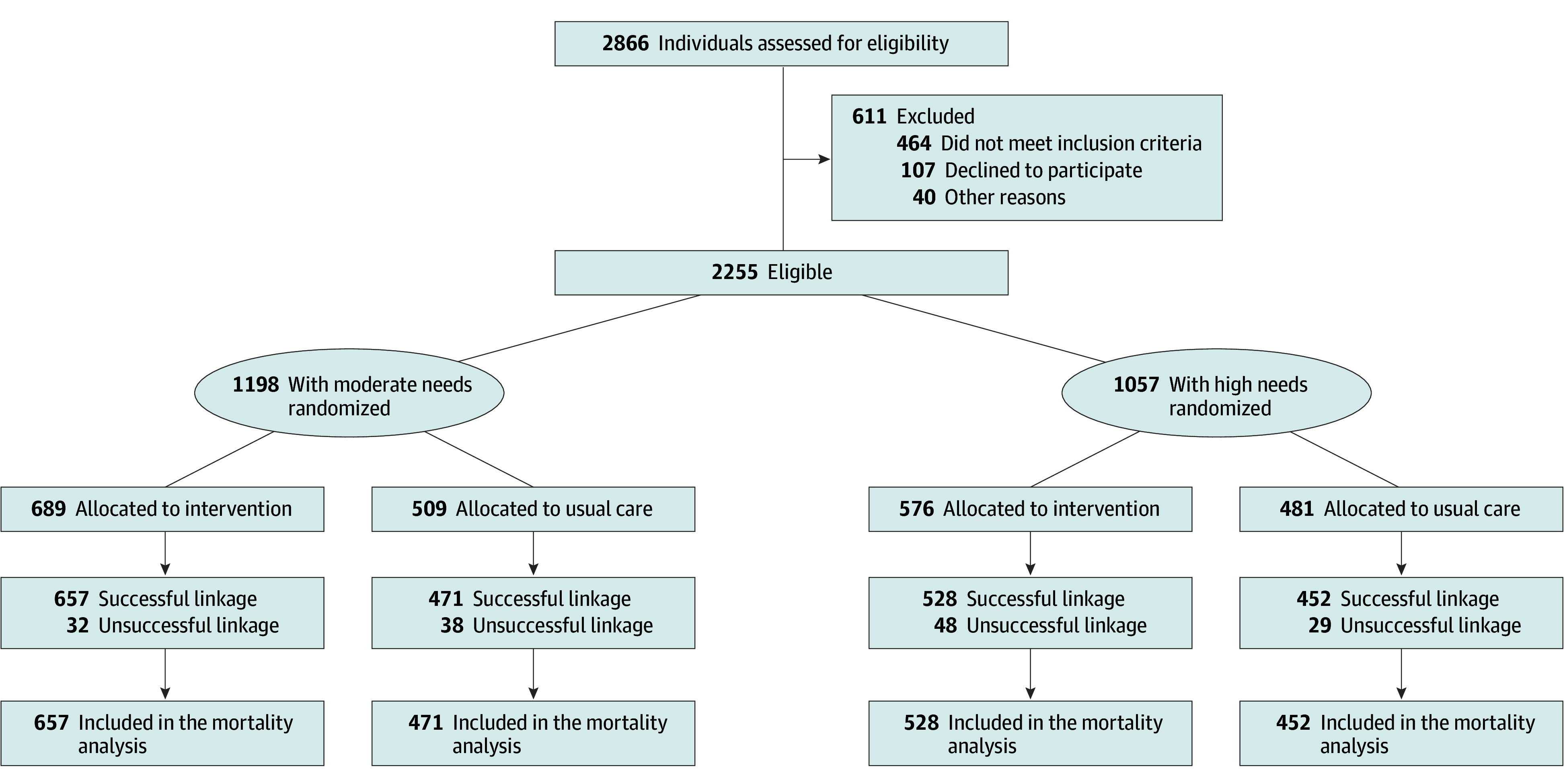
Flowchart of the Study Participants

Study participants were stratified into high- or moderate-needs groups based on diagnosis, acute care service use, comorbid substance use, and justice involvement.^20^ The high-needs group was randomly assigned to HF with ACT or TAU. ACT services provided comprehensive, community-based psychiatric treatment, rehabilitation, and support with 24/7 service coverage to persons with serious mental illnesses.^20^ The moderate-needs group was randomly assigned to HF with intensive case management or TAU. The Moncton site, focused on homelessness in a small city and adjacent rural region, provided ACT to participants with both high and moderate needs because the sample size did not allow for stratification by needs group.^23^ For this reason, when analyses were stratified by needs level, results from the Moncton site were combined with the high-needs groups at the other 4 sites (see Figure 1). The estimation of sample computation and the process recruitment and randomization of participants have been described in a previous publication.^20^

### Vital Statistics Data Linkage

Data on study participants were linked to health administrative databases to ascertain death. Each Canadian province has its own institution that is the repository and analysis center for all health administrative data for residents of that province. The institutions involved in this study were Population Data BC, for participants in Vancouver; the Manitoba Population Research Data Repository, for participants in Winnipeg; ICES (formerly known as the Institute for Clinical Evaluative Sciences), for participants in Toronto; Institut de la Statistique du Québec, for participants in Montreal; and the New Brunswick Institute for Research, Data and Training, for participants in Moncton. Each study site proceeded with the data linkage through a deterministic process using the participant’s provincial health insurance number, or a probabilistic linkage using full name, sex at birth, and date of birth.

### Outcome

The main outcome was ascertained deaths that occurred in the interval between the date of randomization and the last date of administrative data availability, which varied by province: 2 years in Vancouver, 6 years in Winnipeg, 7 years in Toronto and Montreal, and 9 years in Moncton. March 13, 2019, was the latest date of data availability.

### Statistical Analysis

A brief description of the sample by age, self-identified gender, and self-identified race (classified as White and Other race) is provided (Table). Other race included participants of Aboriginal background and those who self-identified as a race other than White. This grouping was used because small cell sizes increased the risk of participant reidentification. The full sociodemographic and clinical profile is found in eTable 1 in Supplement 2. To evaluate the association of the HF intervention with mortality, the analyses were conducted in 2 phases. Due to legal mandates to protect data security and privacy, individual-level data were not allowed to leave each of the 5 institutions holding provincial health administrative data. Therefore, in the first phase of this analysis, mortality analyses were conducted for each site within its respective province. For each site, the number of deaths, exposure time, which refers to the time of observation in the study arm since randomization in person-years, and all-cause mortality rates expressed as the number of deaths per 100 000 person-years of observation were computed for HF and TAU groups overall and stratified by level of needs. Survival analysis was conducted at each study site, using years to death since randomization and Cox proportional hazards survival models to estimate the effect of the HF intervention on mortality. Multivariable models adjusting for age, gender, and self-identified racial group by level of needs were also performed to estimate adjusted hazard ratios (AHRs) with 95% CIs. The proportional hazards assumption was assessed by testing the scaled Schoenfeld residuals.

**Table.** Characteristics of Participants Linked With Health Administrative Data (n = 2108)^a^

Characteristic by study group	City					Total
	Montreal	Moncton	Winnipeg	Toronto	Vancouver	
Age, mean (SD)						
All	44.1 (10.6)	39.8 (11.3)	38.3 (11.2)	39.9 (12.1)	41.3 (11.0)	40.6 (11.5)
HF	44.4 (10.8)	39.7 (11.6)	38.5 (10.9)	38.9 (11.6)	39.8 (11.2)	40.4 (11.4)
TAU	43.6 (10.2)	40.0 (11.0)	38.1 (11.5)	41.0 (12.5)	41.3 (11.0)	40.8 (11.5)
*P* value	.42	.85	.69	.046	.17	.43
Gender, No. (%)						
All						
Male	302 (64.3)	133 (68.9)	318 (65.6)	356 (67.6)	325 (75.1)	1434 (68.0)
Female	168 (35.7)	60 (31.1)	167 (34.4)	171 (32.4)	108 (24.9)	674 (32.0)
HF						
Male	183 (64.2)	68 (68.7)	173 (65.5)	189 (67.5)	193 (75.1)	806 (68.0)
Female	102 (35.8)	31 (31.3)	91 (34.5)	91 (32.5)	64 (24.9)	379 (32.0)
TAU						
Male	119 (64.3)	65 (69.1)	145 (65.6)	167 (67.6)	132 (75.0)	628 (68.0)
Female	66 (35.7)	29 (30.9)	76 (34.4)	80 (32.4)	44 (25.0)	295 (32.0)
*P* value	.98	.95	.99	.98	.98	.99
Self-identified racial group, No. (%)						
All						
White	400 (85.1)	NA	104 (21.4)	194 (36.8)	235 (54.3)	933 (48.7)
Other	70 (14.9)	NA	381 (78.6)	333 (63.2)	198 (45.7)	982 (51.3)
HF						
White	245 (86.0)	NA	47 (17.8)	93 (33.2)	141 (54.9)	526 (48.4)
Other	40 (14.0)	NA	217 (82.2)	187 (66.8)	116 (45.1)	560 (51.6)
TAU						
White	155 (83.8)	NA	57 (25.8)	101 (40.9)	94 (53.4)	407 (49.1)
Other	30 (16.2)	NA	164 (74.2)	146 (59.1)	82 (46.6)	422 (50.9)
*P* value	.52	NA	.03	.07	.77	.77

Abbreviations: HF, Housing First; NA, not applicable; TAU, treatment as usual.

^a^ *P* values based on χ^2^ test comparing HF and TAU groups.

In the second phase of this analysis, we conducted a meta-analysis pooling the number of deaths, exposure time, and sample size from each intervention group and study site to estimate mortality rate ratio. The natural log transformation is often used in meta-analysis when the distribution of estimates in the original scale is skewed or values are strictly positive. Therefore, we considered the natural log of the mortality rate ratio and its SE when pooling results across all sites for all participants, stratifying by needs level. We used a random-effects model with inverse variance weighting that gives a higher weight to study sites with a larger number of participants.^24,25,26^ We also performed a meta-analysis of the AHRs computed from each study site using a random-effects model with inverse variance weighting. For both meta-analyses, the pooled effect size θ with 95% CI was estimated. We assessed heterogeneity in true effect sizes across study sites using the inconsistency index, *I*^2^ value, and the Cochran Q statistic. The *I*^2^ value quantifies the proportion of the variation in point estimates among the differences between the study sites; an *I*^2^ value above 50% is considered substantial or high.^27^ The Q statistic measures the heterogeneity, comparing observed effect size variance with the expected variance, assuming a common true effect across study sites. A low *P* value (typically <.05) associated with the Q statistic suggests significant heterogeneity. We also reported the H^2^ value, which quantifies the excess variation beyond sampling error; H^2^ = 1 indicates all variation is attributable to sampling error. Finally, we created funnel plots to visually assess bias or heterogeneity across study site, examining the distribution of SEs across individual studies.^28^ Meta analyses were performed using the command meta in Stata, version 18.0 (StataCorp LLC). All statistical tests were 2 sided, and differences were deemed statistically significant if *P* < .05. Due to the complexity of accessing health administrative data, analyses were conducted by site between February 2021 and October 2023, and the meta-analysis was completed and reported by December 2023.

## Results

The overall proportion of successfully linked data was 2108 participants (93.5%), including 1185 of 1265 (93.7%) for the HF group and 923 of 990 (93.2%) for the TAU group (eTable 1 in Supplement 2). Of the 2108 successfully linked participants, 674 (32.0%) were female and 1434 (68.0%) were male, with a mean (SD) age of 40.6 (11.5) years as presented in the Table. A total of 933 participants identified as White (48.7%) and 982 as a race other than White (51.3%). A few statistically significant differences were observed between groups: in Toronto, HF participants were younger than TAU participants; in Montreal, HF participants were more likely to report female gender than TAU participants; and in Winnipeg, HF participants were more likely to self-identify as a race other than White than TAU participants. eTable 1 in Supplement 2 includes sociodemographic and clinical characteristics at baseline.

**Figure 2.**
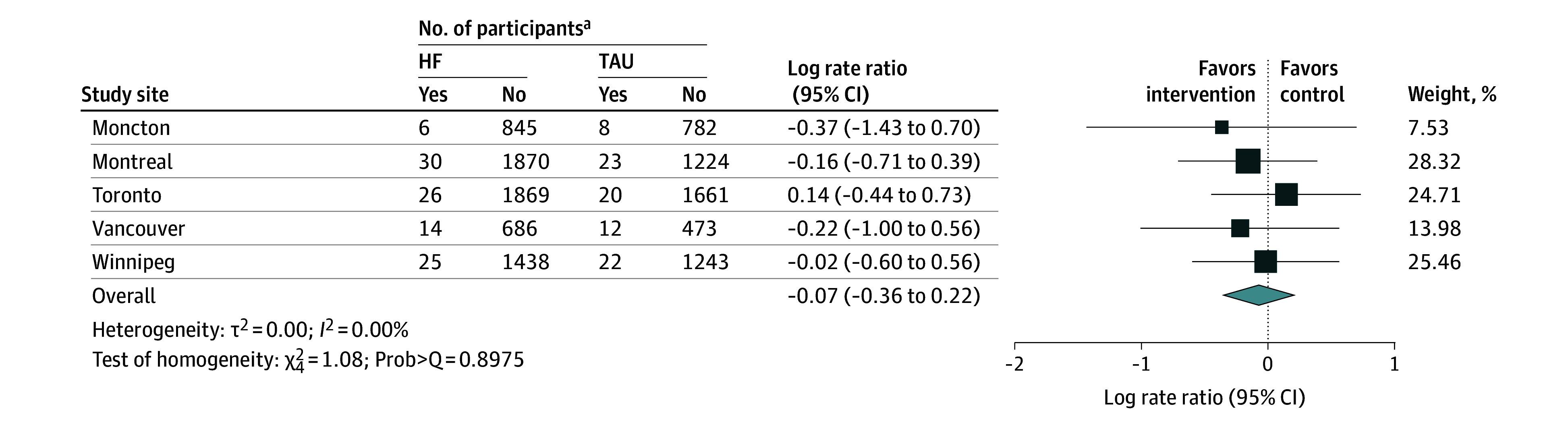
Random-Effects Meta-Analysis of Mortality Rate Ratio Across the 5 Study Sites HF indicates Housing First intervention; TAU, treatment as usual. ^a^Yes indicates patients who died; no, patients who survived.

**Figure 3.**
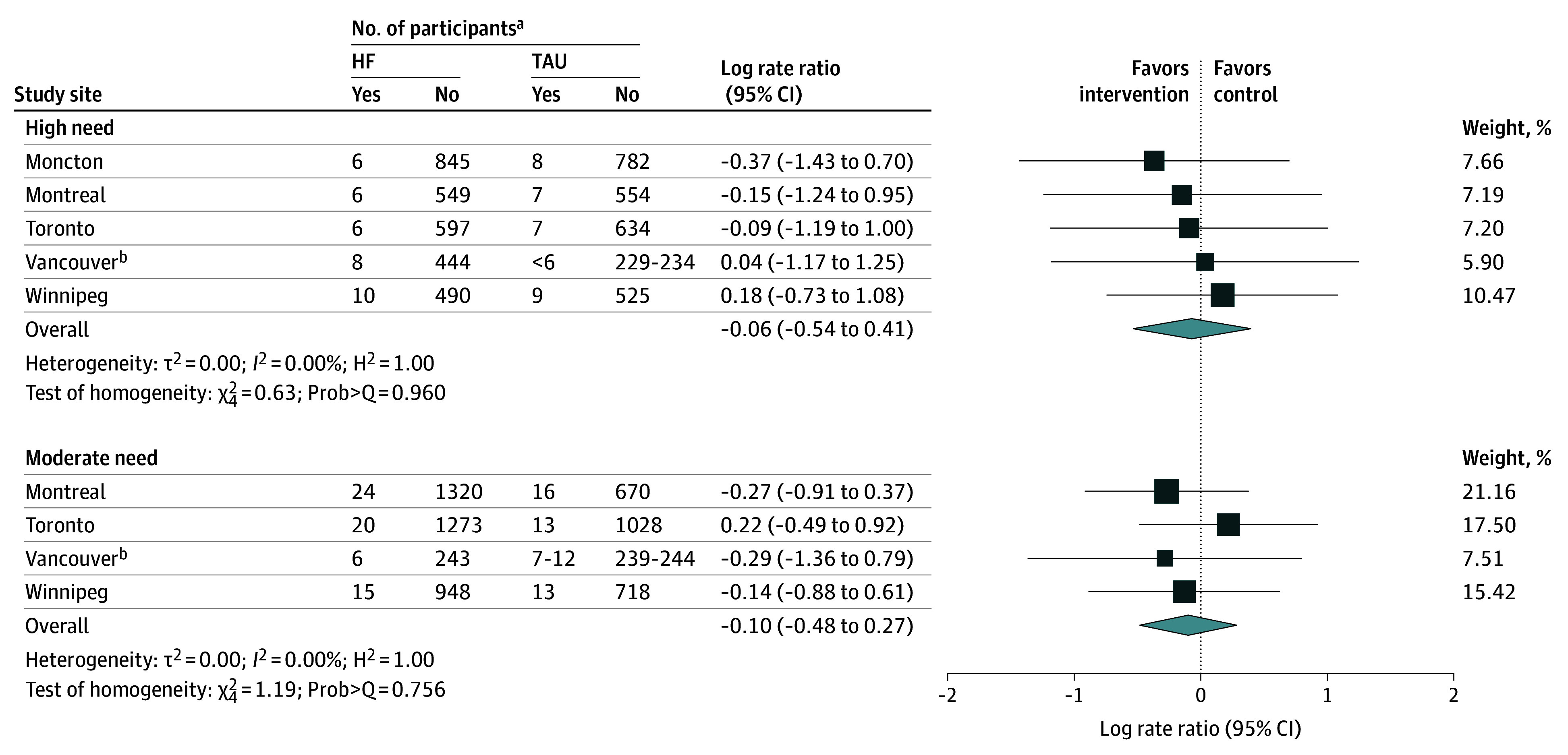
Random-Effects Meta-Analysis of Mortality Rate Ratio Stratified by Need Level and Across the 5 Study Sites HF indicates Housing First intervention; TAU, treatment as usual. ^a^Yes indicates patients who died; no, patients who survived. ^b^To safeguard patient privacy and prevent reidentification, specific values for Vancouver have been grouped at the request of the data providers.

All 5 sites were included in the analysis of the natural log of all-cause mortality rate ratio, comparing the mortality rates between HF and TAU groups. None of the study sites showed a significant difference in mortality rates when comparing HF and TAU groups, as shown in Figure 2. Meta-analysis indicated no heterogeneity across study sites (*I*^2^ = 0; Prob[Q test] = 0.0756) (eFigures 1-3 in Supplement 2). The overall pooled log mortality rate ratio estimate was −0.07 (95% CI, −0.36 to 0.22), indicating no statistically significant difference in mortality rates between HF and TAU groups. Results remained consistent when analyses were stratified by needs level (Figure 3). The log mortality rate ratios were −0.06 (95% CI, −0.54 to 0.41) among high-needs participants and −0.10 (95% CI, −0.48 to 0.27) among moderate-needs participants. The pooled adjusted hazard ratio comparing mortality in the HF and TAU groups was 0.83 (95% CI, 0.43-1.22).

**Figure 4.**
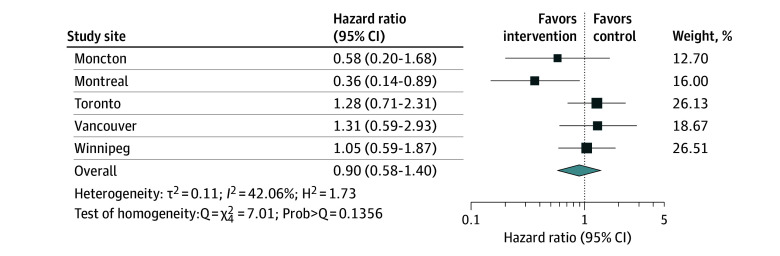
Random-Effects Meta-Analysis of Hazard Ratios of Mortality Across the 5 Study Sites

Meta-analysis pooled estimates of the AHRs are shown in Figure 4. No statistically significant differences in mortality risk were found between the intervention and control groups across all study sites combined, with the pooled hazard ratio being 0.90 (95% CI, 0.58-1.40). Examining AHR at each site, the hazard of death was significantly lower among HF compared with TAU participants in Montreal (0.36; 95% CI, 0.14-0.89) but not at any of the other 4 sites. Heterogeneity across studies was relatively low (*I*^2^ = 42.06%; Prob[Q test] = 0.1356) (Figure 4).

## Discussion

This cohort study leveraged unique data from a 5-site randomized clinical trial of HF linked with health administrative databases to examine the association of housing and supportive care interventions with mortality among homeless adults with mental illnesses. Combining results obtained across the 5 study sites, mortality rates were not significantly different in the group that received the HF intervention compared with the group that received TAU. This finding was consistent for individuals with a moderate or a high level of need for mental health support services. Similarly, the survival analysis revealed that the HF intervention was not associated with mortality risk when compared with TAU.

Our results were consistent with a previous study on mortality in homeless people enrolled in the French Housing First randomized clinical trial,^18^ which showed the overall 2-year mortality rate was relatively higher in the HF group compared to the TAU group (HR, 0.49; 95% CI, 0.24-1.01; *P* = .054), although this difference was not statistically significant. The findings from our study were of particular value due to the larger number of participants (almost 3 times more than the French study) and longer duration of follow-up, yielding greater power.

HF interventions have been shown to be very effective in achieving rapid and stable housing.^29,30^ However, studies have shown less substantial impacts on health status improvement,^31^ use of health care services,^31,32,33^ and other indicators of well-being.^29,34,35^ A 2021 study of the effects of HF at the Toronto site of the At Home/Chez Soi study^32^ found mixed long-term impacts on use of acute care services among participants. For those with moderate needs, the number of all-cause and mental health hospitalizations and ED visits significantly increased during the 7-year postrandomization period compared with TAU; for those with high needs, there was a reduction in the number of days in hospital and the number of ED visits. Another prior study of the Toronto site of the At Home/Chez Soi study^33^ revealed that the intervention had no significant effect on the use of primary care services.

Several factors could explain these findings. First, prior studies^36,37,38^ have shown that experiencing homelessness negatively influences both physical and mental health and is linked to the development of many comorbidities and chronic diseases. These conditions are likely to increase the risk of premature death and may not be amenable to rapid reversibility through the provision of housing. Consequently, individuals with chronic homelessness and mental health disorders have complex health needs^39,40^ that require specialized and tailored health care and follow-up. Second, HF interventions were designed to provide immediate housing, without any preconditions, and research shows that they are indeed highly successful in ending homelessness.^21^ The supports provided by the HF programs in the At Home/Chez Soi study facilitated access to social, mental, and health services,^20^ but may have been insufficient to allow participants to successfully navigate complex and fragmented health care systems.^41^ These difficulties may be particularly challenging for those who have experienced homelessness, serious mental illness, and social exclusion.^29,32^ Finally, although housing provision, as supported by the HF approach, is a key priority to address, it is not enough to guarantee a successful transition from homelessness to complete health, social, and financial recovery.^30,42^ The design of HF interventions does not always address issues related to poverty and neighborhood conditions, factors that are closely linked to mortality risk.^7,43,44^ In some instances, moving into housing might even increase social isolation and loneliness or place the individual in a location with reduced access to health care and other services.^45,46,47^ Recent studies have called for a more coordinated, integrated, and well-funded support system to address the complex needs of individuals experiencing homelessness and mental illnesses.^48^

### Strengths and Limitations

This study has strengths. To our knowledge, it is the first post hoc analysis of a rigorous pragmatic multisite randomized clinical study, with a large sample of 2108 participants, to estimate the association of an HF intervention with mortality among individuals experiencing homelessness and mental illnesses. In addition, it used administrative databases to ascertain vital status of the participants and confirm deaths. However, this study has several limitations. First, there is the inability to assess missing data or linkage rates by site due to data governance restrictions; small cell sizes posed a risk of reidentification, and site-level information could not be disclosed. However, the linkage was successful for 93.5% of the total sample and both HF and TAU groups, indicating minimal overall loss to follow-up. Second, while the study captured whether participants were deceased, it lacked information on the causes of death. This limitation restricted the ability to explore potential mechanisms or specific health risks contributing to mortality within this population. Third, the interventions focused specifically on homeless adults with mental illness, and thus these results may not be generalizable to other subpopulations of people experiencing homelessness. Last, the varying duration of follow-up across cities may have introduced bias when estimating death rates.

## Conclusions

In this secondary analysis of a randomized clinical trial, the HF intervention was not associated with a significant decrease in mortality rates among homeless adults with moderate or high need for mental health services compared with TAU. These findings demonstrate the importance of seeking augmentations or adaptations of the HF model that might better address the complex health needs of this population and reduce the risk of premature death among people experiencing homelessness.

## Notes

Supplement 1.
Trial Protocol

Supplement 2. **eTable 1.** Sociodemographic Characteristics of the Overall AHCS Cohort at Baseline **eTable 2.** Participants Linked With Health Administrative Data, by Study Site **eFigure 1.** Funnel Plot Derived From Random-Effects Meta-Analysis of All-Cause Mortality Rate Ratio **eFigure 2.** Funnel Plot derived from random-effects meta-analysis of All-Cause Mortality Rate Ratio by Level of Need **eFigure 3.** Funnel Plot Derived From Random-Effects Meta-Analysis of Hazard Rations of All-Cause Mortality

Supplement 3.
Data Sharing Statement
