# Supplementary material for: Housing and Support Intervention and Mortality Among Homeless Adults With Mental Illnesses: A Secondary Analysis of a Randomized Clinical Trial
Source: JAMA Netw Open. 2025 Jul 31;8(7):e2524302. doi: 10.1001/jamanetworkopen.2025.24302 (PMC12314730; doi:10.1001/jamanetworkopen.2025.24302)
Supplement: Supplement 2. — eTable 1. Sociodemographic Characteristics of the Overall AHCS Cohort at Baseline eTable 2. Participants Linked With Health Administrative Data, by Study Site eFigure 1. Funnel Plot Derived From Random-Effects Meta-Analysis of All-Cause Mortality Rate Ratio eFigure 2. Funnel Plot derived from random-effects meta-analysis of All-Cause Mortality Rate Ratio by Level of Need eFigure 3. Funnel Plot Derived From Random-Effects Meta-Analysis of Hazard Rations of All-Cause Mortality [file jamanetwopen-e2524302-s002.pdf]

Supplementary Online Content

Lachaud J, Nisenbaum R, Mejia-Lancheros C, et al. Housing and support intervention and mortality among homeless adults with mental illnesses: a secondary analysis of a randomized clinical trial. *JAMA Netw Open.* 2025;8(7):e2524302. doi:10.1001/jamanetworkopen.2025.24302

eTable 1. Sociodemographic Characteristics of the Overall AHCS Cohort at Baseline

eTable 2. Participants Linked With Health Administrative Data, by Study Site

eFigure 1. Funnel Plot Derived From Random-Effects Meta-Analysis of All-Cause Mortality Rate Ratio

eFigure 2. Funnel Plot derived from random-effects meta-analysis of All-Cause Mortality Rate Ratio by Level of Need

eFigure 3. Funnel Plot Derived From Random-Effects Meta-Analysis of Hazard Rations of All-Cause Mortality

This supplementary material has been provided by the authors to give readers additional information about their work.

© 2025 Lachaud J et al. *JAMA Network Open*.

eTable 1. Sociodemographic Characteristics of the Overall AHCS Cohort at Baseline

Variable	Total Sample (N = 2221; 100%)	Housing First (n = 1236; 55.7%)	Treatment as Usual (n = 985; 44.3%)	P-value
Age at enrollment				
Years, mean (SD)	40.89 (11.23)	40.78 (11.15)	41.02 (11.33)	0.617
Sex, n (%)				
Male	1508 (67.9)	834 (67.5)	674 (68.4)	
Female	603 (31.2)	395 (32.0)	298 (30.3)	
Other	20 (0.9)	7 (0.6)	13 (1.3)	0.133
Ethnicities, n (%)				
White	1126 (53.4)	719 (56.2)	407 (49.1)	
non-White	982 (46.6)	560 (43.8)	422 (50.0)	**0.001**
Baseline psychiatric diagnoses, n (%)				
Mood disorder (MDE and manic)	1255 (56.5)	699 (56.6)	556 (56.4)	0.925
PTSD	645 (29.0)	360 (29.1)	285 (28.9)	0.918
Panic disorder	511 (23.0)	270 (21.8)	241 (24.5)	0.133
Psychotic disorder	1095 (49.3)	640 (48.2)	499 (50.7)	0.242
Substance or alcohol use disorder	1498 (67.4)	823 (66.6)	675 (68.5)	0.343
Education, n (%)				
< High school	1241 (56.1)	704 (57.2)	537 (54.7)	
≥ High school diploma	970 (43.7)	526 (42.8)	444 (45.3)	0.240
Lifetime homelessness at baseline, n (%)				

© 2025 Lachaud J et al. *JAMA Network Open*.

<12 months	640 (28.8)	357 (28.9)	283 (28.7)	
12–36 months	576 (25.9)	313 (25.3)	263 (26.7)	
>36 months	1005 (45.2)	566 (45.8)	439 (44.6)	0.746

Percentages are valid percentage of sample and exclude missing data.
MDE, major depressive episode; PTSD, posttraumatic stress disorder.

© 2025 Lachaud J et al. *JAMA Network Open*.

eTable 2. Participants Linked With Health Administrative Data, by Study Site

Cities	# of years post-randomization	HF	TAU	Total	Original Sample
Montreal*	7	285	185	470	470
Moncton	9	99	94	193	201
Winnipeg	6	264	221	485	513
Toronto	7	280	247	527	575
Vancouver	2	257	176	433	497
**Total**		**1185**	**923**	**2108**	
Original sample		1265	990	2255	
% with successful linked data		93.7%	93.2%	93.5%	

* Data from Montreal were rounded using a base of 5 as required by Institut de la statistique du Québec to avoid any potential re-identification risk.

© 2025 Lachaud J et al. *JAMA Network Open*.

eFigure 1. Funnel Plot Derived From Random-Effects Meta-Analysis of All-Cause Mortality Rate Ratio

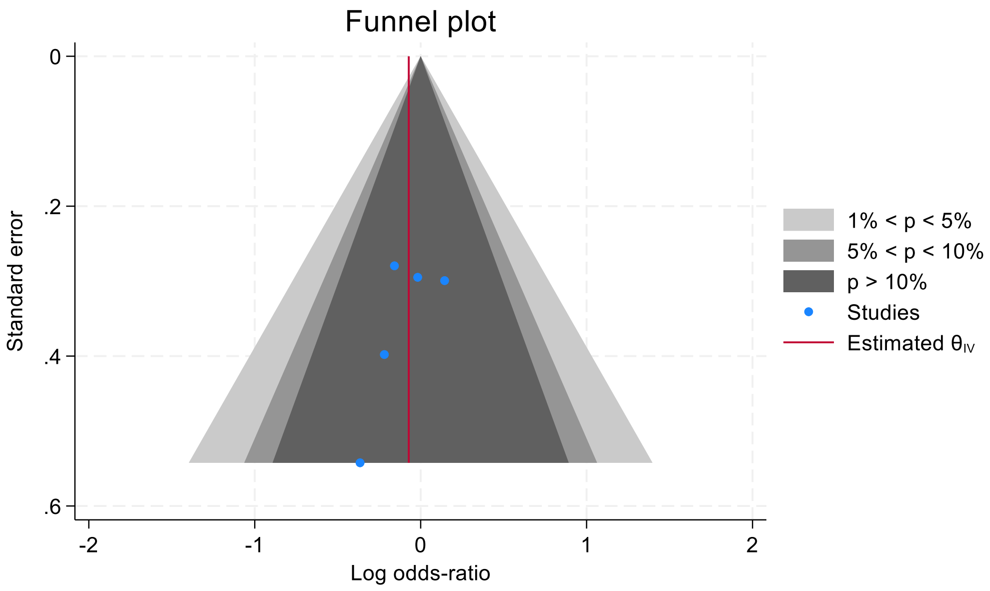

© 2025 Lachaud J et al. *JAMA Network Open*.

eFigure 2. Funnel Plot derived from random-effects meta-analysis of All-Cause Mortality Rate Ratio by Level of Need

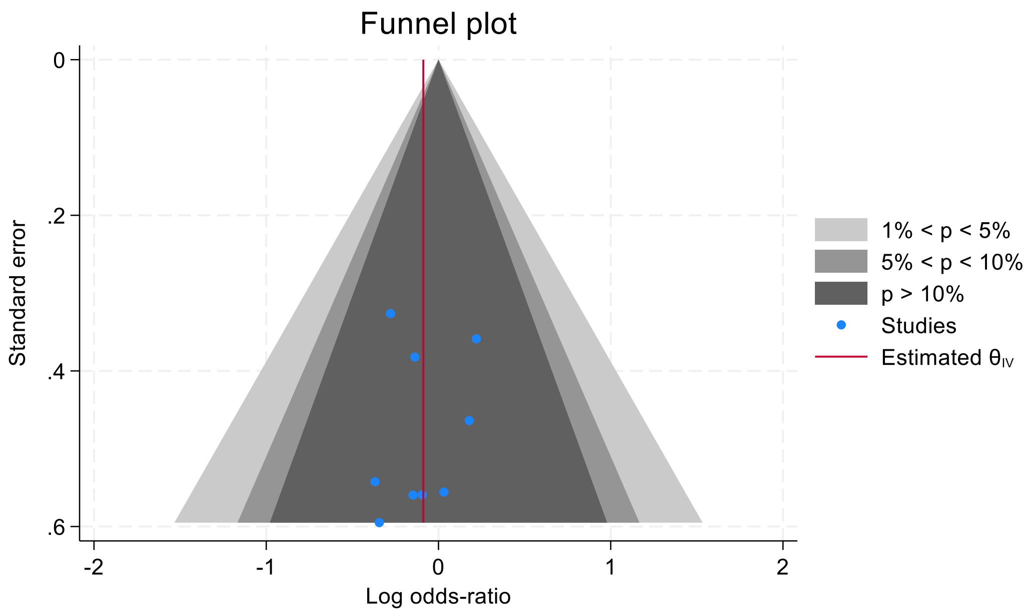

© 2025 Lachaud J et al. *JAMA Network Open*.

eFigure 3. Funnel Plot Derived From Random-Effects Meta-Analysis of Hazard Rations of All-Cause Mortality

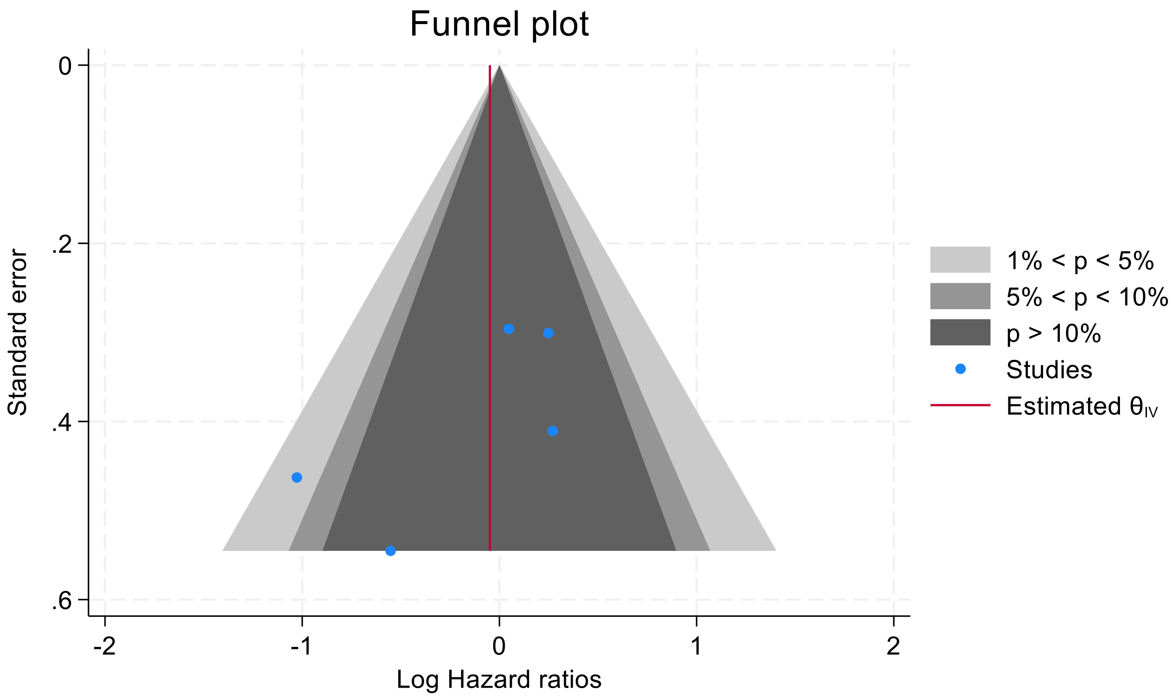

© 2025 Lachaud J et al. *JAMA Network Open*.
