# Supplementary material for: Housing and Support Intervention and Mortality Among Homeless Adults With Mental Illnesses: A Secondary Analysis of a Randomized Clinical Trial
Source: JAMA Netw Open. 2025 Jul 31;8(7):e2524302. doi: 10.1001/jamanetworkopen.2025.24302 (PMC12314730; doi:10.1001/jamanetworkopen.2025.24302)
Supplement: Supplement 3. — Data Sharing Statement [file jamanetwopen-e2524302-s003.pdf]

Data Sharing Statement

Lachaud. Housing and Support Interventions and Mortality Among Homeless Adults With Mental Illnesses. *JAMA Netw Open*. Published July 31, 2025.
doi:10.1001/jamanetworkopen.2025.24302

Data
Data available: No

Additional Information
Explanation for why data not available: The study data have been linked with health administrative data, which are stored within government data centers of Canadian provinces. Unfortunately, these individual health administrative data cannot be shared due to provincial bylaws in Canada.
