# Supplementary material for: Housing and Support Intervention and Mortality Among Homeless Adults With Mental Illnesses: A Secondary Analysis of a Randomized Clinical Trial
Source: JAMA Netw Open. 2025 Jul 31;8(7):e2524302. doi: 10.1001/jamanetworkopen.2025.24302 (PMC12314730; doi:10.1001/jamanetworkopen.2025.24302)
Supplement: Supplement 1. — Trial Protocol [file jamanetwopen-e2524302-s001.pdf]

August 24, 2009

Dr. Julie Spence
Chair, St. Michael's Hospital Research Ethics Board
Research Ethics Board
St. Michael's Hospital
2 Queen Street East, Suite 913
Toronto, ON M5C 3G7

Dear Dr. Spence,

I am pleased to submit for ethical review a study being conducted by scientists at the Centre for Research on Inner City Health. The study is entitled: "At Home" *Research Demonstration Project in Mental Health & Homelessness: Main Study* (REB #09-208). This study is a randomized controlled trial of a complex community intervention for homeless people with serious mental health issues. Dr. Vicky Stergioupoulos, Dr. Patricia O'Campo and I are the Co-Principal Investigators.

This study was submitted for pre-review by a member of the Research Ethics Board on July 30, 2009. We appreciate the feedback provided by this reviewer and would like to point out how his or her concerns have been addressed.

Therapeutic misconception: we have tried to ensure that participants realize that they are primarily part of a research study and are not receiving services or treatment per se by emphasizing this fact in both the main study consent form (Appendix I) and in the information sheet for potential participants (Appendix H).

Undue inducement. The issue of whether our study intervention may present a difficult offer for potential study participants to refuse has been addressed in our protocol (Appendix B – Section 4).

Study completion. A transition plan that ensures adequate housing and supports for each of the intervention group participants is discussed in our protocol (Appendix B – Section 4). The need to transition participants from the study housing if project funding is not extended will be made clear to participants throughout the study and in all study materials (see Appendix G: Referral information for participants and Appendix I: Study Information & Consent Form).

This research project has been developed collaboratively with the City of Toronto's Shelter, Support & Housing Administration Division and with the community-based study service providers. Broad and meaningful community participation has been sought (including input from people with lived experience of homelessness and mental health issues) to shape the content of this protocol, and we have aimed to conduct our work in line with core principles of equitable decision-making, particularly transparency and defensibility.

Please note that this study is related to a recently approved REB Study # 09-161: *Research Demonstration Project in Mental Health & Homelessness: Instrument Pre-Test* and to a recently submitted REB application #09-214: *'At Home' Research Demonstration Project in Mental Health and Homelessness: Planning and Proposal Development Qualitative Evaluation.*

Thank you for the opportunity to submit this application for review by the Research Ethics Board. I look forward to hearing from you about this project.

Sincerely,

Stephen Hwang, MD, MPH

REB FILE NUMBER:	(office use)

Toronto Academic Health Sciences Network (TAHSN)
HUMAN SUBJECTS RESEARCH ETHICS APPLICATION

INSTRUCTIONS

- **All sections** of this application **MUST** be completed before it will be considered for REB review.
- A complete application must be submitted to **each site** where this research will take place.
- A separate detailed protocol must be included with each application.
- All research must be compliant with the Tri-Council Policy Statement which is available at: **http://www.pre.ethics.gc.ca/english/pdf/TCPS%20October%202005_E.pdf, the Ontario Personal Health Information Protection Act (2004 available at http://www.e-laws.gov.on.ca/html/statutes/english/elaws_statutes_04p03_e.htm** and any other relevant regulations or guidelines.
- TAHSN Research Ethics Boards may request and share information related to the review, approval and continuing ethics review of research conducted at other sites.

SECTION I: GENERAL INFORMATION

1. PRINCIPAL INVESTIGATOR NAME

If your institution requires the PI to be a staff member, the on-staff investigator accepts the role and responsibilities of PI at this institution.

Title: Research Scientist	Last Name: Hwang	First Name: Stephen
Credentials (MD, PhD, etc): MD, MPH, FRCPC		

2. FULL STUDY TITLE

"At Home" Research Demonstration Project in Mental Health & Homelessness: Main RCT Study
Sponsor Protocol Number (if applicable):

2A. Is this protocol directly related to a previously approved study at this institution (e.g., extension, rollover, subsequent to a pilot study)? ☒ Yes ☐ No

If **YES**, specify:

Name of Principal Investigator: Stephen Hwang
REB file numbers: 09-161 (Research Demonstration Project in Mental Health & Homelessness: Pre-Test) and 09-214 ('At Home' Research Demonstration Project in Mental Health and Homelessness: Planning and Proposal Development Qualitative Evaluation)

2B. Study Period

Expected start date at this institution: Grant agreement start: April 1, 2009. Participant Enrollment Start: October 2009.
Total study duration at this institution: Grant period is 4 years. Participant study and primary data collection duration is 2 years from enrollment. Secondary data collection (data linkage) will continue for 5 years from enrollment.

3. INVESTIGATORS

3A. Principal Investigator Contact Information and Signature

PRINCIPAL INVESTIGATOR AGREEMENT – I assume full responsibility for the scientific and ethical conduct of the study as described in this application and submitted protocol and agree to conduct this study in compliance with the Tri-Council Policy Statement: Ethical Conduct for Research Involving Human Subjects, Personal Health Information Protection Act (2004) and any other relevant laws, regulations or guidelines. I also agree that if I receive any personally identifiable information (including but not limited to personal health information and biological samples), I will only use or disclose the information as set out in the Protocol, the conditions of the REB, the research participant's consent (unless

consent is waived), and the conditions and restrictions imposed by the relevant information guardian who supplies the information. I certify that all researchers and other personnel involved in this project at this institution are appropriately qualified or will undergo appropriate training to fulfill their role in this project.

<table>
<tr><td>Dept/Div: Centre for Research on Inner City Health</td><td colspan="2">Program:</td><td colspan="2">Institution: St. Michael's Hospital</td></tr>
<tr><td>Telephone: 416-864-5991</td><td colspan="2">Pager:</td><td colspan="2">Fax: 416-864-5485</td></tr>
<tr><td colspan="4">Street Address: 30 Bond Street</td><td>Room/Suite #:</td></tr>
<tr><td>City: Toronto</td><td>Province: Ontario</td><td>Postal Code: M5B 1W8</td><td colspan="2">Email: hwangs@smh.toronto.on.ca</td></tr>
<tr><td colspan="2">Signature of Principal Investigator</td><td colspan="3">Date</td></tr>
</table>

3B. Co-Investigator(s) Contact Information and Signature

CO-INVESTIGATOR AGREEMENT – I agree to participate in this study as described in this application and submitted protocol and agree to conduct this study in compliance with the Tri-Council Policy Statement: Ethical Conduct for Research Involving Human Subjects and any other relevant laws, regulations or guidelines. I also agree that if I receive any personally identifiable information (including but not limited to personal health information and biological samples), I will only use or disclose the information as set out in the Protocol, the conditions of the REB, the research participant's consent (unless consent is waived), and the conditions and restrictions imposed by the relevant information guardian who supplies the information. I will notify the Principle Investigator immediately if there is any deviation from the Protocol or other adverse event.

If one or more co-investigators is a student participating as part of an academic training program, 3C must be completed.

Please see Appendix M for additional co-investigators.

<table>
<tr><td rowspan="2">1</td><td>Title: Research Scientist</td><td>Last Name: Stergiopoulos</td><td>First Name: Vicky</td><td>Institution: St. Michael's Hospital</td></tr>
<tr><td>Dept/Div: Centre for Research on Inner City Health</td><td>Program:</td><td colspan="2">Signature</td></tr>
<tr><td rowspan="2">2</td><td>Title: Director</td><td>Last Name: O'Campo</td><td>First Name: Patricia</td><td>Institution: St. Michael's Hospital</td></tr>
<tr><td>Dept/Div: Centre for Research on Inner City Health</td><td>Program:</td><td colspan="2">Signature</td></tr>
<tr><td rowspan="2">3</td><td>Title: Research Scientist</td><td>Last Name: Bayoumi</td><td>First Name: Ahmed</td><td>Institution: St. Michael's Hospital</td></tr>
<tr><td>Dept/Div: Centre for Research on Inner City Health</td><td>Program:</td><td colspan="2">Signature</td></tr>
<tr><td rowspan="2">4</td><td>Title: Research Scientist</td><td>Last Name: Dunn</td><td>First Name: James</td><td>Institution: St. Michael's Hospital</td></tr>
<tr><td>Dept/Div: Centre for Research on Inner City Health</td><td>Program:</td><td colspan="2">Signature</td></tr>
</table>

5	Title: Research Associate	Last Name: Kirst	First Name: Maritt	Institution: St. Michael's Hospital
	Dept/Div: Centre for Research on Inner City Health	Program:	Signature	

3C. Faculty Supervisor (for student/fellow/resident research studies) ☒ Not Applicable.
NOTE: If this research is part of an academic (University) **training program**, please provide the following information.

☐ Post-Doctoral ☐ PhD ☐ Masters ☐ Undergraduate ☐ Resident/Clinical Fellow

**Name(s) of Student(s)**			
**Name of Supervisor:**			
		Institution:	
Telephone:	Pager:	Fax:	
Street Address:			Room/Suite #:
City:	Province:	Postal Code:	Email:

4. STUDY COORDINATOR/CONTACT PERSON FOR THIS APPLICATION IF NOT THE PRINCIPAL INVESTIGATOR (e.g. study coordinator, research administrative contact, research student, institutional liaison).
☐ Not Applicable

Title: Research Coordinator	Last Name: Mason		First Name: Kate
Dept/Div: Centre for Research on Inner City Health	Program:		Institution: St. Michael's Hospital
Telephone: 416-864-6060 ext 6513	Pager:		Fax:
Street Address: 30 Bond Street			Room/Suite #:
City: Toronto	Province: Ontario	Postal Code: M5B 1W8	Email: masonk@smh.toronto.on.ca

Indicate to whom correspondence should be sent: ☒ Principal Investigator ☒ Study Coordinator/Contact Person

5. DEPARTMENT/DIVISION/PROGRAM HEAD APPROVAL (refer to your institutional guidelines). For institutions that require the PI to be a staff member, approval must come from the Department / Division / Program Head of the same institution as the PI.

Department/Division/Program Head Approval - I am aware of this proposal and support its submission for ethics review. I consider it to be feasible and appropriate. I attest that the Principal Investigator responsible for the conduct of this study is qualified by education, training, and experience to perform his/her role in this study". **This section can not be signed by the Principal Investigator or a Co-Investigator.** An alternative approval signature is required.

Title: Executive Director, Li Ka Shing Knowledge Institute	Last Name: Laupacis	First Name: Andreas
Signature of Dept/Div/Program Head	Date	

6. FUNDING

6A. Source of Funding

Company Name:	
Granting Agency Name:	Mental Health Commission of Canada
Internal Funding:	
Other:	

6B. Status of Funding

☒ Funding obtained	
☐ Funding applied for	Expected date of decision:
☐ No funding required	Explain:

6C. Funding Type/Categories:

List the funder(s):<br>Mental Health Commission of Canada
What category do(es) the funder(s) belong to?<br>(check all that apply)
☐ Industry (e.g. Pharmaceutical Company/ Test or Medical Device Companies / Biotech Company)
☐ Government Funding Agency (e.g. National Institute of Health, Canadian Institutes for Health Research, Medical Research Council)
☒ Government (e.g. National Health Service, Ministry of Health, Department of Defense)
☐ Charitable Foundation (e.g. American Heart Association, The Bill and Melinda Gates Foundation, Wellcome Trust)
☐ Contract Research Organization
☐ Others (describe):

6D. If funding is not awarded, do you plan to proceed with the study? ☐ Yes ☐ No
NOTE: If **YES**, Question 26B. **MUST** be completed.

7. TYPE OF STUDY

Please specify the nature of the study, check **all** that apply.

☒ Chart Review *(please specify):* ☒ Retrospective ☐ Prospective
☒ Clinical Trial (please also complete Question 11)<br>☐Investigational Product or Device study<br><br>(Please specify): ☐ Phase I ☐ Phase 2 ☐ Phase 3 ☐ Phase 4 ☐ unknown ☐ n/a<br>☐ Investigational drug(s)<br>☐ Investigational biologic(s)<br>☐ Investigational natural health product(s)<br>☐ Investigational medical device(s)<br>☐ Approved product for new indication (e.g. new patient population), dosage, or formulation<br><br>Name(s) of Investigational Product(s) or Device(s):<br><br>☒Health Related Intervention(s) (e.g. surgical procedures, behavioural treatments, process-of-case changes, dietary interventions, etc.)<br>(please specify): complex community-based intervention - assignment into one of two groups, (1) intervention (Rent Allowance plus Support Services) or (2) Usual Care.
☒ Qualitative (please check all that apply)

☐ Focus Groups<br>☒ Interviews<br>☐ Observational (e.g naturalistic, field etc.)<br>☐ Questionnaires/Surveys<br>☐ Other. (please specify):
☐ Human Tissue and Biological Specimens (e.g. cadavers, biological fluids, etc.)<br>☐ Banking<br>☐ Biomarker<br>☐ Genetic<br>☐ Other [e.g. pharmacokinetic/pharmacodynamic etc] *(please specify):*
☐ Sub-study Please indicate the REB# of main/related study:
☐ Case Study
☐ Educational
☒ Epidemiological / Database
☐ Quality Assurance / Quality Improvement
☐ Other (please specify):

8. MANAGING CONFLICTS OF INTEREST

Conflicts of Interest do not imply wrong-doing.

It is the responsibility of the PI to determine if **any of the conflicts** listed below apply to **any persons** (listed in Question 3) involved in the research study or any member of their immediate family. Disclose all contracts and any conflicts of interest (actual, apparent, perceived, or potential) relating to this project. Conflict of interest may also arise with regard to the disclosure of personal health information.

NOTE: This disclosure does not replace institutional guidelines and requirements for declaration and management of Conflicts of Interest

☒ Not applicable. There is no Conflict of Interest to disclose.

☐ Function as an advisor, employee, officer, director or consultant for the study sponsor
☐ Have direct or indirect interest in the drug, device or technology employed in this research study (including inventorship, patents or stocks)
☐ Receive an honorarium or other personal benefits from the sponsor (apart from fees for service)
☐ Using services of a family member or a company in which you or a family member has a direct interest.
☐ Receive direct or indirect financial benefit from the disclosure of personal health information
☐ Competing interest
☐ Other (describe)

Describe and detail any Conflicts of Interest.

(Max ¼ page)

How will any Conflicts of Interest be managed?

(Max ¼ page)

9. OTHER INSTITUTIONAL ETHICS REVIEW

9A. Please answer the following and attach ALL RELEVANT CORRESPONDENCE related to ethics and scientific review (e.g. REB review letter, replies, approval letter).

In order to facilitate the REB review process through harmonization and coordination of REB activity, identify if any of the REBs below have reviewed and/or approved the study outlined in this application (check all that apply):		**Ethics Review and Approval Status** (check all that apply and indicate date where applicable):			
		**Application To Be Submitted**	**Applied, Review Pending**	**Reviewed**	**Approved**
☐	Baycrest	☐	☐	☐	☐
☐	Bloorview Kids Rehab	☐	☐	☐	☐
☒	Centre for Addiction and Mental Health	☒	☐	☐	☐
☐	Hospital for Sick Children	☐	☐	☐	☐
☐	Mount Sinai Hospital	☐	☐	☐	☐
☒	St. Michael's Hospital	☐	☐	☐	☐
☐	Sunnybrook Health Sciences Centre	☐	☐	☐	☐
☐	Toronto Rehabilitation Institute	☐	☐	☐	☐
☐	University Health Network	☐	☐	☐	☐
☐	Women's College Hospital	☐	☐	☐	☐
☐	University of Toronto	☐	☐	☐	☐
☐	Other:	☐	☐	☐	☐

9B. Has the research undergone other scientific/scholarly review prior to this REB submission?

☒ Yes (to facilitate further review, please attach all relevant documents) ☐ No

10. RESEARCH SUBJECT TO HEALTH CANADA REGULATION

☒ Not applicable (proceed to Question 11).

10A. Who is the Regulatory Sponsor (i.e. who is listed on the clinical trial application)? ____________________

10B. DOES THIS STUDY INVOLVE SUBMISSION TO HEALTH CANADA UNDER THE FOOD AND DRUG ACT:
☐ Yes ☐ No

If **YES**, is a Health Canada "No Objection Letter" or other regulatory authorization attached? ☐ Yes ☐ No

If **NO**, has an application been made? ☐ Yes ☐ No When? ____________________

10C. Provide the FDA IND Number (Drug Studies) or PMA Number (Device Studies):
FDA IND **#:** ☐ Pending
PMA **#:** ☐ Pending
Not Applicable ☐

NOTE: Final approval will not be granted until the appropriate regulatory approval has been received.

11. CLINICAL TRIAL REGISTRATION (NOTE: This question may be relevant even if you were not required to answer Question 10 above).

The International Committee of Medical Journal Editors (ICJME) has indicated that clinical trials will not be published without the registration of that trial prior to participant enrolment. In June 2007, the ICJME adopted the World Health Organization's definition of clinical trial: "Any research study that prospectively assigns human participants of groups of humans to one or more health-related interventions to evaluate the effects on health outcomes." This definition includes drugs, surgical procedures, devices, behavioural treatments, process-of-case changes and the like. A trial must have at least one prospectively assigned concurrent control or comparison group in order to trigger the requirement for registration." Health related interventions include any intervention used to modify a biomedical or health-related outcome (for example, drugs, surgical procedures, devices, behavioral treatments, dietary interventions, and process-of-care changes). Health outcomes include any biomedical or health-related measures obtained in patients or participants, including pharmacokinetic measures and adverse events.

Given the above definition, indicate whether this trial will be registered (e.g., www.clinicaltrials.gov, www.controlled-trials.com/isrctn/). ☒ Yes ☐ No

If **YES**, provide registration site: **ISRCTN - http://www.controlled-trials.com**

If **NO** please justify:

(Max ¼ page)

SECTION II: STUDY SUMMARY
(The full protocol must still be attached)

Responses to this section are not a substitute for the full protocol.

12. ABSTRACT (suitable for a public access or lay audience).

(Max ¼ page)

This study will examine the effectiveness and cost effectiveness of providing housing and support services for homeless adults in Toronto with serious mental health issues. Specifically, the study will examine the effectiveness of the Housing First model plus either (1) Assertive Community Treatment services or (2) Intensive Case Management services, compared with usual care. The study will also examine the effectiveness of Housing First with Ethno-Specific Intensive Case Management services for homeless adults from ethno-racial groups. The goal is to create evidence for policy makers about what services work best for homeless people with serious mental health issues.

13. RATIONALE AND HYPOTHESIS/RESEARCH QUESTION

13A. What is the rationale for this study?

(Max ¼ page)

Homelessness is a significant social problem that affects thousands of people every night in cities across Canada. A study by the City of Toronto indicates that at least 5,052 individuals are absolutely homeless in Toronto on any given night [1]. The prevalence of mental health problems and addictions among homeless people is significantly higher than in the general population. The literature suggests that between one-fourth and one-third of the homeless have serious mental illnesses such a schizophrenia, major depressive disorder or bipolar affective disorder [2,3]. Further, research suggests that homeless people in Toronto have complex, and often unmet, mental health needs [4,5]. Almost half of Toronto's 2.6 million residents are immigrants including a large proportion of refugee claimants in Canada. A 1999 report by the City of Toronto, identified immigrants and refugees as one of five groups that are particularly vulnerable and in need of targeted strategies [6]. However, the literature concerning the overlap between homelessness, mental

illness and ethnicity, race or culture is scant, particularly in Canadian settings. Overall, there have been only a small number of studies that have examined the effectiveness of housing and support interventions for people with mental illness who are homeless and all of these studies have been conducted in the United States. Research in this area reveals that programs that provide housing and support to people with severe mental illness are effective in reducing homelessness and hospitalizations and in producing other positive outcomes [7]. However, more data are needed that considers both the unique circumstances of ethno-racial groups and homeless people generally within the services and systems available in Canada. *See Appendix A for References.*

13B. What are the study hypotheses or research questions?

(Max ¼ page)

This study has several guiding research questions:
(1) What are the effectiveness, cost/benefit, and cost-effectiveness of a Housing First approach plus Assertive Community Treatment supports in comparison to care as usual for single homeless adults with high need living in urban settings?
(2) What are the effectiveness, cost/benefit, and cost-effectiveness of a Housing First approach plus Intensive Case Management supports in comparison to usual care for homeless adults with moderate needs living in urban settings?
(3) What are the effectiveness, cost/benefit, and cost-effectiveness of a Housing First approach plus Ethno-Specific Intensive Case Management supports in comparison to usual care for homeless adults from ethno-racial groups with moderate needs living in urban settings?
(4) What are the demographic and clinical characteristics that moderate the effectiveness of the interventions?
(5) What are the critical ingredients of each model and modifications needed to effectively implement it in different cities and to effectively serve particular sub-populations?
(6) How do personal stories and lived experience of participants in the intervention and control conditions over time and how can those stories inform the practice, service delivery and policy implications of the study?

See Study Protocol: Appendix B for more details.

13C. What is the significance of the study (i.e. the overall anticipated public and/or scientific benefit)?

(Max ¼ page)

This research is part of a larger multi-site study sponsored by the Mental Health Commission of Canada. Toronto is one of five cities (including Vancouver, Winnipeg, Montréal and Moncton) across Canada where research projects with a common core design to study mental health and homelessness with a focus on Housing First models with supports will take place. A multi-site research ethics board submission will be made to the University of Toronto and the Centre for Addiction and Mental Health pending individual sites' ethics approvals. A National Research Team manages the multi-site study and works collaboratively with investigators at each site to implement and support the research activities. The overall goal of this multi-site study is to provide policy relevant evidence and contribute to developing best practices that will be applicable to future efforts aimed at addressing mental health and homelessness across Canada.

14. STUDY DESIGN

Many of these questions apply to clinical research studies. If any of the items are not applicable to your study, indicate N/A.

14A. Describe the design and methodology (e.g. pre/post design, pilot, study visits, procedures, study intervention).

(Max ½ page)

The study design is a randomized controlled trial in which multiple longitudinal mixed-methods measures will be collected on both the intervention and control groups. The target population for this study is homeless adults with serious mental health issues.

The study involves a total of 560 participants in the Toronto stratified by level of service need and ethno-racial group membership. Participants with high needs (n=200) will be randomized into one of two groups, the intervention (Rent Allowance plus Assertive Community Treatment) or Usual Care. Participants with moderate needs (n=360) will be stratified by ethno-racial group membership and then randomized into one of two intervention options (Rent Allowance plus Intensive Case Management or Rent Allowance plus Ethno-Specific Intensive Case Management) or Usual Care. Study entry will be stratified to ensure that 240 of the 360 moderate needs participants are from ethno-racial groups.

Participants in the Usual Care Group will still be able to access the broad collection of services available in Toronto for homeless people with mental health issues. Participants in this group will be provided with information about available services in the community.

Recruitment to the study may come from several points of entry and will include Toronto's extensive network of organizations serving people who are experiencing homelessness such as shelters, drop-ins, community health centres and emergency departments. An intake research coordinator will assess eligibility and obtain consent. Randomization to group assignment will take place after the baseline interview, which will include classification of high and moderate need. Randomization will occur centrally using the data vendor software. Tests of balance will be conducted to assess the success of the randomization procedures for both the high and moderate needs clients.

Quantitative Survey Component
All study participants (in both the intervention and usual care groups) will be interviewed by phone or in-person every 3 months for a period of 2 years from baseline (for a total of 8 interviews). Participants will be asked questions about their housing situation, physical and mental health status, use of drugs/alcohol and overall quality of life. *See Appendix D: Survey Instrument.*

Physiological measures
Select physiological measures will be collected from all participants at 5 points throughout the study: as part of the baseline interview and at every 6-month interview. The measures being collected are: systolic and diastolic blood pressure, height and weight (to calculate Body Mass Index), and waist and hip circumference.

Data Linkage
Links will be made to administrative data for all participants to obtain information on health care utilization in Ontario, prescription drug use, shelter utilization in Toronto, justice system contacts, disability benefits and official disability diagnoses. The period of observation using these databases will include the 1-year prior to study enrolment and 5 years from enrollment. Individuals who decline permission for linkage to health, social services and justice system databases will still be eligible to participate in the study.

Chart Reviews
The health records of approximately 100 participants will undergo review at baseline and at 24 months. Data will be collected retrospectively for a period of 3 years at enrollment and at 24 months. Data collected from chart review will focus on diagnosis data, preventative health measures (screening and treatment), and select physiological measures where available. *See Appendix F: Chart Abstraction Guide.*

Qualitative Interviews (Personal Narratives)
A subset of 50 participants (20 from the intervention group, 20 from the usual care group and 10 from the ethno-specific treatment group) will be randomly selected from the larger study group and be invited to participate in in-depth qualitative interviews within one month of entering the study and again after 18 months. Participants will be asked to discuss their experiences of homelessness and mental illness and to identify and discuss particularly memorable life events. *See Appendix E: Qualitative Interview Guide.*

Our goal is to retain 75% of participants over 2 years of follow up, using methods that have been proven to be effective in tracking and retaining homeless and marginalized study participants. Participants will be given a laminated card with a toll-free contact number for the study and will be asked to call the contact number once every month for the duration of the study (two years). A $10 cash incentive will paid to the participant for each monthly contact. In addition, at the time of enrolment, participants will be asked permission to allow Ontario Disability Support Program or Ontario Works

to release their current contact information for the purposes of contacting them for additional interviews. To further minimize loss to follow-up, participants will be asked to provide contact information for several friends, relatives and service providers who are most likely to know the participant's future whereabouts. These contact persons will only be contacted when other means of finding study participants have been exhausted. *A script for contact with these individuals is attached in Appendix K.*

14B. Describe the primary outcome measures/goals of the study.

(Max ¼ page)

The primary outcome measures of interest for measuring cost-benefit, effectiveness and cost-effectiveness for this study are:

- Housing Stability (Residential Time-Line Follow-Back Calendar)
- Quality of Life (EQ-5D and SF-12 Health Survey 1.0)
- Mental Health Status (Modified Colorado Symptom Index and Global Assessment of Individual Need - Substance Problem Scale)
- Community Functioning & Integration (Multnomah Community Ability Scale)
- Health care System Use and Costs (Standard health service utilization schedules, administrative data linkage).

For measurement tools please see Appendix D: Survey Instruments.

14C. List all criteria for withdrawal of a participant from the study.

Not Applicable ☒

(Max ¼ page)

We do not envision any premature withdrawal of subjects from the study for safety concerns. Participation in this study is completely voluntary and participants may withdraw from the study at any time.

Participants in the intervention group will not lose their housing or support services should they decide to withdraw from the research activities at any point. Given the pragmatic nature and the population of this study, we feel that it is ethically perilous to consider withdrawing housing and supports if a participant refuses to continue with the research activities of the project, especially since a return to a non-research situation would mostly likely be a return to homelessness. Moreover, if participants are told that their housing and supports will be removed if the do not comply with treatment then this may be viewed as undue influence on the part of the research team.

14D. Is a placebo used in this study? ☐ Yes ☒ No

If **YES**, explain how this is this justified (e.g. no alternative standard treatment available). Include any provisions in place to minimize risks to participants assigned to placebo (e.g., increased monitoring, rescue medication).

(Max ¼ page)

14E. Does this study involve deception or intentional lack of disclosure? ☐ Yes ☒ No

If **YES**, justify and indicate how participants will be debriefed.

(Max ¼ page)

14F. Will the participant be withdrawn from or denied usual therapy for any condition in order to participate in the study? ☐ Yes ☒ No

(This would include medications that are prohibited or restricted in order to be eligible for the study or that may be prohibited or restricted during the course of the study.)

If **YES**, explain.

(Max ¼ page)
Please note: Participants will still be able to access the broad range of services available in Toronto for homeless people with mental health issues. Participants will be provided with information about available services in the community.

14G. Will the participant be subject to other restrictions during the study? ☐ Yes ☒ No

If **YES**, explain.

(Max ¼ page)

15. PARTICIPANT/CONTROLS

15A. List the inclusion and exclusion criteria.

(Max ¼ page)

The main criteria for inclusion in the project are as follows: (1) legal adult status (age 18); (2) meets definition of 'absolute homelessness' or 'precariously housed'; (3) presence of any serious mental health disorder; (4) not currently receiving Assertive Community Treatment or Intensive Case Management services.

Please see Study Protocol (Appendix B) and Screening Tool (Appendix C) for definitions and detailed eligibility cut-off scores

15B. Are there any age, ethnicity, language, gender or race-related inclusion or exclusion criteria?
☒ Yes ☐ No

If **YES**, justify.

(Max ¼ page)

The study includes only individuals of legal adult status (18 years or older) because there is no evidence that the intervention we are evaluating works with youth.

In order to obtain data that are generalizable, women will be targeted to make up 25% of the total sample. If a large proportion of men are enrolled early in the study, men may be excluded from enrollment later on in the study in order to allow us to reach our target of female participants.

Study entry will also be stratified to ensure that 320 of the 560 participants are from ethno-racial groups, 80 among the high needs group and 240 among the moderate needs group. In order to achieve acceptable statistical power levels and be able to definitively evaluate the ethno-specific ICM model and conduct analysis by gender we need to ensure significant numbers of women and individuals from ethno-specific groups are enrolled in the study.

Membership to ethno-racial group will be determined by visible minority status, regardless of immigration status or place of birth, based on responses to demographic questions obtained at baseline.

15C. Indicate the rationale for control group(s).
Not applicable ☐

(Max ¼ page)

There have been no experimental studies of this housing model in Canadian settings. In order to influence policy we need a strong design to find out whether this intervention works, at what cost and for whom in our local contexts. In order to determine which models work best for whom, we need to compare people who are receiving housing with supports with people who are not receiving these services. Individuals in the Usual Care Group will be offered referrals to existing services and will not be placed at any greater risk of harm than they face in their current circumstances. Toronto has many services to help homeless people including housing help and referral centres that help people find housing, street outreach services, emergency shelters, drop ins, and social (subsidized and supportive) housing. There is also a broad range of agencies that provide health care for homeless people. This study is making additional resources available to some individuals in a fair and transparent manner (randomization) in order to test their effectiveness.

Please see 16A below and *Appendix L* for more information about usual care available to study participants

15D. Indicate how many participants will be enrolled.

**Total study enrollment** 560	
**Number of participants to be enrolled at this site** n/a	**Or Total Number of charts to be reviewed at this site?** Charts selected for review will be based on the most frequently used sources of health care (such as primary care physicians, community health centres and emergency departments) by participants in the 3 years prior to study enrollment. It is possible that some charts may be reviewed at St. Michael's Hospital but the exact number is unknown at this time.
**Time period for enrollment:** Enrollment will begin in October 2009 and is expected to last 18 months.	
**Approximate size of eligible population from institution/practice:** According to the City of Toronto's Street Needs Assessment, a minimum of 5,052 individuals were estimated to be absolutely homeless throughout Toronto on April 19, 2006.	

15E. Is sample size justified in the protocol? ☒ Yes ☐ No

If **YES**, indicate protocol page: **page 11**
If **NO**, provide sample size justification.

(Max ¼ page)

16. STUDY INTERVENTIONS OR PROCEDURES

Not Applicable ☐ (e.g. observational studies). If not applicable, go directly to section 17 (Data Analysis)

16A. Document the usual standard of care at this institution for this population.

Not Applicable ☐

(Max ¼ page)

Homeless individuals with serious and persistent mental health issues may access a broad collection of health and social services available in Toronto that includes: inpatient and outpatient clinical service, peer support, crisis services, intensive case management, Assertive Community Treatment Teams (ACT), social housing, supportive housing, vocational programming, street outreach programs, drop-in services and shelters.

Participants in the Usual Care Group will still be able to access the broad range of services available in Toronto for homeless people with mental health issues. Participants in this group will be provided with information about available services in the community.

It is recognized that some individuals in the usual care group may over time, through new or existing programs, access services that are similar to the experimental intervention. Health and social services use will be measured through the common protocol of health service use and included in the analyses of process and outcomes.

For more details, please see the Study Protocol: Appendix B and Appendix L: Study Intervention and Usual Care Group Details.

16B. What procedures will be carried out in the study that are not considered part of the diagnostic, therapeutic "routine" or standard of care? Attach a copy of all non-standardized instruments (e.g., questionnaires, rating scales).

(Max ¼ page)

Research activities (i.e. data collection) for both the Usual Care and Intervention Groups are the same.

The experimental intervention includes the development and implementation of three new services: a recovery-focused Assertive Community Treatment (ACT) team; and two Intensive Case Management (ICM) teams, one with a focus to serve homeless people from ethno-racial visible minority groups. Each of these teams will serve 100 participants and will based at community-agencies. In addition, participants in the experimental intervention will receive a rent allowance of $600 per month and support to obtain immediate access to housing.

Attempts will be made to house individuals within one month or to provide immediate temporary accommodation until suitable housing can be secured. Participants will be allowed to choose their own housing after it meets minimum City of Toronto standards for acceptability, including property standards, affordability. First/last months rent, furniture, eviction prevention funds have been budgeted and will be available to participants. The rent allowance will be guaranteed until the end of March 2013. The City of Toronto's Shelter, Support and Housing Administration Division will administer the rent allowances (directly to landlords so that participant social benefit incomes are not negatively impacted) and provide housing support to the ICM and ACT teams. Most housing will be in private market units. Participants will be placed in social housing only if they are already at the top of the social housing wait list.

For more details, please see the Study Protocol: Appendix B and Appendix L: Study Intervention and Usual Care Grou Details.

16C. Indicate the additional risks associated with the study as compared to usual standard of care. Do not refer to other sections of this form.

(Max ½ page)

There are no additional risks associated with the study as compared to usual standard of care.

16D. Indicate duration of study visits and extra time commitment (length, number, and frequency of test sessions) for study participation.

(Max ½ page)

Total study duration is 2 years.

For participants in the usual care group: monthly 5-minute check-ins by phone, one 80-minute baseline survey interview followed by three 70-minute interviews (at 6 months, 12 months and 18 months), four 10-minute interviews (at 3 months, 9 months, 15 months and 21 months) and one 75-minute final interview.

For participants in the intervention group: same as above, plus weekly visits by case manager or member of ACT team.

A sub sample (N=50) of participants from both groups will be invited to participate in 2 in-depth qualitative interviews (one at baseline and one at 18 months) for approximately 90-120 minutes each interview.

17. DATA ANALYSIS

Briefly explain what methods will be used to analyze study data.
References to protocol for this question are acceptable. Indicate applicable page(s) of protocol.

(Max ¼ page)

Quantitative Data
Differences in the intervention and control groups will be examined by conducted performining t-tests and chi-square analyses at the individual level on a range of baseline outcomes and other demographic and risk factor characteristics. Power will be calculated for a range of effect sizes and intra-class correlations for the implemented longitudinal experimental design. Multi-level models will be implemented to estimate program effects and to study the trajectories of outcomes as a function of both between individual and time varying covariates. Robustness of results will be evaluated through propensity scoring methods used to match individuals in the treatment group with individuals from the control group on a number of baseline measures.

Because the Toronto sample alone is likely to be underpowered to address if the intervention has had differential impacts for each of the groups, we will work closely with the Mental Health Commission of Canada's national research team to implement a group-based approach for analyzing developmental trajectories. This methodology applies finite mixture modeling to identify groups of individuals following similar trajectories on a variable measured (e.g. housing stability) repeatedly over time.

Analysis of data obtained from health care and social service databases in key areas will include: (1) identification and costing of all inputs (ii) identification and costing of outputs (iii) quantification of outcomes (iv) comparison of costs with outcomes (v) direct and indirect benefits of the intervention (based on program theory) (vi) potential short, immediate and long term benefits of the program.

Qualitative Data
The personal narratives from the subset of 50 individuals will be explored using qualitative longitudinal research methods with a focus on explaining the pathways by which the intervention affect the outcomes. Qualitative data analysis will follow a systematic, comparative grounded theory method of analysis. Line-by-line coding of transcribed text from the in-depth interviews will identify key concepts and will be facilitated by the constant comparative method involving the comparison of themes and concepts as the analysis progresses.

Please see Appendix B: Study Protocol for more details.

SECTION III: ETHICAL ISSUES

18. RECRUITMENT AND CONSENT

Any document to be viewed by a study participant (e.g., recruitment posters/letters, consent/assent forms, information sheets) must be included with your submission.

18A. What tools will be used to identify potential participants for recruitment into the study?

☐ Permanent health record/clinical chart (specify source):
☐ Existing database (specify):

- o Does the Principal Investigator maintain the database? ☐ Yes ☐ No
- o If No, identify the entity that maintains the database:

Note: The creation and maintenance of a database for research purposes is a research activity that may require a separate REB application. Consult your institutional REB.

☐ Advertisements, including web based recruitment tools (attach)

☒ Other (specify):

Recruitment to the study may come from several points of entry. Toronto's extensive network of organizations that serve homeless people will be a major source of referrals. Any health or social service agency or organization will be able to refer persons to the intake coordinator for assessment of eligibility. This network includes but is not limited to: shelters, drop-in centres, street outreach programs, housing help offices, community health care centres, and emergency departments. To maximize the appropriateness of referrals to the project and to ensure that the entire range of potentially eligible individuals from across Toronto are referred to the project, the research team has convened a Referrals Work Group made up of representatives of likely referral sources (e.g. the Toronto Drop-in Network, the ER Alliance, the Shelter Network). Communication materials have also been developed for targeted distribution to potential referral sources to ensure common understanding of the study's target population and eligibility criteria and appropriate referral. In an effort to minimize potential participant vulnerability and therapeutic misconception, referrals by individuals will not be accepted. People who self-refer or individuals who are not affiliated with health or social service agencies but who would like to refer potential participants, will be directed to agencies on the study's referral work group who will ensure that the potential study participant is connected to existing social services and, if appropriate, will then refer this person to the study.

Please see Referral Poster & Information Sheet in Appendix G.

18B. Who will identify potential study participants?

☐ Investigator/study personnel
☒ Other healthcare professional (e.g. non-study personnel)
☐ Self-referral (e.g. response to advertisement)

18C. Who will make initial contact with potential participants or an authorized third party? Is this individual (s) already known to the participant or authorized third party? How will contact be made (e.g., in person, phone, letter, e-mail, website)? Attach a copy of the script or any written materials if applicable. Not Applicable ☐

(Max ¼ page)

Initial contact will be made in person primarily by health and social services providers who will have been provided with study information and referral materials including a script for approaching potential participants about the study. Referrals will be made to the study intake coordinator who will work on a mobile basis to meet with individuals who have been identified as potential participants. Once referred to the study, potential subjects will be assessed in-person by the study intake coordinator who will establish eligibility and obtain consent. Assessments will take place at a safe location (possibly the established project office where the intervention service providers will be located) that is convenient to the potential subject.

If a potential participant is screened but found to be ineligible for any reason (e.g. not 'absolutely' homeless, already receiving ACT or ICM services within the last 8 weeks), they will be able to be re-screened only once every 3 months while enrollment to the study is still open. Once someone has been enrolled to the study, they cannot be re-screened.

Please see Referral Information for Service Providers: Appendix G and Referral Information for Participants: Appendix H.

18D. Describe the consent process (e.g. will consent be written, oral, telephone (include script). If the study population requires special consent considerations (e.g., child, incompetent adult, unable to communicate), refer to 18E.
Not Applicable ☐

(Max ¼ page)

Because the screening form collects personal health information which is necessary to determine study eligiblity, a verbal consent process will take place prior to the screening interview. The verbal screening consent process will not replace the written consent process (described below) for the study itself. However, because the study involves explaining multiple possible study assignments (i.e. high needs intervention, moderate needs intervention, ethno-specific intervention, and care as usual groups), we have decided to conduct the full study written consent after eligibility has been determined so as not to unnecessarily burden potential participants who may or may not be eligible for the study with an additional hypothetical situation (i.e. that they may or may not even be eligible to consider the full range of study participation assignments).

Written informed consent will be obtained from participants after they have been screened and deemed eligible for the study. Consent forms are crafted in an accessible, plain language format. To further clarify the study and ensure informed consent, additional consent measures (study questionnaire and overview) have been created and will be included in the consent process. As well, the Study Information and Consent Form will be read aloud to ensure that all participants, regardless of literacy level, will have been given the information required for them to be able to give informed consent. For the qualitative sub-study to collect personal narratives a separate written consent process will take place. Throughout this study, consent will be an ongoing process that ensures that the patient is kept informed and consents to participate at all stages of the research process.

See Appendix R and 18E for additional measures that will be taken to ensure consent with this population. See Appendix I: Information and Consent Form: Main Study and Appendix N: Information and Consent Form: Personal Narrative Component.

i) Who will obtain consent?

(Max ¼ page)

The study intake coordinator will obtain consent. The study intake coordinator (to be hired) will have clinical intake and assessment skills and/or experience recruiting and interviewing homeless and marginally housed individuals.

ii) Is there is a relationship between the participants and either of the following:

Person obtaining consent ☐ Yes ☒ No
Investigator ☐ Yes ☒ No

iii) If YES, explain the nature of the relationship (e.g., physician, employer) and what steps will be taken to avoid the perception of undue influence.

(Max ¼ page)

iv) How much time will be given to participants to review the information before being asked to give consent?

(Max ¼ page)

30 minutes will be allotted for each subject to review the study and consent information with more time given if needed.

18E. Does your research involve any of the following:

i) Special Considerations (check all that apply):

☒ Women of child bearing potential
☒ Pregnant women
☐ Healthy volunteers
☐ Students
☐ Staff
☐ Genetic research
☐ Tissue samples
☐ Fetal tissue or placenta
☐ Prisoners
☐ Participants unable to communicate
☐ None of the above
☐ Other, specify

ii) Capacity/Competency (check all that apply):

☐ Children
☐ Emergency patients
☐ Individuals temporarily unable to assent
☐ Individuals who lack the capacity to assent.
☒ None of the above (skip to Question 18Eiii)

Describe how capacity will be assessed for any individuals in 18Eii.

(Max ¼ page)

If participants are incapable of providing consent, how will substitute decision-makers be identified?

(Max ¼ page)

N/A. We will not accept consent via substitute decision makers. Potential participants who are incapable of providing consent will be excluded from the study.

When inability to provide an informed consent is expected to be temporary, describe what procedures will be used to regularly assess capacity and to obtain consent if the individual later becomes capable of providing consent.

(Max ¼ page)

Participants will be enrolled in the study only if they are able to give informed written consent. Substitute decision makers and/or assent will not be accepted. Having a mental illness does not automatically make an individual incapable to consent to treatment or to participation in research studies [9]. Several studies have shown better decisional capacity among individuals with severe mental illness, even while actively symptomatic, then previously believed [10-12]. Nevertheless, it is acknowledge that some potential participants may be compromised in their ability to provide consent or that this may fluctuate over time.

In an effort to alleviate any lack of understanding of study involvement, we propose to take some extra educational and communicative steps which have been recommended in the literature [13,14]:

- use a simple, clear and repeated informed consent process
- document observations about understanding of participation at the time of consent

If a potential participant is temporarily unable to provide consent at the time of their initial referral (e.g. due to intoxication, acute exacerbated psychosis, acute medical illness), the study intake coordinator will ask the participant to contact the research coordinator at a later date.

iii) Communication Difficulties (check all that apply):

☒ Individuals who may require translation
☒ Individuals who are illiterate.
☐ Participants who have trouble understanding and/or producing speech (and require special support including the use of assistive devices)
☐ None of the above (skip to Question 18F)

Provide an explanation of what procedures will be used to address any communication difficulties (e.g., the use of translated forms, translator, impartial witness).

(Max ¼ page)

Where potential subjects do not speak English, we will hire professional and certified translators who are competent in the required languages. In these cases, the study information and consent will be verbally translated. Translators will be asked to sign a declaration that the study was adequately explained and that

consent was freely given.

The study may also involve participants who are illiterate or have low literacy levels. Consent forms are crafted in accessible, plain language and will be read aloud and explained to all survey participants, regardless of literacy level. For participants who are unable to write, consent will be given orally and a witness will be present during the entire process to ensure that all aspects of the consent process are understood and that consent is given freely. A witness declaration and signature line has been included as part of the consent form.

Please see Consent Form: Appendix I.

18F. What steps will be taken to determine if the participants are already enrolled in other studies or are likely to be enrolled in other studies during the term of this study?

(Max ¼ page)

Enrollment in other studies is not likely to be an issue in this subject population.

SECTION IV: RISKS, BENEFITS AND SAFETY

19. RISK/BENEFIT ESTIMATES

19A. Potential Harms (injury, discomfort and inconvenience) to participant (including psychological factors).
No known risks ☐

i) List the known risks of study intervention(s) including approximate rates of occurrence, severity and rates of reversibility.

(Max ¾ page)

Subjects may experience some discomfort as a result of being asked about personal health experiences and/or life events, which may have been painful. Any potential vulnerability of participants will be minimized since study findings will be kept confidential, anonymized and participants are not required to answer any questions that they do not want to and they may stop the interview at any time. A referral/resource list will also be part of each interview kit so that interviewers will be able to make referrals to appropriate community services if participants require support during or after the interview process.

Individuals who are randomized into the usual care group may feel some disappointment over not receiving the intervention benefits (housing and supports). This will be minimized by providing all members of the Usual Care Group with information about available services in the community and they will be welcome to use the field office (where the intervention service providers will be located as well as some research staff) as a resource centre.

This study proposes to use multiple and varied data sources (including charts, Ministry of Health data, and primary data collection) for each participant. This comprehensiveness is critical to the scientific objectives of the study, yet may increase invasion of individual participant privacy. This privacy concern will be clearly communicated to potential participants, as will the measures for protection of security and confidentiality, prior to consent. Individuals who decline permission for linkage to health, social service and justice data bases will still be eligible to participate in the study. *Data protection measures are further outlined in Section V and in the Study Protocol: Appendix B.*

ii) List the risks of any tests, procedures or other protocol-mandated activities that are conducted for research purposes only, including approximate rates of occurrence, severity and reversibility.

(Max ¾ page)<br>N/A.

iii) For studies involving placebo, washout, or withholding treatment, list any risks related to withdrawal or absence of treatment.

Not Applicable ☒

(Max ¾ page)

iv) Include a summary of the data regarding reproductive risks such as teratogenicity or embryotoxicity of the study drug, any risk with breastfeeding, or risk to men regarding conception.

Risks unknown ☐

(Max ¼ page)<br><br>N/A

v) Does participation in this study affect alternatives for future care?

☐ Yes ☒ No

If **YES**, explain.

(Max ¼ page)

19B. Potential Benefits to Participants

No direct benefits anticipated ☐

List anticipated benefits to the participant, if any.

(Max ¼ page)<br><br>Subjects who are randomized to the intervention group (N=300) will receive a rental allowance ($600 per month) plus support services (either an Assertive Community Treatment team or Intensive Case Management) until March 2013 and possibly beyond (if continued funding becomes available).

20. REMUNERATION

Not Applicable ☐

What payment(s) will be provided to participants or substitute decision makers (if applicable)?

☐ Reimbursement for expenses incurred as a result of research<br>Amount: Specify (e.g., travel, meals): Subjects will be provided with TTC tokens if travel to interview location is necessary.
☐ Gifts for participation<br>Value:
☒ Compensation for time<br>Amount: Between $10 to $30 (depending on length of interview)<br>Provide justification if compensation for time will be provided. (Max 1/4 page): Subjects will be paid an honorarium each interview (lasting between 10 minutes and 1 1/2 hours) to recognize the value of their time, to encourage follow up and to help reduce barriers to participation.
☐ Other forms of compensation:

21. SAFETY MONITORING

21A. Is there a safety monitoring plan for the study?
☒ Yes ☐ No ☐ Not Applicable

If **YES**, provide details. If **NO**, justify.

(Max ¼ page)

Procedures will be in place to ensure the security and safety of both participants and interviewers. This will occur through interim analysis of data (see 21B) and through our interview safety protocol. *Please see Appendix S: Safety Protocol.*

21B. Is an interim analysis planned? ☒ Yes ☐ No ☐ Not Applicable

If **YES**, describe briefly.

(Max ¼ page)

Service utilization and participant survey data will be analyzed to examine differences in treatment and control groups at the study's half way point. In the areas of safety monitoring, we will examine whether there have been any systematic negative consequences of the trial. We will have protocols in place to guide acute safety events (e.g., eviction from housing).

21C. Is there a steering committee? ☒ Yes ☐ No ☐ Not Applicable

NOTE: If **YES**, attach a copy of the terms of reference (mandate) of the steering committee.

Please see Appendix J: Site Operations Team Terms of Reference.

21D. Is there a Data and Safety Monitoring Board (DSMB)?
☐ Yes ☐ No ☒ Not Applicable

If **YES**, forward a copy of the DSMB charter when available or provide a description of the DSMB, including its purpose, membership, relationship to the sponsor, and whether the committee will review unblinded study data etc. **Refer to the protocol as needed.**

(Max ¼ page)

Is the DSMB independent of the sponsor? ☐ Yes ☐ No

If **NO**, justify and explain what alternative arrangements are in place to monitor the safety data and how the overall risk/benefit information will be communicated to the REB.

(Max ¼ page)

22. PUBLICATION/DISSEMINATION OF RESULTS

Indicate how the results will be communicated to participants and other stakeholders (e.g., advocacy groups, scientific community).

TO PARTICIPANTS:	TO OTHER STAKEHOLDERS:
☐ Individual debriefing at end of test session	☒ Presentation
☐ Group debriefing	☒ Publication
☐ Letter of appreciation at end of study	☒ Other (specify): community forum at end of study and periodic newsletter updates. Only group-level de-identified data will be presented.
☐ Publication	☐ No Plan, justify below.
☒ Other (specify): Participants will be invited to the community forum at the end of the study. Only group-level de-identified data will be presented.	
☐ No Plan, justify below.	

If no plan is in place, provide justification.

Not Applicable ☐

(Max ¼ page)

SECTION V: PRIVACY AND CONFIDENTIALITY

23. COLLECTION, USE AND DISCLOSURE OF PERSONAL HEALTH INFORMATION

Investigators should comply with the duties set out for researchers in the Personal Health Information Protection Act (PHIPA), with the privacy and confidentiality and consent guidelines outlined in the Tri-Council Policy Statement on Ethical Conduct for Research Involving Humans and other requirements and guidelines as per the TAHSN Principles for Development of Policy and Guidelines on Security of Personal Health Information Used for Research Purposes (February 4, 2008).

It is a requirement of the institution and PHIPA that a **complete information access log** be kept for each study and for the duration of the study to identify **all personnel** who have access to personal health information for research purposes. The REB or the Institution may require access to this log as part of the monitoring process or for investigational purposes. This log must be kept as part of the recruitment and study conduct processes.

23A. Identify all persons including non-institutional service providers, that will have access to the personal health information now or in the future, their roles in the study (e.g., chart review), their reason for access (e.g. eligible study recruits), and related qualifications.

Only a partial list is provided below. For the complete list of persons with access to personal health information, please see Appendix Q.

1	Title: Co-Principal Investigator	Last Name: Hwang	First Name: Stephen
	Institution: St. Michael's Hospital	Qualifications: MD, MPH, FRCPC	Role in Study: Study design, data analysis
	Reason for Access (e.g. recruitment, study conduct, other – specify): As a co-principal investigator of the study, Dr. Hwang will be involved in all aspects of the study and therefore requires access to the personal health information being collected.		

2	Title: Co-Principal Investigator	Last Name: Stergiopoulos	First Name: Vicky
	Institution: St. Michael's Hospital	Qualifications: MD, MHSc, FRCPC	Role in Study: Study design, data analysis
	Reason for Access (e.g. recruitment, study conduct, other – specify): As a co-principal investigator of the study, Dr. Stergiopoulos will be involved in all aspects of the study and therefore requires access to the personal health information being collected.		
3	Title: Co- Principal Investigator	Last Name: O'Campo	First Name: Patricia
	Institution: St. Michael's Hospital	Qualifications: PhD	Role in Study: Study design, data analysis
	Reason for Access (e.g. recruitment, study conduct, other – specify): As a co-principal investigator of the study, Dr. O'Campo will be involved in all aspects of the study and therefore requires access to the personal health information being collected.		
4	Title: Research Coordinator	Last Name: Mason	First Name: Kate
	Institution: St. Michael's Hospital	Qualifications: MHSc	Role in Study: Study implementation
	Reason for Access (e.g. recruitment, study conduct, other – specify): This access is necessary for study coordination, recruitment and data collection purposes.		
5	Title: National Research Lead	Last Name: Goering	First Name: Paula
	Institution: Centre for Addiction and Mental Health/University of Toronto/Mental Health Commission of Canada	Qualifications: MSN, PhD	Role in Study: National Research Team Principal Investigator, multi-site study design
	Reason for Access (e.g. recruitment, study conduct, other – specify): Leads and manages the overall multi-site study at the national level. Access to Toronto study data is necessary for cross-site comparisons and grouped multi-site analysis.		

23B. Has your research team been given training in privacy and confidentiality issues for this study?
☐ Yes ☒ No

If **NO**, when will training be provided?

(Max ¼ page)

Training on confidentiality and data security will be part of study training and will take place in September 2009. All research team members and staff will have to declare their understanding of and compliance with central and site specific policy and procedures related to confidentiality and privacy.

23C. Is someone on the research team responsible for the protection of privacy and confidentiality?
☒ Yes ☐ No

If **YES**, please provide:

Last Name: Hwang	First Name: Stephen
Position: Principal Investigator	Contact Information: hwangs@smh.toronto.on.ca

23D. List the identifying and identifiable information that will be collected, used, or disclosed from the records during the course of the <u>proposed recruitment activities</u>. The box below lists the most common personal identifying information that might be collected, used and disclosed (see the TAHSN Guidelines for completing this application for a more complete list). Please check the applicable boxes below or add additional identifying information.

☒ Name	☐ Images (e.g., photographic, x-ray, MRI scans)
☒ Address	☐ Social Insurance Number
☒ Telephone/Fax Numbers	☐ Medical Record Number
☒ Email Address/IP Address/URLs	☐ Date of Birth
☐ Health Card Number	☒ Health Information: (e.g., relating to inclusion /exclusion criteria, medications)
☐ Other information (specify):	

23E. Describe the security measures that will be taken to protect the confidentiality of this information.

(Max ¼ page)

Referrals will be made to a single individual (the study Research Coordinator) who will store any information collected for the purposes of recruitment in a locked filing cabinet and on a password protected computer. Access to this information will be restricted to this individual and the study intake coordinator.

The data collection form used for referral information is attached as Appendix G.

23F. What will happen to this information at the completion of the recruitment process?
NOTE: If information will be destroyed, provide the name of the person responsible and at what point the destruction will occur

(Max ¼ page)

As part of the study we intend to analyze referral and screening data to understand more about who was not eligible or decided not to participate in the study. Verbal permission will be obtained from participants for permission to keep referral and screening data. *See Appendix O for consent script.* All referral data will be retained for a period of 10 years after the study has been closed and will subsequently be destroyed by Stephen Hwang, Principal Investigator.

<u>24. DO YOU KEEP A LOG OF PERSONNEL</u> who have access to personal health information for recruitment purposes? ☒ Yes ☐ No

<u>25. LIST ALL PERSONAL HEALTH INFORMATION AND PERSONAL IDENTIFIERS</u> (e.g. name, DOB) required to be collected. For all non-clinical trials, attach data collection forms.

(Max ¼ page)

Personal Health Information: Study participants will be asked to answer closed and open-ended question about their physical and mental health and well-being. Information on health care utilization, prescription drug use and access to health care will also be collected through self-reported survey questions and data linkage. Physiological health measures (blood pressure, height/weight, hip/waist) will be obtained from participants at 5 points in the study. Data collected from chart review will focus on diagnosis data, preventative health measures (screening and treatment), and select physiological measures where available. *See Appendix C: Screening Tool; Appendix D: Survey Instruments; Appendix F: Chart Abstraction Guide.*

Personal Identifiers: Name, date of birth, provincial health card number, Ontario Disability Support Program (ODSP) or Ontario Works (OW) number will be collected at baseline.

These personal identifiers are needed for the purposes of linkage with provincial health care databases through the Institute for Clinical and Evaluative Sciences (ICES), records kept by the Correctional Service of Canada, the Ministry of Community and Social Services, Ministry of Community Safety and Correctional Services, the Toronto Police Service and City of Toronto databases for the purpose of determining cost effectiveness and outcomes of the intervention through an analysis of health care and social services utilization (ambulatory visits, emergency departments, income benefits, justice system contacts, shelter use). The main purpose of the study is to ascertain whether housing and support services are associated with changes in physical and mental health functioning, housing stability and the use of health care services, hence the study cannot be conducted without collecting personal health information.

Please note: all identifiers will be removed once the data has been verified and a master list linking data with identifiers will be segregated and stored separately from the data.

25A. Identify all potential sources of this information.

☒ Permanent health record/clinical chart (specify source): Participant's health care provider(s)
☒ Existing database (specify): ICES, City of Toronto Shelter Utilization, Ministry of Community & Social Services (MCSS), Correctional Service of Canada (CSC), Ministry of Community Safety and Correctional Services (MCSCS), Toronto Police Service.<br>o Does the Principal Investigator maintain the database? ☐ Yes ☒ No – ICES: PI has access as adjunct scientist; City of Toronto: No; CSC: No; MCSS: No; MCSCS: No; Toronto Police Service: No. *[Note: data agreements are in progress and will be in place with each institution/database provider]*<br>o If No, identify the entity that maintains the database: ICES/SHSC; City of Toronto Shelter, Support & Housing Administration Division, MCSS, CSC, MCSCS, Toronto Police Service.
☒ Directly from the participant
☐ From other institutions Specify:
☐ Other (specify):

25B. Indicate how study participants will be identified on data collection forms (e.g. study number, initials).

☒ Participant Identification #
☐ Other (specify:)<br>If using other, please justify:

25C. Indicate how data will be stored.

☒ Computerized files<br>(Specify): Server ☒ Desktop ☐ Laptop ☐<br>Server (specify): Internal ☒ Contracted Service Provider ☒ Other Third Party ☐
☒ Hard copy
☒ Audio recordings
☐ Video recordings
☐ Other (e.g. PDA, USB and other portable storage devices):

25D. Indicate where the data will be stored.

☒ On-site
☒ Off-site, Specify location(s): Toronto, ON
If off-site, will a back-up copy be stored on site? ☐ Yes ☒ No If "no" justify:

Consent forms will be kept on-site in locked cabinets with restricted access at the Centre for Research on Inner City Health. A master list linking consent forms with data will be stored separately.

All quantitative survey data will be entered directly into laptops configured specifically for the project and maintained by HealthDiary, the data vendor contracted by the Mental Health Commission of Canada to manage data storage for the study. No data will be stored on the laptop's local hard drive and no hard copies of this data will be kept. Data will be stored by HealthDiary at an off-site centralized server with high levels of physical and network security,

redundant power supply, redundant internet connections and power supply. All data will be encrypted and protected with strong passwords and authentication. Data will be backed up daily to a secure storage location at the data centre. Physical access to these backups is highly restricted and backups are password protected. Data backups will also be taken offsite from the data centre by a secure transport service to a secure storage location provided by Iron Mountain.

Qualitative interview data will be collected using digital recorders, transcribed and uploaded to the central data vendor's off-site secure server for storage. Paper transcripts of interviews will be identified by code number and stored in a locked filing cabinet. An additional written consent process will be obtained for this part of the study with selected participants.

Chart review data will be stored on St Michael's Hospital servers and transferred using encrypted USB keys. Data from chart reviews will be entered directly onto laptops configured specifically for this aspect of the study and maintained by the Centre for Research on Inner City Health with multiple password protection and data encryption software. A master list linking data with identifiers will be stored separately from the data.

25E. Indicate which of the measures will be undertaken to protect the confidentiality and security of the data, including any physical and technical safeguards

☒ Data stored on mobile devices will be encrypted
☒ Data will be password protected
☒ Data will be stored on a hospital or other institutional network drive that has firewalls and security measures in place
☒ Hard copy records will be stored in a locked cabinet in a secure location
☒ Access to records and data limited to authorized persons
☒ Study data will be **de-identified or coded.** A master linking log with identifiers will be kept and stored separately from the data.
☐ Study data will be **anonymized.** All identifiers will be removed once the data has been collected/verified/analyzed.
☐ Study data will be **anonymous.** Identifiers/identifying information will not be collected
**If audio/video recordings will be used:**<br>☒ Tapes will be destroyed upon transcription/review/verification/analysis<br>☒ Tapes will coded<br>☐ Tapes will not capture date and time
☐ Other:

25F. Indicate who will have access to data in the future

(Max ¼ page)

Access to the data will be limited to authorized users only, using a multi-level, permissions protocol that will specify roles and types of data access permitted, using the need to know principle. The research team, comprised of the Co-Principal Investigators and Co-Investigators listed in 23A, will have access to de-identified data. Research Coordinators and Assistants with specific roles such as participant follow-up or data review and management will have access to the data for such specified purposes. St. Michael's Hospital Research Ethics Board will have access the data for monitoring purposes.

25G. Indicate if any information that could potentially identify study participants will be disclosed outside of the custody of the Health Information Custodian (Hospital or responsible institution) (e.g., names, initials, DOB, OHIP #).
☒ Yes ☐ No

If **YES**, to whom?

(Max ¼ page)

Identifiable data (names, DOB, OHIP #, Ontario Works or Ontario Disability Support Program #) will be shared with the Institutes for Clinical Evaluative Sciences, the Ministry of Community and Social Services, Correctional Service of Canada, the Ministry of Community Safety and Corrections, the Toronto Police Service and the City of Toronto's Division of Shelter, Support & Housing Administration.

Is there a contract or agreement in place that requires the transfer of data from the custody of the Health Information Custodian? ☐ Yes ☒ No

Justify and describe how this information will be transferred and any security measures to be used (e.g., de-identified data, secure network upload or download).

(Max ¼ page)

This transfer of information is necessary for data linkage. Agreements with all of the agencies listed in 25G have begun and will be in place before any data is transferred. Data will be transmitted as encrypted files via secure network upload or by physical transfer (courier) on encrypted USB keys.

25H. If personal health information is to be linked to other databases (e.g., health registries, statistics Canada information) provide the following details:

Not Applicable ☐

Describe the data to which the personal health information will be linked.

(Max ¼ page)

Data linkages are for the purposes of the study only and are not permanent linkages with the database owners.

- Health card number, name and date of birth will be used to obtain health care utilization and prescription drug use data from the Ministry of Health.
- Name, date of birth and ODSP/OW number will be used to obtain Ontario Disability Support Program (ODSP) and Ontario Works benefit amounts and disability diagnosis from the Ministry of Community and Social Services.
- Name and date of birth will be used to obtain justice system contacts from the Toronto Police Service, the Ministry of Community Safety and Correctional Services and the Correctional Service of Canada.
- Name and date of birth will be used to obtain shelter utilization data from the City of Toronto Division of Shelter, Support and Housing Administration.

ii) Explain how the linkages will be made.

(Max ¼ page)

Agreements are in process to obtain the above data from the Institute for Clinical Evaluative Sciences, the Ministry of Community and Social Services, Correctional Service of Canada, the Ministry of Community Safety and Correctional Services, the Toronto Police Service and the City of Toronto.

iii) Explain why these linkages are required.

(Max ¼ page)

These linkages are required for the purpose of determining cost effectiveness and outcomes of the intervention through an analysis of health care and social services utilization (ambulatory visits, emergency departments, income benefits, shelter use).

25I. Indicate how long the personal health information will remain identifiable and explain why.
Not Applicable ☐

(Max ¼ page)

All identifiers will be removed once the data has been collected and verified. A master list that links data with identifiers will be segregated from the data and securely stored. Identifiers must be kept for data linkage purposes.

25J. Explain why the research cannot reasonably be accomplished without using personal health information.

(Max ¼ page)

The purpose of the study is to ascertain whether housing and support services are associated with changes in such things as physical and mental health functioning, and the use of health care services, hence the study cannot be conducting without using personal health information.

25K. If personal health information will be collected, used or disclosed without consent from the individuals to whom the information relates, explain why obtaining explicit consent would be impractical.

(Max ¼ page)

n/a

25L. Describe any harms that could arise if personal health information was inappropriately released (e.g., embarrassment, refusal of employment or insurance coverage, stigmatization of individuals / groups) and how any consequences would be addressed.

(Max ¼ page)

Embarrassment and stigmatization could arise if personal health information from this study was inappropriately released. If such an event were to occur, all affected persons would be notified and efforts would be made to address any consequences and/or provide support to address any specific harms.

25M. Describe how and when the personal health information will be disposed of or returned to the health information custodian.

(Max ¼ page)

Personal health information will be retained for a period of 10 years after the study has been closed and will subsequently be destroyed. Paper records will be shredded. USB / hard drive storage devices will be securely erased using tools that meet the minimum RCMP standards for data destruction. Any tapes will be physically destroyed.

SECTION VI: FUNDING, CONTRACTS AND AGREEMENTS

26. BUDGET

26A. Attach an itemized study budget (applies to all full board and delegated review studies). The budget should reflect all costs to complete the study (e.g. database extraction, student payments, participant reimbursement etc.)
OR ☐ No budget required; see explanation in Section 3.

26B. Is funding sufficient to cover all study costs? ☒ Yes ☐ No

If **NO**, explain how the shortfall will be made up.

(Max ¼ page)

26C. Will any investigator receive direct personal payments? ☐ Yes ☒ No

If **YES**, describe what these payments are for and the amount.

(Max ¼ page)

27. CONTRACTS AND AGREEMENTS

"In many clinical trials, the sponsors may obtain contractual rights to the initial analysis and interpretation of the resultant data. Researchers and REBs must ensure, however, that final analysis and interpretation of such data remain with the researchers, whose duty it is to ensure the integrity of their research." (TCPS 7E)

REBs also legitimately seek assurances that other contractual rights and obligations are consistent with the statements in the protocol. This is why the REB requests information regarding agreements related to transfers of personal information and biological material (for privacy issues), liability (to ensure that participant reimbursement is appropriately available) and publication. Review by the institution ensures that certain institutional policies are met.

27A. Contract/Research Agreement
Is there **any party** external to the institution involved with the research that will be entering into an agreement or contract with the institution? ☒ Yes ☐ No

If **YES**, provide names and roles of those involved (i.e. Regulatory sponsor, contract research organization, funder, collaboration institution, vendor or researcher).

(Max ¼ page)<br><br>Mental Health Commission of Canada: funding and research collaboration agreement.<br>Institute for Clinical Evaluative Sciences: Data sharing agreement in process.<br>Ministry of Community and Social Services: Data sharing agreement in process.<br>City of Toronto's Division of Shelter, Support and Housing Administration: Data sharing agreement in process.<br>Correctional Service of Canada: Data sharing agreement in process.<br>Ministry of Community Safety and Correctional Services: Data sharing agreement in process.<br>Toronto Police Service: Data sharing agreement in process.

27B. Has the contract/research agreement has been submitted for review and signing (see institution specific instruction page)? ☒ Yes ☐ No

27C. Transfer Agreement
Will biological materials (e.g. blood, other bodily fluids, tissues) or identifiable information (e.g. data, video and audio and other data) be transferred? If so, has an agreement related to the transfer (e.g., Material Transfer Agreement, Information Sharing Agreement, Service Provider Agreement, Vendor Agreement) been approved?
☐ Yes ☒ No

If **NO**, explain.

(Max ¼ page)

28. LIABILITY

Who will cover reasonable out-of pocket expenses to ensure that immediate medical care is provided if a participant suffers an injury as a result of participation in the study?

- ☐ Regulatory Sponsor (as listed above, Section 8C)
- ☐ Funder
- ☒ Institution
- ☐ Other (specify):

29. PUBLICATION

Has the funding agency or sponsoring company placed any restrictions on publication of findings (e.g., timing of manuscripts; approval process of manuscripts) or on reporting interim results? ☒ Yes ☐ No ☐ Pending

If **YES**, explain any restrictions.

(Max ¼ page)<br><br>Publications that include data from other sites (i.e. Vancouver, Winnipeg, Montreal or Moncton) or that overlap with the list of core cross-site papers emerging from the multi-site study must be approved by a committee of national team researchers and representatives from each site. There are no restrictions placed on data collected exclusively in our Toronto study.

Appendix A: REB TAHSN Form References

1. City of Toronto, Shelter, Support and Housing Administration Division. 2006. *2006 Street Needs Assessment: Results and key findings.* Toronto: City of Toronto. Available from: www.toronto.ca/housing/pdf/streetneedsassesment.htm.
2. Fischer P and Breakey W, *The epidemiology of alcohol, drug and mental disorders among homeless persons.* American Psychologist, 1991. 46: p. 1115-1128.
3. Koegel P, Burnam M, and Farr R, *The prevalence of specific psychiatric disorders among homeless individuals in the inner city of Los Angeles.* Archives of General Psychiatry, 1988. 45: p. 1085-1092.
4. Stergiopoulos V, Dewa C, Chau N, et al., *Collaborative street outreach: a novel approach to service delivery. Canadian Psychiatric Association Annual Meeting, Vancouver, BC.,* September 2008.
5. Goering P, Pyke J, and Bullock H, *Health, Mental Health, and Addiction Treatment Needs of People Using Emergency Shelters in Toronto. City of Toronto.* 2004.
6. Golden A, Currie WH, Griaves E, Latimer EJ. 1999. *Taking responsibility for homelessness: an action plan for Toronto. Report of the Mayor's Homelessness Action Taskforce.* Toronto: City of Toronto.
7. Nelson, G, Aubry, T, & Lafrance, A, (2007). *A Review of the Literature on the Effectiveness of Housing and Support, Assertive Community Treatment, and Intensive Case Management Interventions for Persons with Mental Illness Who Have Been Homeless.* American Journal of Orthopsychiatry, 77, 350-361.
8. Street Health. 2006. *Failing the Homeless: Barriers to the Ontario disability Support Program for Homeless People with Disabilities.* Toronto: Street Health. Available at www.streethealth.ca.
9. Wirshing D, Wirshing W. Informed consent: Assessment of comprehension. *Am J Psychiatry.* 1998; 155:1508-11.
10. Carpenter WT, Gold JM, Lahti AC, et al. Decisional capacity for informed consent in schizophrenia research. *Arch Gen Psychiatry.* 2000; 57:533-538.
11. Roberts LW, M.D.,, Warner TD, Ph.D., Brody JL, Ph.D. Perspectives of patients with Schizophrenia and psychiatrists regarding ethically important aspects of research participation. *Am. J. Psychiatry.* 2000; 157:67-74.
12. Barker S., Barron N., McFarland BH., et al., *A community ability scale foe chronically mentally ill consumers: Part II. Applications.* Community Mental Health Journal, 1994. 30: p. 459-472.
13. Durbin J et al, *The use of the Multnamah Community Ability Scale as a Program Evaluation Tool.* . Canadian Journal of Program Evaluation, 2004. 19(3): p. 135-157.
14. Guest G, Bunce A, and Johnson L. (2006). How many interviews are enough?: An experiment with data saturation and variability. *Field Methods*, 18, 59-82.

"At Home" Project
Research Demonstration Project in Mental Health & Homelessness: Main RCT Study

1. Background and Rationale

Homelessness is a significant social problem that affects thousands of people every night in cities across Canada. A study by the City of Toronto indicates that at least 5,052 individuals are absolutely homeless in Toronto on any given night [1].

The prevalence of mental health problems and addictions among homeless people is significantly higher than in the general population. The literature suggests that between one-fourth and one-third of the homeless have serious mental illnesses such a schizophrenia, major depressive disorder or bipolar affective disorder [2,3]. Research also suggests that homeless people in Toronto have complex, and often unmet, mental health needs [4,5].

Almost half of Toronto's 2.6 million residents are immigrants including a large proportion of refugee claimants in Canada. A 1999 report by the City of Toronto, identified immigrants and refugees as one of five groups that are particularly vulnerable and in need of targeted strategies [6]. However, the literature concerning the overlap between homelessness, mental illness and ethnicity, race or culture is scant, particularly in Canadian settings.

Overall, there have been only a small number of studies that have examined the effectiveness of housing and support interventions for people with mental illness who are homeless and all of these studies have been conducted in the United States. Research in this area reveals that programs that provide housing and support to people with severe mental illness are effective in reducing homelessness and hospitalizations and in producing other positive outcomes [7]. More data are needed that considers both the unique circumstances of ethno-racial groups and homeless people generally within the services and systems available in Canada.

2. Study Overview and Objectives

This goal of this study is to examine the effectiveness and cost effectiveness of providing housing and support services for homeless adults in Toronto who have serious mental health issues. Specifically, the study will examine the effectiveness of the Housing First model plus either (1) Assertive Community Treatment services or (2) Intensive Case Management services, compared with usual care. A particular focus of this study will be to examine the effectiveness of Housing First with Intensive Case Management services designed specifically for homeless adults from racialized ethno-racial groups. *For details about the models being examined see 3-I & 3-J below and Appendix L: Study Intervention and Usual Care Group Details.*

This research is part of a larger multi-site study sponsored by the Mental Health Commission of Canada. The Mental Health Commission of Canada is a federally funded, arms-length, non-profit organization with a mandate to focus national attention on mental health. Toronto is one of five cities (including Vancouver, Winnipeg, Montréal and Moncton) across Canada where research projects with a common core research design will take place. The Mental Health Commission of Canada's National Research Team manages the multi-site study and works

collaboratively with investigators at each site to implement and support the research activities. In Toronto, the research coordination lead is the Centre for Research on Inner City Health, St. Michael's Hospital. The service coordination lead is the City of Toronto's Shelter, Support and Housing Administration Division.

The overall goal of this multi-site national demonstration study is to provide policy relevant evidence and contribute to developing best practices that will be applicable to future efforts aimed at addressing mental health and homelessness across Canada.

This study has several guiding research questions:

- What are the effectiveness, cost/benefit, and cost-effectiveness of a Housing First approach plus Assertive Community Treatment supports in comparison to care as usual for single homeless adults with high need living in urban settings?
- What are the effectiveness, cost/benefit, and cost-effectiveness of a Housing First approach plus Intensive Case Management supports in comparison to usual care for homeless adults with moderate needs living in urban settings?
- What are the effectiveness, cost/benefit, and cost-effectiveness of a Housing First approach plus Ethno-Specific Intensive Case Management supports in comparison to usual care for homeless adults with moderate needs living in urban settings?
- What are the demographic, clinical, behavioral and social characteristics that moderate the effectiveness of the interventions?
- What are the critical ingredients of each model and modifications needed to effectively implement it in different cities and to effectively serve particular sub-populations?
- How do personal stories and lived experience of participants in the intervention and control conditions over time and how can those stories inform the practice, service delivery and policy implications of the study?

3. Study Design and Methods

A) Overview

This study is an impact evaluation that will be conducted using an experimental design in which multiple longitudinal mixed-methods measures will be collected on both the intervention and control groups. The intervention includes the provision of independent housing (based on the principles of 'Housing First') plus the development and implementation of three new services: a recovery-oriented Assertive Community Treatment (ACT) Team (based on the Pathways to Housing model in New York City), an Intensive Case Management (ICM) Team (based on the Streets to Homes model in Toronto); and an Ethno-Specific Intensive Case Management Team (developed in Toronto by a variety of stakeholders). The target population for this study is homeless adults with serious mental health issues.

B) RCT Design

The randomized controlled trial design involves a total of 560 participants stratified by level of service need and ethno-racial group membership.

Participants with high needs (n=200) will be randomized into one of two groups, the intervention (Rent Allowance plus Assertive Community Treatment) or Usual Care.

Participants with moderate needs (n=360) will be stratified by ethno-racial group membership and then randomized into one of two intervention options (Rent Allowance plus Intensive Case Management or Rent Allowance plus Ethno-Specific Intensive Case Management) or Usual Care. Study entry will be stratified to ensure that 240 of the 360 moderate needs participants are from ethno-racial groups. *For more details see Appendix U: Randomization Flow Chart.*

Moderate needs participants who are eligible for the ethno-specific intervention and who are randomized to intervention will be able to choose if they would like to receive Ethno-specific or regular ICM while there is still space in each intervention arm.

Participants in the Usual Care Group will be still be able to access the broad collection of services available in Toronto for homeless people with mental health issues. Participants in this group will be provided with information about available services in the community.

Data will be collected from participants in both groups approximately every 3 months for a period of 2 years from enrollment (see *Data Collection – Section K* below for details).

C) Target Population/Eligibility Criteria

The main criteria for inclusion in the project are as follows: (1) legal adult status (age 18) or older; (2) meets definition of 'absolute homelessness' or 'precariously housed'; (3) presence of any serious mental health disorder; (4) not currently receiving Assertive Community Treatment (ACT) or Intensive Case Management (ICM) services.

(2) Absolute homelessness is defined as: having no fixed place to stay for at least the past 7 nights and little likelihood of obtaining accommodation in the upcoming month; OR currently being discharged from an institution (e.g. jail, hospital) with no fixed address. No fixed place to stay is defined as: living rough in a public or private place not intended for human habitation (e.g. parks, vehicle, squats, and doorways), as well as those whose primary night-time residence is a supervised public or private emergency shelter.

(3) A candidate for inclusion demonstrates the presence of a serious mental illness when (i) the threshold for a diagnosable illness is met as established by the Modified MINI International Neuropsychiatric Interview or (ii) at least one psychotic behavior is observed/reported by the referring source in the past month or (iii) has documentation of a diagnosed mental health condition AND serious functional impairment is observed/reported by the referring source in the past month.

(4) Unmet ACT or ICM need will be determined using conversational interviewing to elicit what services the potential participant is receiving for help with their health or mental health, in getting a place to stay or to secure income. Participants will be asked for the names of programs and agencies where they receive services. This will be checked against a list of ACT and ICM programs in Toronto. Anyone receiving services from an agency or program on this list within the last 8 weeks will not be eligible for the study.

Please see Appendix C: Screening Tool for detailed definitions and eligibility cut-off scores.

Because women are often under represented among the absolutely homeless population, from which we are recruiting, they will be targeted to ensure they make up 25% of the total sample. Study entry will also be stratified to ensure that 320 of the 560 participants are from ethno-racial groups, 80 among the high needs group and 240 among the moderate needs group.

Eligibility for the ethno-racial intensive case management group will be based on self-reported responses to demographic racial identity questions obtained at baseline. *See Appendix D: Survey Instrument for details.*

Study participants will be classified as high or moderate need using criteria that correspond to Section 3 of the Ontario Standards for Assertive Community Treatment teams. Study participants who meet two or more of the criteria will be determined to be high need. All other study participants will be classified as moderate need. *For criteria details please see Appendix L: Study Intervention and Usual Care Group Details.*

D) Participant Recruitment

Concerted effort will be made to recruit participants from a range of living situations (e.g. shelter, street) and will aim to approximate the distribution of living situations documented among homeless people in Toronto in the 2006 Street Needs Assessment [1]. Recruitment to full sample is expected to take up to 18 months.

Recruitment to the study may come from several points of entry. Toronto's extensive network of organizations that serve homeless people will be the primary source of referrals. Any agency organization will be able to refer persons to the research coordinator for assessment of eligibility. This network includes but is not limited to: shelters, drop-in centres, street outreach programs, housing help offices, community health care centres, and emergency departments. Referrals by individuals will not be accepted. People who self-refer or individuals who are not affiliated with health or social service agencies but who would like to refer potential participants, will be directed by the Research Coordinator to agencies on the study's referral work group (see next paragraph) who will ensure that the potential study participant is connected to existing social services and, if appropriate, will then refer this person to the study.

To maximize the appropriateness of referrals to the project and to ensure that the entire range of potentially eligible individuals from across Toronto are referred to the project, the research team has convened a Referrals Work Group made up of representatives of likely referral sources (e.g. the Toronto Drop-in Network, the ER Alliance, the Shelter Network). Communication materials have been developed for targeted distribution to potential referral sources to ensure common understanding of the study's target population and eligibility criteria and appropriate referrals. Project staff will visit key referral agencies and organizations prior to study enrollment and during the early stages of the study to provide an overview of the study and discuss eligibility criteria and referral process.

A two-page referral form that can be faxed or emailed to the research coordinator will be distributed to potential referral sources. Referrals can also be provided to the coordinator over the phone. The referral form will request: the contact information of the referral source, the

individual's name, evidence of a mental health diagnosis if available, observed behaviours related to the study eligibility criteria, and some basic demographic information (necessary for study stratification) and information required to contact the individual. Referral sources will be directed to obtain verbal permission from potential participants before referring them to the study and will document that this has been obtained on the referral form.

Where participants give permission, referral sources will be notified if the person they referred has been enrolled in the study and to which arm. This is necessary in order to maximize continuity of service and to minimize participant vulnerability.

See Appendix G: Referral Information for Service Providers and Appendix H: Referral Information for Participants.

E) Screening/Eligibility Assessment

Initial contact with potential participants will be made in person primarily by health and social services providers who will have been provided with study information and referral materials including a script for approaching potential participants about the study. *Please see Appendix G for the referral script.*

Referrals will be made to a single individual (the study Research Coordinator) who will store any information collected for the purposes of recruitment in a locked filing cabinet. Access to this information will be restricted to this individual and the study intake coordinator.

An intake coordinator will receive referrals from the research coordinator and will work on a mobile basis to meet with individuals who have been identified as potential participants. Once referred to the study, potential subjects will be assessed in-person by the study intake coordinator who will establish eligibility and obtain consent. Assessments will take place at a safe and private location (possibly at the field office where the intervention service providers will be located) that is convenient to the potential subject.

Because the screening form collects personal health information, a verbal consent process will take place prior to the screening interview. Also, as part of the study we intend to analyze referral and screening data to understand more about who was not eligible or decided not to participate in the study. All referral data will be retained for a period of 10 years after the study has been closed and will subsequently be destroyed. Verbal permission will be obtained from participants prior to screening for permission to retain this information. *Please see Appendix O: Screening Consent Script.*

The verbal screening consent process will not replace the written consent process for the study itself. Because the study involves explaining multiple possible study assignments (i.e. high needs intervention, moderate needs intervention, ethno-specific intervention, and care as usual groups), we have decided to conduct the full study written consent after eligibility has been determined so as not to unnecessarily burden potential participants who may or may not be eligible for the study with an additional hypothetical situation (i.e. that they may or may not even be eligible to consider the full range of study participation assignments).

If a potential participant is screened but found to be ineligible for any reason (e.g. not 'absolutely' homeless, already receiving ACT or ICM services within the last 8 weeks), they will be able to be re-screened only once every 3 months while enrollment to the study is still open. Once someone has been enrolled to the study, they cannot be re-screened.

F) Consent Process

Written consent will be obtained from participants. Consent forms are crafted in an accessible, plain language format. The form will be read aloud to ensure that all participants, regardless of literacy level, will have been given the information required for them to be able to give informed consent.

Where potential subjects do not speak English, we will hire professional, certified translators who are competent in the required languages. In these cases, the study information and consent will be verbally translated. Translators will be asked to sign an oath of confidentiality and a declaration that the study was adequately explained and that consent was freely given.

The study may also involve participants who are illiterate or have low literacy levels. Consent forms have been crafted in accessible, plain language and will be read aloud and explained to all survey participants, regardless of literacy level. For participants who are unable to write, consent will be given orally and a witness will be present during the entire process to ensure that all aspects of the consent process are understood and that consent is given freely. A witness declaration and signature line has been included as part of the consent form.

Please see Section 4 below for additional protocols around consent and capacity.

G) Randomization Process

Participants will be randomized to either the intervention or control group after eligibility screening, consent process, and baseline interview (which will include classification of high v. moderate need).

The data vendor (described below in Section 5) contracted to provide data management and security for the national study will manage randomization for each site centrally. The study software will assign each participant's random selection, which will be generated using random number tables and stratified into 3 groups by level of need and ethno-racial group membership: (1) high need; (2) moderate need/non-ethno-racial group member; and (3) moderate need/ethno-racial group member. Tests of balance will be conducted to assess the success of the randomization procedures for both the high and moderate needs clients.

H) Sampling Strategies for Sub-Studies

Chart Review: At baseline, all participants will be asked to name their 5 most frequently used sources of health care in the past 3 years and for permission to obtain health records from these sources. Participants for chart review will be randomly selected from the larger sample stratified by gender, racialized ethno-specific group membership and treatment versus usual care assignment. Charts will be reviewed at all of the most frequently used sources of health care for the selected sub sample. Approximately 100 participants will be selected up to the entire sample, as budget and time allow.

Qualitative Interviews (Personal Narratives): Participants will be randomly selected from the larger sample stratified by gender, high need versus moderate need, ethno-racial group membership, proportional to the larger sample.

I) Usual Care Group

Homeless individuals with serious and persistent mental health issues may access a broad collection of health and social services available in Toronto that includes: inpatient and outpatient clinical service, peer support, crisis services, intensive case management, Assertive Community Treatment Teams (ACT), supportive housing, vocational programming, street outreach programs, drop-in services and shelters. Participants in the Usual Care Group will be still be able to access the broad collection of services available in Toronto for homeless people with mental health issues. Participants in this group will be provided with information about available services in the community. *Please see Appendix U: Resource Guide.*

It is recognized that some individuals in the usual care group may over time, through new or existing programs, access services that are similar to the experimental intervention. Health and social services use will be measured through the common protocol of health service use and included in the analyses of process and outcomes.

For additional information about Usual Care, please see Appendix L.

J) Intervention Group

The experimental intervention includes the development and implementation of three new services: a recovery-focused Assertive Community Treatment (ACT) team; and two Intensive Case Management (ICM) teams, one with a focus to serve homeless people from specific ethno-racial groups. Each of these teams will serve 100 participants and will be based at community-agencies.

In addition, participants in the experimental intervention will receive a rental allowance of $600 per month and support to obtain immediate access to housing. Participants will be allowed to choose their own housing after it meets minimum City of Toronto standards for acceptability, including property standards, affordability. The City of Toronto's Shelter, Support and Housing Administration Division will administer rent allowances and provide housing support to the ICM and ACT teams.

Participants who are randomized to the intervention group (N=300) will receive the rental allowance ($600 per month) plus support services (either an Assertive Community Treatment team or Intensive Case Management) until March 2013 and possibly beyond (if continued funding becomes available). Participants in the intervention group will be required to have weekly visits by a case manager or member of ACT team.

For additional details of the intervention, please see Appendix L.

K) Data Collection

Appendix B: Study Protocol

Multiple longitudinal mixed-methods measures will be collected on both the intervention and usual care groups.

Quantitative Survey Component

All study participants (in both the intervention and usual care groups) will be interviewed by phone or in-person every 3 months for a period of 2 years from baseline (for a total of 8 interviews). This includes: one 80-minute baseline survey interview followed by three 70-minute interviews (at 6 months, 12 months and 18 months), four 10-minute interviews (at 3 months, 9 months, 15 months and 21 months) and one 75-minute final interview. Participants will be asked questions about their housing situation, physical and mental health status, use of drugs or alcohol and overall quality of life. In addition, data on housing situation will be collected during monthly 5-minute check-ins by phone. *See Appendix D: Survey Instrument.*

Physiological measures

Select physiological measures will be collected from all participants at 5 points throughout the study: as part of the baseline interview and at every 6-month interview. The measures being collected are: systolic and diastolic blood pressure, height and weight (to calculate Body Mass Index), and waist and hip circumference.

Data Linkage

Links will be made to administrative data for all participants to obtain corroborative information on health care utilization in Ontario, prescription drug use, justice system contacts, shelter utilization in Toronto, disability and welfare benefits and official disability diagnoses. Participants will be asked for permission to the use of their personal identifiers (name, date of birth and Ontario Health card number) for this purpose. Linkages will be made with databases at the Institute for Clinical Evaluative Sciences (ICES), the Ministry of Community and Social Services, Correctional Service of Canada, the Ministry of Community Safety and Correctional Services, the Toronto Police Service, and the City of Toronto's Division of Shelter, Support and Housing Administration. The period of observation using these databases will include the 1-year prior to study enrolment and 5 years from enrollment. Individuals who do not have a health card in their possession at the time of the interview will be asked to provide us with contact information for recent health care providers, who can provide us with the individual's health card number. Individuals who decline permission for linkage to databases will still be eligible to participate in the study.

Chart Reviews

The health records of another subset of at least 100 participants will undergo review at baseline and at 24 months. Data will be collected retrospectively for a period of 3 years at study enrollment and at 24 months. Data collected from chart review will focus on diagnosis data, preventative health measures (screening and treatment), and select physiological measures where available. *See Appendix F: Chart Abstraction Guide.*

Qualitative Interviews (Personal Narratives)

A subset of 50 participants (20 from the intervention, 20 from the usual care group and 10 from the Ethno-Specific Intervention) will be randomly selected from the larger study group and be invited to participate in 2 in-depth qualitative interviews; the first within one month of entering

the study and the second at 18 months. Each interview will last approximately 90 minutes. Participants will be asked to discuss their experiences of homelessness and mental illness and to identify and discuss particularly memorable life events. *See Appendix E: Qualitative Interview Guide*

L) Follow-up Methods

Our goal is to retain 75% of participants over 2 years of follow-up, based on estimates and using methods that have proven to be effective in tracking and retaining homeless and marginalized study participants [8-14]. Efforts will be made to establish trust and rapport with participants at first contact and to explain the importance of their participation in follow-up interviews. All participants will be given a laminated card with a toll-free contact number for the study. Participants will be asked to call the contact number once every month. During the call, they will be asked to provide a brief update on their current living situation and location as well as updated contact information. A $10 cash incentive will be paid to the participant for each monthly contact.

To further minimize loss to follow-up between interviews, at the time of enrollment, participants will be asked to provide contact information not only for themselves but also for friends, relatives, service providers and case workers who are most likely to know the participant's whereabouts and who may be contacted in order to locate them. Participants will also be asked to give consent for the Ontario Ministry of Community and Social Services to provide us with their updated contact information or the contact information of their payee/trustee to the research team for participants receiving welfare or disability benefits. Contact persons will only be contacted when other means of finding study participants have been exhausted. *A script for contact with these individuals is attached in Appendix K.*

M) Honorarium

Participants will be paid an honorarium after each interview (lasting between 10 minutes and 1 1/2 hours) and for the monthly check-ins to recognize the value of their time, to encourage follow up and to help reduce barriers to participation. Honorarium amounts will be between $10 and $30 (depending on length of interview).

N) Data Analysis

Quantitative Data

Differences in the intervention and control groups will be examined by conducting t-tests and chi-square analyses at the individual level on a range of baseline outcomes and other demographic and risk factor characteristics. Power will be calculated for a range of effect sizes and intra-class correlations for the implemented longitudinal experimental design. Multi-level models will be implemented to estimate program effects and to study the trajectories of outcomes as a function of both between individual and time varying covariates. Robustness of results will be evaluation through propensity scoring methods used to match individuals in the treatment group with individuals from the control group on a number of baseline measures.

Because the Toronto sample alone is likely to be underpowered to address if the intervention has had differential impacts for each of the groups, we will work closely with the Mental Health Commission of Canada's national research team to implement a group-based approach for

analyzing developmental trajectories. This methodology applies finite mixture modeling to identify groups of individuals following similar trajectories on a variable measured (e.g. housing stability) repeatedly over time.

Analysis of data obtained from health care and social service databases in key areas will include: (1) identification and costing of all inputs (ii) identification and costing of outputs (iii) quantification of outcomes (iv) comparison of costs with outcomes (v) direct and indirect benefits of the intervention (based on program theory) (vi) potential short, immediate and long term benefits of the program.

Interim Analysis
Service utilization and participant survey data will be analyzed to examine differences in treatment and control group services delivery processes at the study's half way point. In the areas of safety monitoring, we will examine whether there have been any systematic negative consequences of the trial such as loss of services, poorer access to services than prior to enrollment into the study. We will have protocols in place to guide acute safety events (e.g., eviction from housing).

Outcome measures
The primary outcome measures of interest for measuring cost-benefit, effectiveness and cost-effectiveness for this study are:

- Housing Stability (Residential Time-Line Follow-Back Calendar)
- Quality of Life (EQ-5D and SF-12 Health Survey 1.0)
- Mental Health Status (Modified Colorado Symptom Index and Global Assessment of Individual Need - Substance Problem Scale)
- Community Functioning & Integration (Multnomah Community Ability Scale)
- Health care System Use and Costs (Standard health service utilization schedules, administrative data linkage).

Qualitative Data
Information from the personal stories from 50 individuals will be explored using qualitative longitudinal research methods with a focus on explaining the pathways by which the intervention affect the outcomes. Qualitative data analysis will follow a systematic, comparative grounded theory method of analysis. Line-by-line coding of transcribed text from in-depth interviews will identify key concepts and will be facilitated by the constant comparative method involving the comparison of themes and concepts as the analysis progresses.

Chart Review
Analysis will focus on comparisons between intervention and usual care groups on rates of adherence with preventive health service guildlines and measured physiological parameters. Analysis will also examine the accuracy of self-reported health status compared with chart documented diagnosis.

D) Instrument Pre-Test

Quantitative survey instruments were pre-tested (see REB #09-161) using conventional and cognitive interview techniques to: (i) identify concerns related to face validity, timing, flow, and general acceptability of the proposed survey tools; and (ii) explore issues of meaning, comprehension, recall, and disclosure. The results of pre-testing will be used to make modifications to the survey tools that will improve the content and administration process. In the case of highly standardized instruments, where item changes were not possible, the information collected will be used at the analysis stage to inform the interpretation of findings for specific items and scales.

P) Sample Size Justification

RCT Study:

It has been estimated that a clinically meaningful and realistically achievable effect size (the between group difference in the mean value of continuous normally distributed outcome variables), is approximately half of the within group standard deviation. This also corresponds to what Cohen refers to as a moderate effect size (between 0.3 and 0.5) [15]. While there are potentially multiple outcomes for the proposed study, the MCAS provides an excellent outcome to use in a sample size calculation as it captures multiple dimensions of well-being and functioning of participants and we have data on standard deviations on many samples [16,17]. Such studies have reported standard deviations of approximately 9.0.

We performed separate sample size calculations to address each of the following research questions: (1) Among high needs homeless adults: to compare ACT with usual care; (2) Among moderate needs homeless adults: to compare ICM with usual care; (3) Among moderate needs homeless adults from ethno-racial groups: to compare ES-ICM with usual care. For question (2) we will conduct sample size calculations for treatment comparisons among ES-eligible and among Not ES-eligible, as well as treatment comparisons among the pooled the ES- and Not ES-eligible groups.

Among the moderate needs, ES-eligible stratum, our design allows for a participant randomized to treatment to be able to choose between ICM or ES-ICM up to a maximum to 40 ICM participants and 100 ES-ICM participants (see *Appendix V: Randomization flow chart*). These limits are determined by the ability of the service providers to offer the experimental treatments to at most 100 clients during the study period.

The following table shows the minimum MCAS mean difference at the end of the study for the proposed sample sizes, assuming within group standard deviation=9, power=90%, alpha=5%, 2-sided two-sample t-test to compare means.

COMPARISON	N_T	N_C	MEAN DIFFERENCE
High Needs: ACT vs usual care	100	100	4.1
Moderate Needs: ICM vs usual care			
Not ES-Eligible	60	60	5.4
ES-eligible	40	100	5.5
All	100	160	3.7
Moderate Needs- ES-ICM vs usual care	100	100	4.1

N_t = sample size of experimental arm; N_c = sample size of usual care arm

Thus we seek overall differences between our experimental and control arms of between 3.7 and 5.5 points on the MCAS, representing moderate to large effect sizes of 0.4 to 0.6. Similar or larger differences have been observed in comparing high risk to low risk populations for example [18].

Finally, we anticipate that there will be uniform attrition across all groups and estimate it to be approximately 25%. Our power will no longer be 90% to detect each of the differences in the table above at the 5% significance level: we estimate it to be 79.1%, 80.3%, 80.0%, 79.5% to detect a mean differences of 4.1 with 75 in each group, 5.4 with 45 in each group, 5.5 with group sizes of 30 and 75, and 3.7 with sizes of 75 and 120. Loss of power when comparing MCAS means at the end of the study with t-tests in presence of attrition will be remedied by using other statistical techniques, such as mixed modeling, that consider all repeated measures, rather than summary measures, in this longitudinal study.

Qualitative Component (Personal Narratives):
We expect that 50 interviews will provide a large enough sample to achieve saturation of codes and themes [18], as well as a minimum level of statistical power.

Chart Review
We plan to review as many charts as possible, contingent on budgetary restrictions. We currently have 1,220 hours of staff time allocated for chart review and expect to be able to review at least 100 charts.

4. Ethical Issues

Study Design
There have been no experimental studies of this housing and support model in Canadian settings. In order to influence policy we need a strong design to find out whether this intervention works, at what cost and for whom in our local contexts. In order to determine which models work best for whom, we need to compare people who are receiving housing with supports with people who are not receiving these services. Individuals in the 'care as usual' group will be offered referrals to existing services and will not be placed at any greater risk of harm than they face in their current circumstances.

Risk to Participants
There are no additional risks associated with the study as compared to usual standard of care. Subjects may experience some discomfort as a result of being asked about personal health experiences, which may have been painful. A referral/resource list will also be part of each interview kit so that interviewers will be able to make referrals to appropriate community services if participants require support during or after the interview process. A safety/risk protocol has been established by the national research team which will give Research Assistants a back-up clinician to contact based on a specific operational decision around responses on risk items, a referral card for a contact clinician, and a number for crisis services. *Please see Appendix S:*

Safety Protocol.

Individuals who are randomized into the usual care group may feel some disappointment over not receiving the intervention benefits (housing and supports). This will be minimized by providing all members of the care as usual group with information about existing available services in the community. Where permission is given, the Research Coordinator will notify the referral source that the participant was randomized to the usual care group so that this health or social service provider is aware that the individual will still need support to find housing and supports outside of the study.

Privacy and Confidentiality
This study proposes to use multiple and varied data sources (including charts, Ministry of Health data, and primary data collection) for each participant. This comprehensiveness is critical to the scientific objectives of the study, yet may increase invasion of individual participant privacy. This privacy concern will be clearly communicated to potential participants, as will the measures for protection of security and confidentiality, prior to consent. Any potential vulnerability of participants will be minimized since study findings will be kept confidential, de-identified soon after collection, and because participants are not required to answer any questions that they do not want to and they may stop the interview at any time.

Data protection measures are outlined in Section 5.

Consent and Capacity
Participants will be enrolled in the study only if they are able to give informed written consent. Assent will not be accepted. Having a mental illness does not automatically make an individual incapable to consent to treatment or to participation in research studies [19]. Several studies have shown better decisional capacity among individuals with severe mental illness, even while actively symptomatic, then previously believed [20-22]. Nevertheless, it is acknowledge that some potential participants may be compromised in their ability to provide consent or that this may fluctuate over time.

In an effort to alleviate any lack of understanding of study involvement, we propose to take some extra educational and communicative steps that have been recommended in the literature [23,24]:

- use a simple, clear and repeated informed consent process
- document observations about understanding of participation at the time of consent

If a potential participant is temporarily unable to provide consent at the time of their initial referral (e.g. due to intoxication, acute exacerbated psychosis, acute medical illness), the study intake coordinator will ask the participant to contact the research coordinator at a later date.

Undue Inducement
While it may seem that the possibility of receiving a rental allowance and supports would be a difficult offer for someone who is homeless and experiencing serious mental health issues to refuse, it should be noted that a City of Toronto survey found that about 1 in 10 homeless people do not want permanent housing [1]. Of those who did not want housing, 20% said they preferred being homeless, 14% said they did not want help finding housing and 20% said they did not want to live in Toronto or were just traveling through [1]. (Note: our study is only able to offer people

housing in the City of Toronto). It is also likely that some homeless people will not want the particular type of housing being offered through our study – i.e. primarily independent, self-contained units where the support services are not directly attached to the housing. Some individuals will prefer a congregate living situation with on-site supports. Many may also prefer an alternative and less intensive form of mental health support services. As explained in the study consent process, participants must be willing to have contact with a study service provider at least once per week. This will not appeal to everyone who is eligible for the study as many potential participants will prefer less frequent or less structured models of case management support.

Withdrawal from Intervention
We do not envision any premature withdrawal of subjects from the study for safety concerns. Participation in this study is completely voluntary and participants may withdraw from the study at any time. Participants in the intervention group will not lose their housing or support services should they decide to withdraw from the research activities at any point. Given the pragmatic nature and the population of this study, we feel it is ethically perilous to consider withdrawing the rent allowance and supports if a participant refuses to continue with the research activities of the project, especially since a return to a non-research situation would mostly likely be a return to homelessness. Moreover, if participants are told that their housing and supports will be removed if they do not comply with the research activities then this may be viewed as undue influence on the part of the research team.

It should also be noted that we do not anticipate that many participants in the intervention group will wish to withdraw from the research activities exclusively. The research activities themselves (monthly 5-minute check-ins by phone, one 80-minute survey interview at baseline followed by periodic interviews over two years ranging from 10 to 80 minutes) do not represent a significant additional burden for participants who are housed and receiving support services. Participants in the intervention group will have likely formed positive relationships with the study service providers and we anticipate that they will be willing to participate in periodic interviews for which they will receive an honorarium. With direction from the participant's ACT team or ICM case manager, research staff will periodically approach these individual's to see if they might be willing to re-join the study. Permission to maintain contact with participants who have withdrawn from research activities during the 2-year participant enrollment period will be discussed as part of the consent process.

Sustainability & Transition
The potential for evictions of participants in the intervention group at the end of the project (March 31, 2013) is a risk and concern. The project team has a responsibility to ensure that participants do no become homeless and without case management supports again at completion of the research project. Everyone in the intervention group will be helped to either stay where they are or move to other long term housing. Participants will not be transitioned back to shelters or the street, or to potentially unstable pilot projects. In the absence of funding from sources other than the Mental Health Commission of Canada beyond the duration of the study, we will instruct service providers in year 3 to access rent-geared-to-income housing and to ensure access to alternate appropriate support services.

Everyone in the intervention group will be put on the social housing wait list (average wait is approximately 1 year for a bachelor apartment) and study service providers will partner with other support agencies in the community where participants can then be referred in the absence of extended funding after study completion. Participants will be updated about the sustainability of the project on an ongoing basis through their service provider and service providers will include participants as decision makers in their individual transition plans.

Planning for post-study system integration and sustainability has been part of this project from the outset. Program sustainability objectives include identifying appropriate strategies for scaling up successful elements of the demonstration project to be inclusive of all eligible clients in the Greater Toronto Area. Planning for and championing program transition and sustainability has begun with the establishment of a Local Advisory Committee for the project. This committee is made up of experts with extensive knowledge of health and social policy frameworks in Ontario, including service providers and people with lived experience of mental illness and homelessness. This committee is responsible for identifying feasible options for integrating successful demonstration project elements into the core funding portfolios of the relevant administrative agencies and championing program adoption with relevant stakeholders and agencies.

Ideally, if funding is extended, we would offer the effective services to the control group participants as well, but this is not necessarily feasible. Given limited resources, our first obligations are to those in the intervention group with whom we have entered into a fiduciary relationship as a service and support provider.

Safety of Research Personnel
Research assistants will be hired based on their experience with research and working with individuals who have mental illness and addiction. All research staff will undergo training in how to conduct research interviews with individuals who are homeless and have mental illness. Specific training will be provided around non-violent crisis management, and personal safety. Although the likelihood of violence against staff is low, safety precautions such as having two researchers present in a participant's home if she/he has a violent history will be taken. *Please see Appendix S: Safety Protocol.*

5. Community Stakeholder Involvement

This research project has been developed collaboratively with the study service providers and the Toronto community. Broad and meaningful community participation has been sought to shape the content of this protocol, and we have aimed to conduct our work in line with core principles of equitable decision-making, particularly transparency and defensibility.

Since the beginning of the study development process, people with lived experience of mental health issues and/or homelessness have participated in planning meetings concerning governance, research approaches, and strategic implementation. Structures and guiding principles have since been established to ensure that people with lived experience are able to contribute to and communicate with the project and to ensure that their experience and perspective informs (a) appropriate and ethical implementation of services; (b) appropriate and

ethical research practices; (c) governance of this project; (d) development of local research questions and local methods for gathering and interpreting data; (e) approaches for informing local and national stakeholder communities (including consumers, policy-makers, and the media) about the project; (f) approaches for disseminating the policy implications of the study and information about homelessness and mental health; (g) approaches for sustaining the interventions after the research project is over. A caucus of approximately 20 people with lived experience of mental health and/or homelessness meets monthly to discuss the study and provide direct input and feedback to this stakeholder group through caucus membership on the study work groups (described below).

A project 'Site Operations Team' meets every other week and is responsible for the implementation and day-to-day operations of the project. The Site Operations Team includes project leads from CRICH and the City of Toronto's Shelter, Support and Housing Administration Division, the community-based ACT and ICM service providers, two people with lived experience of mental health issues and/or homelessness and a representative from the Mental Health Commission of Canada. In addition, a series of Site Operations Team work groups focused on specific aspects of the study (Research, Housing, Referrals, Services, Ethno-Specific Services) meet on a monthly basis to inform and guide the project. These work groups include additional community stakeholders including people with lived experience and external service providers. As mentioned in Section 5 (*Sustainability & Transition*), a Local Advisory Committee also allows for community input into the study.

6. Data Management & Security

The Mental Health Commission of Canada, through a competitive process, selected a vendor to provide data collection software and information systems equipment and services for the study. The chosen vendor is HealthDiary, a Canadian-owned company based in Toronto. HealthDiary has a strong track record in providing similar systems for other multi-site health research studies across Canada which have been submitted to and received ethics approvals from relevant academic ethics review boards. These studies include: the Canadian Healthy Infant Longitudinal Development Study (CHILD), the Christmas Epidemic of Exacerbations of COPD.

Specific details of HealthDiary's compliance with industry standards are provided in Appendix P.

System Design

The system is designed as a secure web-based application that will provide the data collection and storage services required for the project. It includes features for secure access control, secure data management, data encryption, network security and privacy. The system will be assessed by an information systems security expert external to the company at the design stage, and security testing will be completed and documented by the core development team (which includes Health Diary staff, information systems staff of the Mental Health Commission of Canada and National Research team staff) prior to initiating data collection.

The research database application will run on a cluster of dedicated servers that provide database, application and web services only for HealthDiary's clients. These servers are located in a Tier 3+

commercial data centre in Toronto provided by Bell Canada. The data centre environment provides high levels of physical and network security, redundant Internet connections, redundant power supply, fire suppression systems and heating and ventilation systems. This environment provides the robust, resilient and secure environment required for hosting the application.

Physical access to the hardware that comprises the system will be restricted to authorised personnel only. The servers reside behind a dedicated hardware firewall and all network access to the application and servers is protected by security policies that include role based access controls, strong password and authentication, anti virus / anti-malware filtering and encryption of all traffic using Secure Sockets Layer (SSL). All hardware and operating systems are monitored and maintained on a regular basis for security and availability purposes.

All data within the application will be encrypted and protected with strong passwords and authentication. All identifiers in the data will be removed once the data has been collected and verified. The master list linking data with identifiers will be segregated and stored secured from the data. The application data is backed up daily to a secure storage location in the data centre. Physical access to these backups is highly restricted and the backups are password protected. Data backups are also taken offsite from the data centre by a secure transport service to a secure storage location provided by Iron Mountain. Physical access to the data backups at Iron Mountain is restricted to authorised personnel only.

Data Collection

In the field, quantitative data will be collected from participants by trained Research Assistants via interviews through a Computerized Assisted Personal Interviewing (CAPI) system. To access the HealthDiary application only an Internet browser is required and the application does not store any study data on the laptop's local hard drive. Microsoft's ReadyState technology will be used to prevent users from installing software, restricting access to features such as Control Panel, or storing data on local hard drives. A computer reboot will automatically restore the computer to the original configuration. The laptops will connect via the internet using Broadband Wireless technology and communication between the laptop computers and servers will be encrypted using SSL technology.

If for any specific interview in the field a Research Assistant is unable to connect to the central database (the frequency of this circumstance is expected to be rare), the information will be collected on hard copy, carried directly back to the research office by the Research Assistant, entered and then shredded. Research Assistants will be trained on security procedures around paper records, i.e. the records will be kept on their person at all times and returned to the research office the same day. Storage at home or in a car will not be permitted.

Personal information, for the purposes of scheduling and attending interview appointments with participants, may be printed from the central database via office-based desktop computers and will be similarly protected while in the field, and filed in a locked, limited access file cabinet in the research office, which will also have limited keyed access with no public access. Interviews that are conducted in the field office will be conducted in a separate space.

Connection under strict access controls will also be permitted via existing desktop computers, but only by authorized staff using specified desktop computers in allowable research sites and governed by specific security and confidentiality procedures for the study and the sites.

Qualitative data will be collected using digital recorders, transcribed and uploaded to a separate directory of the database. Study identifiers will be used to allow for connection of quantitative variables (e.g. demographics) to the qualitative data at the time of analysis; this will be done outside the central database, in other software, by the lead researchers for the qualitative components of the national study (Dr. Geoff Nelson and Dr. Myra Piat).

Access
Access to the application and data will be limited to authorised users only, using a multi-level, permissions protocol that will specify roles and types of data access permitted, using the need to know principle. A small group of national research team members (Dr. Jayne Barker, Dr. Paula Goering, Dr. Carol Adair and Dr. David Streiner) will have access to the full dataset from all sites. Because the profile data (i.e. identifying information) is kept separate in the database, access can be restricted centrally and within sites to those with a specific need for it only. When the data are exported for analysis, only study numbers will be present in the file. Co-Principal Investigators, Research Coordinators and Research Assistants in Toronto with specific roles such as participant follow-up or data review and management will have access to data for such specified purposes; but not to any data collected by other sites. Toronto Co-Investigators will have access to data for the purposes of data analysis but will not have access to personal identifiers.

All users will have unique usernames and will be required to use strong passwords. The system has protection against brute force attacks on the authentication pages. In addition, the system will automatically record the details of system security in the form of user access logs, data access logs and system logs. User access logs will be reviewed at least weekly and the other logs will be preserved for the duration of the study for special audits if needed.

All investigators and research personnel that have access privileges will receive specific orientation to the system procedures and sign a Confidentiality and Security declaration outlining their duties and responsibilities in accessing the system.

Data from other sources (e.g. provincial or regional administrative databases) at each site will not be linked with participants' records in the central database; the subset of data for each site will be output from the central database in SAS format and linked in local databases for analysis, in compliance with the ethics and organizational regulations governing each site.

7. Knowledge Translation

An integrated knowledge translation approach oriented toward sustainability planning is an important component of the project. In collaboration with the study's Local Advisory Committee (made up of provincial government representatives, service providers and individuals who have experienced homelessness and mental illness) a communications plan

will be developed and key partners and stakeholders will be identified to ensure the engagement of key target audiences in knowledge production and dissemination activities to ensure adoption of evidence and use of knowledge to inform policies.

References

1. City of Toronto, Shelter, Support and Housing Administration Division. 2006. *2006 Street Needs Assessment: Results and key findings.* Toronto: City of Toronto. Available from: www.toronto.ca/housing/pdf/streetneedsassesment.htm.
2. Fischer P and Breakey W, *The epidemiology of alcohol, drug and mental disorders among homeless persons.* American Psychologist, 1991. 46: p. 1115-1128.
3. Koegel P, Burnam M, and Farr R, *The prevalence of specific psychiatric disorders among homeless individuals in the inner city of Los Angeles.* Archives of General Psychiatry, 1988. 45: p. 1085-1092.
4. Stergiopoulos V, Dewa C, Chau N, et al., *Collaborative street outreach: a novel approach to service delivery.. Canadian Psychiatric Association Annual Meeting, Vancouver, BC.,* September 2008.
5. Goering P, Pyke J, and Bullock H, *Health, Mental Health, and Addiction Treatment Needs of People Using Emergency Shelters in Toronto. City of Toronto*. 2004.
6. Golden A, Currie WH, Griaves E, Latimer EJ. 1999. *Taking responsibility for homelessness: an action plan for Toronto. Report of the Mayor's Homelessness Action Taskforce*. Toronto: City of Toronto.
7. Nelson, G, Aubry, T, & Lafrance, A, (2007). *A Review of the Literature on the Effectiveness of Housing and Support, Assertive Community Treatment, and Intensive Case Management Interventions for Persons with Mental Illness Who Have Been Homeless*. American Journal of Orthopsychiatry, 77, 350-361.
8. Cohen EH, Mowbray CT, Bybee D, Yeich S, Ribisl K, Freddolino PP. Tracking and follow-up methods for research on homelessness. Evaluation Review. 1993; 17:331-52.
9. Cohen, J. (1988). Statistical power analysis for the behavioral sciences (2nd ed.). New Jersey: Lawrence Erlbaum.
10. Hough, RL, Tarke H, Renker V, Sheilds P, Glatstein J. Recruitment and retention of homeless mentally ill participants in research. Journal of Consulting and Clinical Psychology. 1996; 64:881-91.
11. McKenzie, M., Peterson Tulsky, J., Long, H. L., & Chesney, M. (1999). Tracking and follow-up of marginalized populations: a review. Journal of Health Care for the Poor and Underserved, 10, 409-429.
12. Pollio DE, Thompson SJ, North CS. Agency-based tracking of difficult-to-follow populations: Runaway and homeless youth programs in St. Louis, Missouri. Community Mental Health Journal. 2000; 36:247-58.
13. Ribisl KM, Walton MA, Mowbray CT, Luke DA, Davidson WS II, Bootsmiller B. Minimizing participant attrition in panel studies through the use of effective retention and tracking strategies: Review and recommendations. Evaluation and Program Planning. 1996; 19: 1-25.
14. Wright JT, Allen TL, Devine JA. Tracking non-traditional populations in longitudinal studies. Evaluation and Program Planning. 1995; 18:267-77.
15. Cohen J, *Statistical power analysis for the behavioral sciences.* Second ed. 1998, New Jersey: Lawrence Erlbaum.
16. Pollack ME, Billick SB. Competency to consent to treatment. *Psychiatric Quarterly.* 1999; 70:303-311.

17. Appelbaum PS, Grisso T, Frank E, O'Donnell S, Kupfer DJ. Competence of depressed patients for consent to research. *Am J Psychiatry*. 1999; 156:1380-1384.
18. Grisso T, Appelbaum PS, Hill-Fotouhi C. The MacCAT-T: A clinical tool to assess patients' capacities to make treatment decisions. *Psychiatric Services*. 1997; 48:1415-1419.
19. Wirshing D, Wirshing W. Informed consent: Assessment of comprehension. *Am J Psychiatry*. 1998; 155:1508-11.
20. Carpenter WT, Gold JM, Lahti AC, et al. Decisional capacity for informed consent in schizophrenia research. *Arch Gen Psychiatry*. 2000; 57:533-538.
21. Roberts LW, M.D., Warner TD, Ph.D., Brody JL, Ph.D. Perspectives of patients with Schizophrenia and psychiatrists regarding ethically important aspects of research participation. *Am. J. Psychiatry*. 2000; 157:67-74.
22. Barker S., Barron N., McFarland BH., et al., *A community ability scale foe chronically mentally ill consumers: Part II. Applications.* Community Mental Health Journal, 1994. 30: p. 459-472.
23. Durbin J et al, *The use of the Multnamah Community Ability Scale as a Program Evaluation Tool.* . Canadian Journal of Program Evaluation, 2004. 19(3): p. 135-157.
24. Guest G, Bunce A, and Johnson L. (2006). How many interviews are enough?: An experiment with data saturation and variability. *Field Methods*, 18, 59-82.

BASELINE INTERVIEW
PERSONAL NARRATIVE INTERVIEW GUIDE

Introduction

[Complete informed consent]

This interview is an opportunity for you to tell the story about your experiences living on the streets or in a shelter. We're interested in learning about what life has been like before and after you started living on the streets or in a shelter. Take the time you need. For most people it takes about 90 minutes, but how much time we take to do the interview is up to you. We can take a break if you wish. Just as a reminder, please be aware that your participation in the study is completely voluntary. You can decide not to participate, to withdraw your participation at any time, and to skip any questions that you do not wish to answer. Also, your decision to participate or not participate will not affect the services or support your receive. You may find some of these questions sensitive or disturbing. We will only proceed with the interview today if you feel comfortable doing so. We will hold everything that you say in confidence. Please note that your name will not be associated in any way with your responses. You will receive a written summary of the findings when the research is completed. Do you have any questions before we get started? I'm going to start the recorder now – is that still okay with you?

Part I: Story of Living on the Streets or in a Shelter

I'm interested in learning about your experiences in housing. Now I'm going to ask you some questions about that.

- **Probe about Life Before Living on the Streets or in a Shelter:**

- What was your life was like before you started living on the streets or in a shelter?
  - Are there issues/experiences in your childhood that were related to you living on the streets or in a shelter? (probe for when left parents' home)
- When you were housed, what do you feel helped you to stay housed?

- **Probe about Life on the Streets or in a Shelter:**

- How has your life changed since you started living on the streets or in a shelter? (probe re: how you felt about yourself, relationships, family, friends, health, involvement in the community, poverty, stigma, addictions)
- Describe some of the places you've lived and what it's been like on the streets or in a shelter? (probe safety, quality, support)
- What has been hardest for you since you've been living on the streets or in a shelter? (probe re: how you felt about yourself, relationships, family, friends, work, health, involvement in the community, poverty, stigma, addictions)

- Please describe to me what your average day is like. (probe re: places visited, people met with, the nature of the encounters with people)
- Please tell me about what keeps you going. What do you enjoy doing?
- Have you had contact with your family, while while living on the streets or in a shelter? How would you describe your relationships with your family?
- How would you describe your relationships with friends (both people on the streets and people who are housed) while while living on the streets or in a shelter?
- What services, supports, or community organizations have you used while living on the streets or in a shelter? What are they like? What types of services/supports/community organizations do you find most helpful (e.g., services, family, friends, church)? What types of services/supports have been least helpful?
- How would you describe your involvement in the community while living on the streets or in a shelter?
- Have you ever felt that you were treated unfairly by landlords, superintendents or neighbours? Please tell me about this.
- What has been your experience of stigma and discrimination in relation to services and housing?
- What do you see as the ideal housing situation for you (probe for individual vs. shared living situations; landlord relationships; location; safety issues)?
- **Ask this question only of those persons in one of the housing interventions** – What do you think of the "At Home" intervention project in which you will be involved?

- **Probe First Experiences of Mental Health Problems:**

- Please tell me about when you first remember struggling with thoughts and emotions that affected your mental health?
    - What was your life like at that time (probes: how you felt about yourself, relationships, family, friends, physical health, involvement in the community, poverty, stigma, addictions)?
- What were your first experiences like with receiving help for mental health issues?
- What has your experience with mental health services been like since that first time? Did these services meet your needs?
- Have you ever felt that you were treated unfairly when using these services? Please tell me about that.
- Is there anything that you would change or improve about the mental health services that you have used?

Part II: High-, Low-, and Turning Point

I'm now going to ask you to highlight a high-point, a low-point and a turning-point from your life. Let's start with the high point[1].

Note to Interviewers: [Make sure that the participant addresses all of the following questions, especially ones about impact and what the experience says about the person. Do not interrupt

[1] If the participant has already recounted a high-, low-, and/or turning point-story earlier, there is no need to ask about this again here at the end of the interview.

the description of the event. Rather ask for extra detail, if necessary, after the participant has finished initial description of the event.]

High Point Story

I would like you to reflect on a high point in your life, what you might think of as the best moment in your life. It could be a moment or time in your life where you experienced very positive feelings, such as joy, excitement, happiness, or inner peace. Does an event or time like this come to mind? Describe it for me in detail. Make sure to tell me what led up to the scene, so that I can understand it in context. What happened in the scene? Where and when did it happen? Who was involved? What were you thinking and feeling in the event? Why is it an important event? What impact has this event had on who you are today?

Low Point Story

Think back over your entire life and try to remember a specific experience or event where you felt really low: it could involve emotions such as deep sadness, fear, strong anxiety, terror, despair, guilt, or shame. You might think of this as the worst moment in your life. Please describe this scene for me in detail. Again, tell me what led up to the scene, so that I can understand it in context. Where and when did it happen? Who was involved? What happened? What were you thinking and feeling? Why is it an important event? What impact has this event had on who you are today?

Turning Point Story

In looking back on your life, are there any big "turning points" that come to mind? This could be times when you experienced an important change in your life.
IF YES: Please choose one key turning point scene and describe it in detail.
IF NO: Describe a particular time in your life that comes closer than any other to qualifying as a turning point – a scene where you changed in some way.
Again, tell me what led up to the scene. What happened? Where and when did it happen? Who was involved? What were you thinking and feeling? Why is it an important event? What impact has this event had on who you are today?

What does "home" mean to you?
What are your hopes for the future?

Ending the Interview

- How are you feeling right now?
- Is there anything that we have not covered that you think is important for me to know about how being homeless has affected your life?
- What did you think of the interview?
- Did you feel comfortable doing this interview?
- Is there anything we can do to improve the interview?
- Do you have any questions of me?

Thank you very much for participating in this interview. I appreciate your willingness to share your story with me – this is an important part of the project.

The following data will be abstracted by way of chart review:

Physical Health Diagnoses: these diagnoses will be searched for throughout all of the charts from health care providers identified by participants and the date of diagnoses will be recorded.
- Asthma
- Fibromyalgia
- Back problems
- High blood pressure
- Migraine headaches
- Chronic bronchitis, Emphysema or Chronic obstructive pulmonary disease (COPD)
- Diabetes
- Heart disease
- Effects of a stroke
- Urinary incontinence
- Bowel disorder (such as Crohn's disease, IBS, ulcerative colitis, bowel incontinence)
- Alzheimer's disease or any other dementia
- Kidney or bladder trouble
- Arthritis or rheumatism (excluding fibromyalgia)
- Epilepsy or seizures
- Glaucoma
- Thyroid condition
- HIV positive test or AIDS
- Hepatitis B
- Hepatitis C
- Cirrhosis of the liver

Mental Health Diagnoses: these diagnoses will be searched for throughout all of the charts from health care providers identified by participants and the date of diagnoses will be recorded. However, only psychiatric consultation notes will be recorded for the purpose of abstracting psychiatric diagnoses. Multiple diagnoses will be abstracted where required.
- Mental retardation
- Learning disorder
- Pervasive developmental disorders
- Attention-deficit and disruptive behaviour disorders
- Tourette's disorder
- Dementia
- Alcohol dependence

- Alcohol abuse
- Amphetamine dependence
- Amphetamine abuse
- Cannabis dependence
- Cannabis abuse
- Cocaine dependence
- Cocaine abuse
- Hallucinogen dependence
- Hallucinogen abuse
- Inhalant dependence
- Inhalant abuse
- Opioid dependence
- Opioid abuse
- Phencyclidine dependence
- Phencyclidine abuse
- Sedative, hypnotic, or anxiolytic dependence
- Sedative, hypnotic, or anxiolytic abuse
- Polysubstance dependence
- Schizophrenia
- Schizophreniform disorder
- Schizoaffective disorder
- Delusional disorder
- Brief psychotic disorder
- Substance-induced psychotic disorder
- Psychotic disorder NOS
- Major depressive disorder
- Dysthymic disorder NOS
- Bipolar I disorder
- Bipolar II disorder
- Cyclothymic disorder
- Bipolar disorder NOS
- Mood disorder NOS
- Panic disorder without agoraphobia
- Agoraphobia without history of panic disorder
- Specific phobia
- Social phobia
- Obsessive-compulsive disorder

- Post-traumatic stress disorder
- Acute stress disorder
- Generalized anxiety disorder
- Substance-induced anxiety disorder
- Anxiety disorder NOS
- Conversion disorder
- Pain disorder
- Factitious disorder
- Dissociative amnesia
- Dissociative identity disorder
- Depersonalization disorder
- Anorexia nervosa
- Bulimia nervosa
- Intermittent explosive disorder
- Adjustment disorder
- Schizotypal personality disorder
- Antisocial personality disorder
- Borderline personality disorder
- Narcissistic personality disorder

Preventative Health Services: these services will be abstracted from all charts from health care providers identified by the participant. They will be assessed for whether the service was performed at all and also the most recent date of service delivery recorded in the chart.
- Blood pressure measurement by a health care provider
- Influenza immunization 1x/year
- Screen for obesity based on BMI
- Intensive counseling and behavioral interventions to promote sustained weight loss
- Screening for cholesterol and elevated blood lipids(total cholesterol, HDL, LDL, triglycerides, HDL/TC ratio)
- Screening for cervical cancer using a papanicolaou smear
- Screening for diabetes mellitus (fasting blood glucose, HbA1c, 2-hour glucose-tolerance test)
- Screening for breast cancer using mammography

Physiological measures: these values will be recorded if available in any of the charts from health care providers identified by participants.
- Serum lipid profile (total cholesterol, HDL, LDL, triglycerides, HDL/TC ratio)
- Fasting blood glucose, HbA1c, 2-hour glucose-tolerance test

"AT HOME" Research Demonstration Project in Mental Health & Homelessness Information for Referral Sources

---

Funded by: The Mental Health Commission of Canada (MHCC)

Principal Investigators: Dr. Stephen Hwang, Dr. Vicky Stergiopoulos, Dr. Pat O'Campo; Centre for Research on Inner City Health (CRICH), St. Michael's Hospital.
Service Coordinator: Shelter, Support and Housing Administration, City of Toronto.

Research Coordinator: Kate Mason, masonk@smh.toronto.on.ca, (416) 864-6060 ext 6513.

Purpose: To examine the effectiveness and cost effectiveness of housing and supports for homeless people with serious mental health issues. The goal of this study is to create evidence for policy makers about what services and supports work best for this group.

Method: This study will evaluate 3 different models of housing and supports:
1. Housing with Assertive Community Treatment (ACT) support for individuals with high needs
2. Housing with Intensive Case Management (ICM) support for individuals with moderate needs
3. Housing with Ethno-racial Intensive Case Management support designed specifically for people from racialized groups with moderate needs

The study design is a Randomized Controlled Trial. 560 people will be enrolled in the study: 300 will receive housing and supports and 260 will receive care as usual. In order to ensure that participants have an equal chance of receiving housing and supports as part of the study or not, the assignment to one group or another will be random (like a coin toss).

'Housing plus Supports' group:
- Participants assigned to the housing plus supports group will receive a rent allowance (attached to the individual, not the unit) until at least March 2013. The project team is working to extend funding for the services; however, there is a possibility that participants may lose their rent allowance and supports at the end of the project. In this case, a transition plan will be developed for all participants.
- Individuals with high needs will be assigned to an ACT team. Individuals with moderate needs will receive Intensive Case Management. These services will be provided by community-based agencies.
- Treatment will be offered throughout the project but is not mandatory. Participants are not required to be abstinent from drugs or alcohol.

- Participants are required to meet with a case manager involved with the project at least once a week.

'Usual Care' group:

- Participants assigned to the 'Usual Care' group will not receive housing or supports through this study.
- Individuals will still be able to receive any current or future services available in the community for people who are homeless and have mental health issues.
- Participants will be informed by the study team about new and existing services.

Research requirements for both groups:

- Individuals will be asked to update their contact information with the research team monthly.
- Individuals in both groups will be interviewed every 3 months for a period of 2 years from enrollment in the study.
- Interview topics will include: housing quality, physical and mental health status, health care and social service use, and quality of life.
- Interviews will take between 10 to 90 minutes.
- Researchers will use participant health card numbers to look at use of health care services over time (1 year prior to the study and for the 2 year study period).
- All participants will receive an honorarium of $10 to $30 per interview.
- All information collected through the research will be kept strictly confidential.

Participation is voluntary: If, at any time during the study, a participant no longer wants to be involved, they are free to withdraw from the research and (if they are receiving them) the housing and supports.

Eligibility Criteria:

- **Legal adult status** (age 18 or older).
- **Homeless** (staying on the street, shelter)
- **Serious and persistent mental health issues** (i.e., psychosis, severe depression, severe post traumatic stress disorder).
- **Must have unmet mental health care and support needs.**

- Formal mental health diagnosis is not required at the time of referral.
- Concurrent disorder (substance use) is allowed.
- We encourage referrals of **individuals from racialized groups**.

Enrollment will begin in October 2009 and will take up to 18 months.

For more information: please contact Kate Mason, Research Coordinator, at 416-864-6060 ext 6513 or masonk@smh.toronto.on.ca.

Recruitment Script for Service Providers:

The following script and questions are provided to help service providers present the project to potential participants.

Script: *There is a new research project for people who are homeless and have serious mental health issues. The project is evaluating a model of housing and support services that will provide government and other decision makers with evidence about what services work best for homeless people with mental health issues.*

If you are eligible and willing to participate in the study, you could receive independent housing and supports for up to 3 ½ years as part of the study. However, there is also a chance that you may not receive housing or supports even if you agree to participate. In either situation, researchers will want to interview you about your experiences with housing, health care and your physical and mental health status. Some interviews will be very short (about 10 minutes) and others will take about an hour and half. Everyone who participates in the study will be paid a cash honorarium for each interview.

Does this sound like something you're interested in participating in or learning more about?

POSTER → *to be designed and then approved by SMH Public Relations. Please note: poster will not be displayed widely; it is for targeted use in health care provider offices and other staff spaces.*

[MHCC logo] [CRICH/SMH & City of Toronto logos]

-- RECRUITMENT OF PARTICIPANTS --

"AT HOME"
RESEARCH DEMONSTRATION PROJECT
ON MENTAL HEALTH & HOMELESSNESS

The Mental Health Commission of Canada, the Centre for Research on Inner City Health, St. Michael's Hospital and the City of Toronto are conducting a research study to evaluate models of housing and support services for homeless people who have serious mental health issues.

What does the study involve?
- Half of the study participants will receive independent housing and support services (either Assertive Community Treatment or Intensive Case Management) through the study.
- All participants will be interviewed every 3 months for a period of 2 years and will receive an honorarium for each interview.

Who is Eligible?
- 18 years or older
- Homeless
- Serious and persistent mental health issues (formal diagnosis not required at the time of referral)
- Not currently receiving Assertive Community Treatment or Intensive Case Management services

How do I make a Referral?
- For more information or to make a referral please contact Kate Mason, Research Coordinator, 416-864-6060 ext 6513 or masonk@smh.toronto.on.ca.

[Version date: August 2009]

[MHCC logo] [CRICH & City of Toronto logos]

"AT HOME"
Research Demonstration Project in Mental Health & Homelessness:

REFERRAL FORM

Please email, fax or call your referral to Kate Mason, Research Coordinator
Email: masonk@smh.toronto.on.ca
Fax: (416) 864-5558
Tel: (416) 864-6060 ext 6513

Name of person making referral: ______________________________

Agency: ______________________________

Position: ______________________________

Telephone: ______________________

Email: ______________________

Today's Date: ____________________

☐ **I have discussed this study with the potential participant and they have agreed to this referral (please check).**

Potential study participant: First name ________________________

Last name ________________________

Our study is trying to recruit a diverse range of participants. The following information is necessary to ensure that our sample reflects the diversity of the general population of Toronto and of the range of living situations of people who are homeless.

Gender: Male / Female / Trans

Is this person from a visible minority/racialized group? yes / no

Current living situation (e.g. shelter, 'sleeping rough', couch surfing, etc):
____
____

Please provide us with the following additional participant contact details (if contact is not through referral source).

Telephone number(s) where he/she can be reached: ______________

____

What kind of phone number is this? (personal, phone number of a shelter, worker, drop-in, etc). Provide additional names/agency information if applicable: ______________________________

____

Drop-In or Agency where this person spends the most time and where we have permission to go look for him/her: ______________________

____

What type of mental health difficulties does your client experience?:

____

____

____

During the last month have you observed ANY of the following behaviors? Please circle all that apply:

1. *Verbal expression of clearly false or bizarre ideas such as reporting references to self on TV or in newspaper or claiming to be someone he/she clearly is not.*
2. *Appears to or reports hearing sounds of people talking when no one is around, or sees visions others do not see.*
3. *Extremely difficult to understand because odd use of words, rambling, and/or sudden change of topics.*
4. *Indications of extreme sadness together with severe apathy or withdrawal.*
5. *Engages in self harm activities such as slashing and/or frequent overdosing.*

Has your client seen a psychiatrist or been admitted to hospital for mental health issues in the past two years?

Yes No

If yes, please provide the consultation report or discharge summary if available.

Have any of the following difficulties with basic needs in past month been reported or observed? Please circle all that apply:

1. *Needs assistance to meet nutritional needs (e.g., unable to obtain/access meals even when opportunity is available).*
2. *Needs assistance to maintain minimally adequate personal hygiene, (e.g., washing, grooming, oral hygiene).*
3. *Needs assistance or is unwilling to access needed resources (e.g., financial benefits, clothes, appropriate accommodation or medical care).*
4. *Needs assistance to acquire and maintain network of family, friends and peers (e.g., extremely isolated).*
5. *Needs assistance to manage finances (e.g., unable to budget for basic daily living needs).*

Is your client currently in receipt of case management services?

Yes No

If Yes, please specify the case management agency:__________

____

____

Note to referral sources: if the person you refer to our study is randomized to the housing and support services group, your contact information will be shared with their new service team so that a member of the team is able to contact you if they require any additional service referral information.

'AT HOME' RESEARCH STUDY

INFORMATION FOR POTENTIAL PARTICIPANTS

This research is being conducted by the Centre for Research on Inner City Health at St. Michael's Hospital and the City of Toronto. This study is funded by the Mental Health Commission of Canada and is one of five Research Demonstration Projects on Homelessness and Mental Health taking place across the country.

You have been told about this research project because you are experiencing homelessness and might benefit from the services being offered as part of this study.

We are looking for people to participate in our study to evaluate housing and supports for people who are homeless and who have mental health issues. About half of the people who agree to participate in the study will receive housing and supports as part of the study. The other half will not receive housing and supports through the study but will be directed to the usual sources of care in our community. You will have an approximately equal chance of receiving or not receiving housing and supports as part of the study. In either case, we would like to interview you periodically for two years about your housing situation, physical and mental health, use of social services, and well being.

Frequently Asked Questions

All of these questions will also be addressed with you in more detail as part of the informed consent process before you agree to participate in the study.

What is the housing like? Housing will be made available through a rental subsidy which will be guaranteed until March 31, 2013. You will get housing of your choice, such as an independent apartment with your own kitchen and bathroom. If you use drugs or alcohol, you do not have to stop to receive this housing. You will get to choose where you want to live in the City of Toronto.

What kinds of supports are offered? Depending on your level of need, you will receive support through an Assertive Community Treatment team or from an Intensive Case Management team. Support around daily living skills, employment, mental health issues and substance use will be offered but treatment is not required. The only requirement is that you meet with a case manager once a week for the duration of the project.

What happens after the project ends? The project team is working to ensure that funding for the housing and supports continues even after the project ends but there is a possibility that you will have to move at the end of March 2013. If you do, a plan will be put in place to help with your service provider to help you find ongoing supports and somewhere else to live.

What if I don't get assigned to the housing and supports group? The researchers will still meet with you every 3 months to ask you about your life situation, health and service use and you will get a cash honorarium after each of these interviews. The researchers will also make sure you are directed to other sources of housing and supports in the community.

What about privacy? All of the information that you provide to the researchers will be kept completely confidential and will not impact any services you receive through the project or elsewhere. The researchers will ask you for permission to use your health card number to look at your use of health and income assistance over time. Your name will not be associated with any of this information.

What if I agree to participate and then change my mind? You can stop participating in the research at any time with no penalty.

For more information about the study, please contact Kate Mason, Research Coordinator at (416) 864-6060 ext 6513 or 1-800-XXX-XXXX.

Appendix I: Study Information and Consent form

Consent form begins below this line.

Consent to Participate in a Research Study

Title of Research Study: 'At Home' Research Demonstration Project in Mental Health and Homelessness: Main Study

Introduction

Before agreeing to participate in this research study, it is important that you read and understand this research consent form. It includes details that we think you need to know in order to decide if you wish to take part in this study. If you have any questions, please ask a study staff person. You should not sign this form until you are sure you understand the information. Taking part in this research is completely voluntary. You may also wish to discuss the study with a family member, close friend or worker before you sign this form.

Investigators:

All investigators can be reached from Monday to Friday, 9:00 am – 5:00 pm

Principal Investigator: Stephen Hwang
Centre for Research on Inner City Health, St. Michael's Hospital
Tel: 416-864-5991

Vicky Stergiopoulos
Centre for Research on Inner City Health, St. Michael's Hospital
Tel: 416-864-6060 Ext. 3341

Patricia O'Campo
Centre for Research on Inner City Health, St. Michael's Hospital
Tel: 416-864-6060 Ext. 3312

Research Coordinator: Kate Mason
Centre for Research on Inner City Health, St. Michael's Hospital
Tel: 416-864-6060 Ext 6513

Toll-free Study Phone Line: *[1-800-XXX-XXXX]*

Study Funding:
The study is funded by the Mental Health Commission of Canada.

Purpose of the Research
This study is an evaluation being conducted by the Centre for Research on Inner City Health at St. Michael's Hospital and the City of Toronto. We are doing this research study to find out whether certain types of housing and support services help homeless people who have serious mental health issues. We want to find out if these new services work and how much they cost, compared to what is already available in Toronto. The information from this study may help improve services for homeless people who have serious mental health issues.

Description of the Research
About 560 homeless people in Toronto will participate in this study. As part of the study, about half of these people will receive support services and a housing allowance. The other half will not receive any new services or a housing allowance through the study. In order to determine if and how the support and housing model works, we need to compare people who are receiving this type of housing with supports with people who are not.

If you are eligible for the study and you agree to participate, you will be randomly selected to receive or not receive the new support services and housing allowance. This selection will be done by chance, like the flip of a coin.

Whether you are in the group that receives housing and supports or not, everyone who participates in the study will be interviewed every few months for two years. You will be asked questions about their housing situation, physical and mental health, use of drugs and alcohol, use of social services, and well being.

If you are assigned to receive the support services and housing allowance, the kind of support service you receive will depend on your level of need which will be assessed at an interview within the next week or so. If you have high needs, you will be assigned to an Assertive Community Treatment (ACT) team. If you have moderate needs, you will be given Intensive Case Management (ICM) services. Either way, you must be willing to have a service provider visit you at a mutually convenient time and place at least once a week.

If you have high needs and you are assigned to ACT, your support team will be a group of people who are trained in social work, counseling, nursing and psychiatry. You will be able to contact someone from this team or crises support services 7 days per week, 24 hours per day. If you have moderate needs, you will be assigned to a case manager who will provide or coordinate support services for you 5 days a week, 8 hours per day with after-hours crisis support as needed. Treatment will be offered throughout the project but is not mandatory. Community-based agencies will coordinate the support services.

One of the intensive case management teams is designed specifically for people from specific ethno-racial groups. If you are a member of a racialized group, you may be served by this ethno-racial specific team or by a mainstream intensive case management provider, depending on your preference and availability of service. This does not include aboriginal people who will be served by the regular intensive case management team.

If you are randomly assigned to receive the new support services and housing allowance you will get a rent allowance and help to find housing. You will have choices about where you would like to live, as long as it meets standards for things like safety and affordability. You will receive the support services and rent allowance until at the end of March 2013. Before then, we will work with you to develop a plan to make sure that you will continue to have adequate housing and supports after the study ends. It is possible that you will have to move at the end of the study.

If you are not assigned to receive new services and a housing allowance, you will still be able to use any of the services that you use now. Being in this study will not affect your ability to obtain any other services that are currently available or that become available to you in the future. We will give you information about health care and housing services that already exist in Toronto for people who are homeless.

No matter which group you are randomly assigned to, if you agree to participate in the study, we will interview you within the next week for about an hour and a half. After that, every 3 months for a period of 2 years someone from the study will interview you by phone or in person for either 10 minutes or 1 hour. These interviews will be surveys where researchers will ask you questions and type your answers directly onto laptops that are used only for the study. At the beginning of the study and every 6 months, we will also take your blood pressure and measure your height, weight, hips and waist. As well, we will ask you to contact us once a month to give us a quick update about your living situation. We will give you a toll-free phone number that you can use for these phone calls.

You may also be asked to participate in some additional interviews so that we can hear more about your experiences and stories in your own words. If and when that happens, we will give you more information at that time so that you can decide then whether or not you want to participate in this extra part of the study.

Study follow-up

If you agree to participate in the study, we will ask you to give us the **names and contact information** of friends, relatives, service providers or other people who might know where you are if we are not able to get in touch with you when it is time for your interview. We will only be asking these people about your location and contact information when we don't know where you are and are trying to find you to schedule another interview. We will tell these individuals that we are trying to get in touch with you for the purpose of a research study but we will not give them any details about the study or any of the information you have provided in interviews.

Additional information we would like to collect

In addition to getting information from you directly, with your permission we would also like to be able to get information from various government databases and your health care providers about your use of health care services, shelter services, income assistance, and the justice system. We would like this additional information to be able to measure the costs associated with providing housing and support services and to understand exactly what the impact of these services might be. Specifically, we would like to obtain information from the following sources:

1) **Ministry of Health and Long Term Care.** We would also like to have your permission to use your name, date of birth, and Ontario health card number to get information about your **use of health care and prescription drugs** from the Ontario Ministry of Health records. We will only be looking at what kinds of drugs you have been prescribed, the kinds of health care services you use and how often you use these services. If you don't know your health card number or don't have a health card, we will find out what your number is for you from one of your health care providers or help you get a health card.

2) **Ministry of Community and Social Services.** We would like to have your permission to use your name and date of birth or your Ontario Works or Ontario Disability Support Program (ODSP) number, if you have one, so that we can get information about what **income assistance** you might be receiving and what your qualifying disability is if you are receiving ODSP. With your permission, we would also like to be able ask the Ministry of Community and Social Services for your updated contact information so that we can get in touch with you for follow-up interviews. If you have a payee, we would like your permission to obtain their contact information from the Ministry of Community and Social Services so that we can contact them to find out about your whereabouts to follow up with you and schedule more interviews.

3) **Correctional Service of Canada, Ministry of Community Safety and Corrections and Toronto Police Service.** We would like your permission to use your name and date of birth to obtain information about any contacts you have had with **police and the justice system**.

4) **City of Toronto Shelter, Support & Housing Administration Division.** We would like to ask for your permission to use your name and date of birth so that we can get information about your **use of the Toronto shelter system** through the City of Toronto's shelter management and database system.

5) **Health Care Providers**. With your permission, we would also like to review your **health charts** that are kept at your doctor's office, clinics, or the hospitals you've been to the most in recent years. We would like to look at your health records in these places to learn more about your physical and mental health status and find out if you are receiving certain physical health tests and services.

6) If you are assigned to receive the new support services and housing allowance, we would like your permission to obtain **information from your new service provider** about the amount and type of service you receive.

Potential Risks

It is possible that if you are not randomly assigned to receive the housing allowance and new services that you will feel disappointed. If you are not assigned to receive new services and a housing allowance, we will provide you with information about services that already exist in Toronto for people who are homeless and would like mental health support.

During the study, it is possible that you may feel some discomfort during interviews because you may be asked about personal health experiences, which may have been painful or upsetting. You do not have to answer every question and you can ask to stop the interview at any time.

Potential Benefits
If you are randomly assigned to the support services and housing allowance group you will receive a rental allowance and support services until March 31, 2013 and possibly beyond but only if continued funding becomes available.

People who are not assigned to this group may not benefit directly from participating in the study. However, we are conducting this research because we plan to use the information we collect to improve health and social services for homeless people. We hope that in the long run all homeless people in Toronto will benefit from any changes made because of the findings of this study.

Protecting Your Health Information
The information you provide for this study will be kept strictly confidential. St. Michael's Hospital is collecting the study data but, for the most part, it is being stored and protected by a company called HealthDiary who we have contracted with for this purpose. HealthDiary has a strong track record in providing similar systems for other health research studies. To protect your privacy, any information that could identify you (such as your date of birth and provincial health card number) will be kept separately from the rest of the information you provide to us over the course of the study and coded. All of the information you give to us will be kept in a secure research office and only authorized research staff will have access to the information. All of the paper data will be stored in locked filing cabinets that only authorized members of the research team will have access to. All of the electronic data will be kept on a secure server in an unreadable format for anyone outside of the study. All study information will be kept for a period of 10 years from the end of the study and then destroyed.

The results of any research will include information from many people grouped together so that no one person can be identified. No records or personal information that could be linked to you will ever be reported. The principal investigators will protect your records and keep all your information confidential to the greatest extent possible. The chance that your information will accidentally be given to someone outside the study is small. No information that reveals the identity of participants will be released or published without your consent, unless required by law. For example, we would have to breach your privacy and make sure that you get treatment, or that protective action is taken, if we receive information that a child is being abused, or that you are at high risk of physically harming yourself or someone else.

The Research Ethics Board at St. Michael's Hospital may need to review study records for monitoring purposes. As part of this review, someone may contact you from the Research Ethics Board to discuss your experience in this research study.

Study Results
If the results of this study are published in articles or presented at conferences, seminars or other public forums, no individual information that could identify you will be released. Note that the study investigators may choose to not publish or make use of the results of the study.

Reimbursement to Participants
If you agree to participate in the study, you will be reimbursed for any reasonable expenses related to your participation in the research components of the study, such as transportation. You will be given $10 for each month that you call us to update us on your living situation and for the short interviews. For the longer interviews that occur every 6 months, you will be given $25. For the final interview at the end of the 2-year study period, you will receive $30. Payment for each completed interview stage will be made to you whether or not you complete the entire study. There are no costs to your participation in the study.

Participation and Withdrawal
Participating in this study is completely voluntary. If you decide to participate, you can still refuse to answer any questions, or stop the interviews at any time.

If you are receiving the housing allowance and support services, you will not lose your housing or services even if you decide you do not want to participate in the study. A researcher or your service worker will ask you if you would like to re-join the study from time to time, but the decision is yours. You are not obligated to re-join the study. Although you can withdraw from the study at any time, the information that you have provided us up until the time that you leave the study will still be kept for research purposes.

Your decision to participate or not will have no impact on the care you receive now or in the future from St. Michael's Hospital, the study service providers or any other health and social service provider in the city.

Research Ethics Board Contact
The study protocol and consent form have been reviewed by a committee called the Research Ethics Board at St. Michael's Hospital. The committee is established by the hospital to review studies for their scientific and ethical merit. If you have any questions regarding your rights as a research participant, you may contact Dr. Julie Spence, Chair, St. Michael's Hospital Research Ethics Board at (416) 864-6060 ext. 2557 during business hours.

The Research Ethics Board at St. Michael's Hospital may need to review records for monitoring purposes. As part of this review, someone may contact you from the Research Ethics Board to discuss your experience in the study.

Study Contacts
If you have any questions about this study, contact Kate Mason, Research Coordinator, Centre for Research on Inner City Health, St. Michael's Hospital at: Telephone: 416-864-6060 ext 6513, or one of the principal study investigators listed on page one of this consent form.

"AT HOME" RESEARCH DEMONSTRATION PROJECT IN MENTAL HEALTH & HOMELESSNESS: MAIN STUDY

I acknowledge that the research study described above has been explained to me and that any questions that I have asked have been answered to my satisfaction. I have been informed of my right to choose to not participate in the study. As well, the potential risks, harms and discomforts have been explained to me and I also understand the benefits of participating in the research study. I understand that I have not waived my legal rights nor released the investigators, sponsors, or involved institutions from their legal and professional duties. I know that I may ask now or in the future any questions I have about the study or the research procedures. I have been assured that information relating to me and my health care will be kept confidential and that no information will be released or printed that would disclose my personal identity without my permission unless required by law. I have been given enough time to read and understand the above information.

By signing this consent, I agree to participate in this study. I will be given a signed copy of the entire consent form, including this signature page

X____________________	________________	__________
Signature of Participant	Name (printed)	Date
X____________________	________________	__________
Signature of Study Person Explaining Study	Name (printed)	Date

CONSENT TO OBTAIN PROVINCIAL HEALTH CARD NUMBER FROM SERVICE PROVIDERS ☐ not necessary

☐____ (initial) I do not have my health card in my possession at the time of this interview and give permission for the researchers to contact my recent health care providers to obtain my health card number from them for the purposes of the study.

☐____ declined

CONSENT THE RELEASE OF HEALTH CARE USE INFORMATION

☐____ (initial) I agree to allow the research team to link my provincial health card number or my name and date of birth to Ministry of Health files to obtain information about my use of health care services and prescription drugs for the past year and for the next 5 years.

☐____ declined

CONSENT TO THE RELEASE OF INFORMATION FROM THE MINISTRY OF COMMUNITY AND SOCIAL SERVICES

☐____ (Initial) I agree to allow the research team to link my name, date of birth, and either Ontario Disability Support Program (ODSP) or Ontario Works number to databases at the Ministry of Community and Social Services (MCSS) so that the research team can determine my welfare and disability benefit amounts, my qualifying ODSP diagnosis, for the past 1 year and in the next 5 years. I give MCSS permission to release my updated contact information or the contact information of my payee/trustee if I have one to the research team so that they may follow up with me during the study period.

☐____ declined

CONSENT TO THE RELEASE OF INFORMATION FROM CORRECTIONAL SERVICE OF CANADA, MINISTRY OF COMMUNITY SAFETY AND CORRENCTIONS, & TORONTO POLICE SERVICE

☐____ (Initial) I agree to allow the research team to link my name and date of birth to federal and provincial corrections data bases and with Toronto Police Service databases so that the research team can obtain information about my use of the justice system and contacts with the Toronto police, for the past 1 year and in the next 5 years.

☐____ declined

CONSENT TO THE RELEASE OF INFORMATION FROM THE CITY OF TORONTO'S SHELTER MANAGEMENT INFORMATION SYSTEM

☐____ (initial) I consent to the use of my name and date of birth by the City of Toronto's Shelter Management Information System. I authorize the City of Toronto to provide the research team with information about dates and locations of my shelter use throughout Toronto for the past year and for the next 5 years.

☐____ declined

CONSENT TO THE RELEASE OF HEALTH RECORD INFORMATION BY HEALTH CARE PROVIDERS

☐____ (initial) I consent to the research team contacting my family doctor and other health care providers that I have used recently for the purpose of obtaining information from my health records about my physical or mental health diagnoses, health services I have been offered, and the results of physical health tests I may have had for the 3-year period before the study and for the study period. I understand that the research team will only look at the health records of some people in the study and that it is possible that they may not need to access my health records even though I have given permission for them to do so.

☐____ declined

CONSENT TO THE RELEASE OF INFORMATION FROM ACT/ICM SERVICE PROVIDERS & HOUSING ADMINISTRATION IF ASSIGNED TO THE INTERVENTION GROUP

☐____ (initial) If I am assigned to receiving housing and support services, I give permission for the research team to contact my ACT/ICM service provider and the project's housing administrator (Housing Connections) for the purpose of obtaining information about the amount and type of service I receive, my housing status and my position on the social housing wait list.

☐____ declined

WITNESS DECLARATION OF PARTICIPANTS INFORMED CONSENT: *If a participant is unable to read independently for any reason or unable to sign, a witness should be present during the entire informed consent discussion and sign below. The witness may be a family member who is able to read. By signing the consent form I attest that the information was accurately explained to and apparently understood by the participant and that consent was given freely.

____________________ ________________________ _____________

Name of Witness Signature of Witness Date

INTERPRETERS DECLARATION AND SIGNATURE:

I have been requested to interpret the consent discussion for the potential research participant [Name of Potential Participant]: __________________________________

I am competent in the English language and in the language of the choice of the potential participant [Language]: ____________________________

I agree to keep confidential all personal information of the potential participant.

I have interpreted the consent discussion. The potential participant has advised me in his/her own language that he/she has been informed about the research study, the nature and extent of his/her participant, including the risk involved. The potential participant freely gives his/her consent to participate in this study.

Signature of Interpreter: ____

Printed name of interpreter: ____

Relationship or Position of Interpreter: _______________________________________

Contact Information of Interpreter: ____

Date: _____________

Signature page for authorization to obtain health record information from health care providers

Dear Health Care Provider,

Re: ______________________________ ________________________

Name of Participant Date of Birth [DD/MM/YY]

This is to authorize you to give Dr. Stephen Hwang and the study team access to the following information and data which they request regarding my physical and mental health status, for use in the research study entitled: "**'At Home' Research Demonstration Project in Mental Health and Homelessness: Main Study**". This information will be obtained via chart review by one of the study staff. The information extracted will include: physical or mental health diagnoses, preventative health services, and the results of physical health tests, for the 3-year period before the study and for the 2 year study period. One chart review will take place as soon as possible and the second will take place 18 months from now.

X____________________________ ________________________ ______________

Signature of Participant Name (printed) Date

[This page will be signed up to 7 times – one copy stored in the research study file, one copy given to participant and up to 5 copies to be sent to the identified health care provider(s)]

Appendix K: Contact Follow-up Script

'AT HOME' PROJECT
SCRIPT FOR CONTACTING PARTICIPANTS' FRIENDS, FAMILY, SERVICE PROVIDERS

Can I speak with [contact name] please?

Good (morning/afternoon). My name is [name] I am a [position] from the Centre for Research on Inner City Health. I am calling you because [participants name] gave us your name and number as a contact person while participating in a research study with us.

[participants name] did a interview with us on [month/year] but (his/her's) contact information changed after that. We need your assistance to get in contact with (him/her) for another interview. Can you provide us with his current contact information or give to (him/her) our contact information so that (him/her) can contact us to schedule (his/her) next interview?

(If contact asks about the study): The study is about housing and support services.

(Give and/or take note of contact information)

Thank you very much for your help. We might contact you again in the future if we are unable to contact [participants name] for another interview. If you have any question feel free to contact (me OR the study research coordinator), (my/her) phone number is 416 864-6060 x 6513.

INTERVENTION:

Service providers
Service providers involved in the delivery of the intervention were chosen through a widely released Request for Expressions of Interest. COTA Health will provide the Assertive Community Treatment (ACT) services, Toronto North Support Services/Community Resource Connections of Toronto will provide the Intensive Case Management (ICM) services, and Across Boundaries will provide the Ethno-Specific Intensive Case Management (ES-ICM).

The City of Toronto's Shelter, Support and Housing Administration Division will administer the rent allowances and provide housing support to the ACT and ICM teams.

Rental Allowance/ Housing First Model
Participants in the intervention group will receive a rent allowance which will be paid directly to the landlord. However, the participant will be named on the lease with all the rights and obligations entailed under provincial legislation. Maximum rents will be based on the total of the study subsidy ($600) plus the shelter component of participant's income support payments (ODSP/OW) for a rent that includes utilities. The maximum rent will be reduced where utilities are excluded. The study budget also includes an allowance for furnishings and move-in costs. To ensure the greatest range of housing choices, outreach to landlords will be undertaken to identify available housing. Participants will be able to choose their own housing after it meets minimum City of Toronto standards for acceptability, including property standards and being self-contained. Housing will be offered within a "Housing First" framework.

"Housing First" is a model of housing provision that provides immediate access to permanent housing and support services, within a philosophy of consumer choice. Unlike other supportive housing models, clients are not required to participate in treatment services or main sobriety from drugs or alcohol as a provision of housing. Housing is provided primarily in scattered site private market apartments. Treatment and support services are provided off-site (i.e. are not attached to the housing) and are focused on helping people to maintain housing and integrate into the community by addressing mental health and recovery goals.

Assertive Community Treatment
The Assertive Community Treatment (ACT) team will provide integrated, community-based recovery-oriented support, treatment and rehabilitation. ACT teams use a team approach to treatment; although each client will be assigned a primary service coordinator who will coordinate and monitor the activities of the ACT team and the client's personal support network in relation to supporting each client's pursuit of their individual Recovery/Goal Plans. Many services are provided directly by team members whose backgrounds and training include social work, rehabilitation, counseling, nursing and psychiatry. Among the services ACT teams provide are: case management, initial and ongoing assessments; psychiatric services; employment and housing assistance; family support and education; substance use services; and other services and supports critical to an individual's ability to live successfully in the community. Participants will have access to someone from this team or crises support services 7 days per week, 24 hours per day.

ACT in the Housing First context of this study differs from other ACT models with its recovery-orientation and access to housing. A key adaptation of the general ACT model within housing first is in its strength-based, person-centred approach to treatment and its aims for participants' self determination and independence.

Intensive Case Management

Intensive Case Management (ICM) is a comprehensive, coordinated approach to client-centred services. Each study participant will be matched with a Case Manager. The staff to client ratio is generally 20:1. The Case Manager will works in partnership with each participant to develop a service plan that meets the individual's needs and is acceptable to him/her. Together they will work on achieving goals that are important to the client through any of the following functions: outreach, assessment, skills teaching, network development, linking to resources, individual advocacy and crisis support. Service will take place in the community, typically at the location of choice for the participant. The exception to this will be the availability of some group programming at the Project Office, in combination with the ACT Team. Frequency of contact will be negotiated with each participant and will vary over time. It is anticipated that most clients will receive one or two contact per week initially, but there will be capacity for more frequent contact for short periods when there is a need. Case managers will provide support services for participants 5 days a week, 8 hours per day, with after-hours crisis support as needed.

ICM in the Housing First context of this study differs from other ICM models as its interventions are anchored in independent living (many ICM programs do not have the resources to access housing on behalf of their clients) and has a focus on providing supports for increased personal independence over time, without creating a dependent relationship.

Ethno-Specific Intensive Case Management

The Ethno-Specific Intensive Case Management model will provide the same basic support services as the regular ICM model but will emphasize a holistic approach to mental health care that recognizes the interdependence of spiritual, emotional, mental, physical, social, economic, cultural, linguistic and broader environmental aspects of life including social determinants of health in working with individuals with severe mental health problems/illness.

Programs and initiatives that integrate peer support, skills building, social and recreational activities, support groups, alternative and complementary therapies, art and music therapy, creative expressions, community kitchen, individual support and community outreach will be available to participants in this intervention group. Assisting participants to build their support networks, including working with their family and friends will be a main goal of this model.

The ES-ICM service will be provided from 8am- 8pm, 7 days /week based on client need. Aside from the ability of case managers to speak a second language, translation and interpretation services will be purchased as needed.

CLASSIFICATION OF HIGH (ACT) AND MODERATE (ICM) NEED:

Study participants will be classified as high or moderate need using criteria that correspond to Section 3 of the Ontario Standards for Assertive Community Treatment teams. Study participants who meet two or more of the criteria will be determined to be high need. All other study participants will be classified as moderate need.

a) high use of schedule one hospital services or specialty hospital services, tertiary level services, or psychiatric emergency services such as mental health crisis response services.
b) intractable (i.e. persistent or very recurrent), severe major symptoms (e.g. affective, psychotic, suicidal, delusional).
c) co-existing substance use disorder of significant duration (i.e. greater than six months).
d) high risk or recent history of criminal justice involvement (e.g. arrest and incarceration).
e) inability to meet basic survival needs
f) residing in an inpatient bed or in a supervised community residence, but clinical assessed to be able to live in a more independent living situation if intensive services are provided, or requiring a residential or institutional placement if more intensive services are not available.

USUAL CARE:

It is anticipated that high and moderate need clients in the control groups will have access to several of the programs and services described below, and will continue to be directed to both mainstream and homeless specific health services for care. Individuals suffering from a serious and persistent mental illness enter the treatment system in Toronto through many doors: inpatient and outpatient clinical service, peer support, crisis services, intensive case management, Assertive Community Treatment Teams (ACT), supportive housing, vocational programming, street outreach programs, drop-in services and shelters. Although it can be argued that Toronto has a wealth of mental health services provided by several dozen organizations, it has been estimated that only about 25%-50% who meet the criteria for services at any given time actually find their way to receiving them.

Currently, homeless health and housing resources in Toronto with a component related to mental illness include:

<u>ACT Teams with Homelessness Focus:</u> There are 13 Assertive Community Treatment teams in the greater Toronto area, serving over 1,000 individuals with severe mental illness. Nine teams are hospital based, and some, such as the CONTACT team at St. Michael's Hospital, have a homelessness focus. There is one ethno-specific ACT Team in Toronto, a joint venture between Mount Sinai Hospital and Hong Fook Mental Health Association.

<u>Community Health Centers</u>: People experiencing homelessness have access to all of the primary care and support services offered by the Community Health Centres. Community outreach programs attempt to connect these clients with a community health centre or other primary health care provider. In cases where a client is unwilling to visit a health centre, the outreach workers can arrange for CHC nursing services to visit the client. After 12 years of having a specific

envelope of funding to serve homeless people, caseloads of people who are experiencing homelessness have become high and the capacity to serve more clients has become limited. In response to this challenge the CHC's have established walk-in clinics and drop-in sites for people who are homeless because these help reduce barriers (e.g. difficulty keeping appointments) that homeless people face when trying to access health care.

Emergency Rooms in Hospital Facilities: Toronto is home to 12 general hospitals and one provincial psychiatric hospital. The Rotary Club of Toronto Transition Center at St. Michael's Hospital serves approximately 1,000 homeless individuals annually, offering a temporary refuge and help in accessing community resources.

Hostel Outreach Program (providing intensive case management services): The Hostel Outreach Program (HOP) operates out of two organizations, CRCT and COTA. HOP engages clients by developing relationships with them, building on trust and respect. Clients determine their goals, which include but are not limited to housing, health, re-unification with their families, safety, social isolation and income.

Drop-Ins with Onsite Medical and Psychiatric Services: Most drop-ins have very limited resources for providing psychiatric or medical supports to their homeless service users living with serious mental health issues. A few larger drop-ins in Toronto, primarily those with Ministry of Health funding (of which there are six), have the resources to provide more extensive medical and case management supports to their homeless clients living with serious mental illness.

Ethno-Cultural Services: Toronto has a number of ethno-racial services providing mental health care. These range from mainstream mental health providers who run specific initiatives targeted at ethno-racial groups (Canadian Mental Health Association, Mount Sinai Hospital, Sistering, Toronto Western Hospital) to purely ethno-specific mental health service providers. Examples of the latter include Access Alliance, a community health centre for immigrants and refugees; Across Boundaries, a mental health center for people of color; and the Canadian Mental Health Association, offering a variety of cross-cultural initiatives. Services for ethno-specific groups will be evaluated in the third arm of this study.

Specialized Health Care Teams: These include: Inner City Health Associates, offering primary care and psychiatric clinics in shelters, drop-ins, street outreach teams and supporting housing agencies (25 locations), Seaton House Family Health Team, offering shelter based primary and mental health care and Shared Care Teams/CAMH, offering physician and nursing/case management services in 5 locations.

Specialized Health Care Facilities: Sherbourne Health Centre Infirmary offers a site for short-term recuperation for homeless individuals with medical conditions, usually after discharge from hospital. Perram House offers hospice care with a special emphasis on serving homeless and marginally housed individuals.

Shelters with Health Services: About 60% of shelters have some health care providers coming into the program to offer services to clients. Most shelters have general practitioners and registered nurses, and some have specialized services such as psychiatrists. Other shelters have established referral relationships with community health centres and specialized health care facilities such as the Sherbourne Health Centre.

Shelter Programs: The City of Toronto supports about 3,031 beds (excluding families) in 59 shelters, in addition to the Assessment and Referral Centre (overnight respite and shelter access service. Almost all shelters operate on a 24/7 basis and offer a wide range of services in addition to meals and a place to sleep. Approximately 75% of shelters have onsite case management teams that support and guide clients through the multiplicity of systems that include health, income supports, housing, legal, training, education and employment. Many shelters also have established relationships with housing providers, community case management agencies, health services and income support services. Some shelters as part of multi-service agencies also have direct access to housing and employment centres. Case workers in shelters, regardless of their specialty, will assist clients to transition to living in the community by supporting them to access public transportation, furniture, food banks and social networking as is found in drop-ins.

Private & Social Housing: Housing for low income and homeless individuals in Toronto is obtained through a range of possible avenues. Many community-based agencies, shelters and health care providers (such as social workers at Community Health Centres or discharge planners at hospitals) have workers who will assist homeless people to find either private market or social housing. The City of Toronto's Streets to Homes program is mandated to seek out and engage individuals who are living rough on the street and assist them to find housing. An average market-rent bachelor apartment in Toronto costs about $740. Welfare benefits for a single person in Toronto are approximately $548 per month while disability benefits are approximately $979. Many people with serious and persistent mental health issues are eligible for disability benefits and are able to find housing once these benefits are secured [8].

Homeless and low income indivudals may also access social or 'rent geared to income' housing where the portion of one's income that goes towards rent is not more than 30%. The City of Toronto provides social housing to approximately 164,000 low and moderate-income tenants including seniors, families, singles, refugees, recent immigrants to Canada and people with special needs. The average wait for a subsidized bachelor unit in Toronto is one to five years and up to ten years for a one- to five-bedroom home. There are approximately 4,405 supportive housing units in Toronto specifically designated for individuals with persistent mental illness. The great majority of these are permanent housing with support varying from one hour per week to 24 hours per day. Additionally there are 3,422 units available through what is referred to as the "alternative housing providers," a group of 33 providers who house individuals with a variety of health and social issues. Alternative and supportive housing takes a number of forms including self-contained apartments and private rooms in shared accommodation where kitchens, bathrooms, living rooms and kitchens are common. Alternative housing support services include: housing stabilization, eviction prevention, crisis intervention, referrals, employment, and health care including addictions and mental health services; assistance with budgeting, community living and personal care; conflict mediation.

Appendix M: Question 3B - Additional Co-Investigators

6	Title: Research Scientist	Last Name: Sridharan	First Name: Sanjeev	Institution: St. Michael's Hospital
	Dept/Div: Centre for Research on Inner City Health	Program:	Signature	
7	Title: Associate Professor	Last Name: Kirsh	First Name: Bonnie	Institution: University of Toronto
	Dept/Div: Occupational Science & Occupational Therapy	Program:	Signature	
8	Title: Research Scientist	Last Name: Nisenbaum	First Name: Rosane	Institution: St. Michael's Hospital
	Dept/Div: Centre for Research on Inner City Health	Program:	Signature	

Appendix N: Study Information and Consent form: Personal Narratives

Consent form begins below this line.

Consent to Participate in a Research Study

Title of Research Study: 'At Home' Research Demonstration Project in Mental Health and Homelessness: Personal Narratives Component

Introduction

Before agreeing to participate in this research study, it is important that you read and understand this research consent form. It includes details that we think you need to know in order to decide if you wish to take part in this study. If you have any questions, please ask a study staff person. You should not sign this form until you are sure you understand the information. Taking part in this research is completely voluntary. You may also wish to discuss the study with a family member or close friend.

Investigators:

All investigators can be reached from Monday to Friday, 9:00 am – 5:00 pm

Co-Principal Investigators: Stephen Hwang
Centre for Research on Inner City Health, St. Michael's Hospital
Tel: 416-864-5991

Vicky Stergiopoulos
Centre for Research on Inner City Health, St. Michael's Hospital
Tel: 416-864-6060 Ext. 3341

Patricia O'Campo
Centre for Research on Inner City Health, St. Michael's Hospital
Tel: 416-864-6060 Ext. 3312

Research Coordinator: Kate Mason
Centre for Research on Inner City Health, St. Michael's Hospital
Tel: 416-864-6060 Ext. 6513

Field Office: *[Site not yet selected – Address will be inserted here]*

Toll-free Study Phone Line: [1-800-XXX-XXXX]

Study Funding:

The study is funded by the Mental Health Commission of Canada.

Purpose of the Research
This research is part of the "At Home" Research Demonstration Project in Mental Health and Homelessness that you are already participating in. The purpose of this part of the research is to understand the lived experiences of study participants in their own words through life story interviews. This component of the research is intended to complement the more statistical information being collected in the rest of the study.

Description of the Research
In this part of the study, we are inviting you to participate in a set of two longer interviews. The first interview will happen today, and the second interview will happen 18 months from now. The interview will be conducted by a member of the Toronto site research team. The interview will last about 1.5 to 2 hours.

During the interview, the interviewer will ask you a number of questions about your life, including questions about particularly memorable life events. With your permission, the interviewer will audio record the interview. There is no deception involved in the research project

Potential Risks
We do not believe that you will experience any significant risks to your well-being by participating in this interview. However, it is possible that if you have had a negative experience in your life, that you may find yourself becoming upset recalling such an experience. Please remember that you can take a break or stop the interview at any time.

Potential Benefits
You may experience some benefits from your participation in this study. First of all, you may enjoy and find it interesting to reflect back on your life and some of the experiences you have had. We are conducting this research because we plan to use the information we collect to improve health and social services for homeless people. In the long run, we hope that your experiences will be useful in improving services for people experiencing mental health and homelessness issues.

Protecting Your Health Information
Your responses to the interview questions will be kept confidential. That is, your name will not be associated with anything you say during the interview. We will keep everything you say confidential and private. A paper transcript of the interview will be identified by code number and stored in a locked filing cabinet to protect the confidentiality of your responses. Should you consent to the use of your quotations, they may be used in write-ups and/or presentations on this research; however, the quotations will not contain any information that allows you to be identified.

All audio files and transcripts of digitally recorded interviews will be stored on a secure (password protected) web-server, which is accessible only to members of the Toronto site research team and the national research team. Transcriptions of the interviews will be stored in a locked filing cabinet at the Centre for Research on Inner City Health, at St. Michael's Hospital. All audio files will be deleted and paper transcripts destroyed by December 31, 2016.

Study Results

A summary of the results of this research will be sent to you when the data have been analyzed. Information from this research will be used to inform reports on the experiences of individuals who have experienced mental homelessness and health issues at each of the five sites that are part of this study, as well as a cross-site report developed by the national research team. Additionally, we plan to present the results of the research at professional and scientific conferences and to publish the findings in professional and scientific journals. Note that the investigators may choose to not publish or make use of the results from this study.

Reimbursement to Participants

If you agree to participate in the study, you will be reimbursed for any reasonable expenses related to your participation in the research components of the study, such as transportation. For your participation in this interview, you will receive compensation in the amount of $25.

Participation and Withdrawal

Your participation in this study is purely voluntary and you have the right to decide that you do not want to take part in the research. Your decision to take part or to not take part will in no way affect your usual educational, health or community services. We will ask you to participate in a second interview that is similar 18 months from now. If you withdraw from the study, we will not transcribe any of your responses to the interview. You have the right to withdraw your response to any question without penalty. You can stop the interview at any time, and you will still receive compensation even if you don't answer all of the interview questions. If you decide to stop the interview or withdraw from the study at any time, the information that you have provided us up until that time will still be kept for research purposes. During the interview there may be things that have been discussed which you may have concerns about. If you have any questions or concerns about yourself or your family, the interviewer will distribute to all participants a list of names and phone numbers of people and agencies in your community that can assist with any questions or concerns that you might have.

Research Ethics Board Contact

The study protocol and consent form have been reviewed by a committee called the Research Ethics Board at St. Michael's Hospital. The committee is established by the hospital to review studies for their scientific and ethical merit. If you have any questions regarding your rights as a research participant, you may contact Dr. Julie Spence, Chair, St. Michael's Hospital Research Ethics Board at (416) 864-6060 ext. 2557 during business hours.

The Research Ethics Board at St. Michael's Hospital may need to review records for monitoring purposes. As part of this review, someone may contact you from the Research Ethics Board to discuss your experience in the study.

Study Contacts

If you have any questions about this study, contact Kate Mason, Research Coordinator, Centre for Research on Inner City Health, St. Michael's Hospital at: Telephone: 416-864-6060 ext 6513, or one of the principal study investigators listed on page one of this consent form.

"AT HOME" RESEARCH DEMONSTRATION PROJECT IN MENTAL HEALTH & HOMELESSNESS: PERSONAL NARRATIVES COMPONENT

Consent:
I acknowledge that the research study described above has been explained to me and that any questions that I have asked have been answered to my satisfaction. I have been informed of my right to choose to not participate in the study. As well, the potential risks, harms and discomforts have been explained to me and I also understand the benefits of participating in the research study. I understand that I have not waived my legal rights nor released the investigators, sponsors, or involved institutions from their legal and professional duties. I know that I may ask now or in the future any questions I have about the study or the research procedures. I have been assured that information relating to me and my health care will be kept confidential and that no information will be released or printed that would disclose my personal identity without my permission unless required by law. I have been given enough time to read and understand the above information.

By signing this consent, I agree to participate in this study. I will be given a signed copy of the entire consent form, including this signature page

X________________________	__________________	__________
Signature of Participant	Name (printed)	Date
X ______________________	__________________	___________
Signature of Study Person Explaining Study	Name (printed)	Date

I agree to have the interview tape-recorded.

Yes _______ No _______ Initials: _______

WITNESS DECLARATION OF PARTICIPANTS INFORMED CONSENT: *If a participant is unable to read independently for any reason or unable to sign, a witness should be present during the entire informed consent discussion and sign below. The witness may be a family member who is able to read. By signing the consent form I attest that the information was accurately explained to and apparently understood by the participant and that consent was given freely.

______________________	___________________________	_______________
Name of Witness	Signature of Witness	Date

INTERPRETERS DECLARATION AND SIGNATURE:

I have been requested to interpret the consent discussion for the potential research participant [Name of Potential Participant]: ____

I am competent in the English language and in the language of the choice of the potential participant [Language]: ________________________________

I agree to keep confidential all personal information of the potential participant.

I have interpreted the consent discussion. The potential participant has advised me in his/her own language that he/she has been informed about the research study, the nature and extent of his/her participant, including the risk involved. The potential participant freely gives his/her consent to participate in this study.

Signature of Interpreter: ____

Printed name of interpreter: ____

Relationship or Position of Interpreter: ____

Contact Information of Interpreter: ____

Date: _______________

Appendix O: Screening Consent Script

'AT HOME' STUDY: SCREENING CONSENT SCRIPT

To be read to all participants prior to beginning the screening form.

Before we begin the process of finding out if you are interested and willing to participate in this study, I need to ask you some questions to first find out if the study is appropriate for you and your life situation. This will be determined by an interview that will last about 30 minutes. I will be asking you some questions about your mental health, access to health care services and your living circumstances. Your answers will be kept strictly confidential but you can also choose not to answer any specific question if you wish, just let me know.

The screening process is completely voluntary. Your decision to participate or not participate in this screening interview will have no effect on the services you receive at St Michael's Hospital or elsewhere in the community.

You will not benefit directly from this screening. There is no honorarium. It is possible that you may feel some discomfort during the screening because you may be asked about personal health experiences, which may have been painful. You can skip any question you don't want to answer and you can ask to stop the interview at any time.

Before you begin, I would like to let you know that, with your permission, we will be keeping the information that you provide during this brief interview and the information that was provided to us by the person or agency that referred you to the study.

We would like to keep this information in case, if for any reason, you have to be screened for the study again, we will not have to repeat every single question with you and that will save time. We would also like to keep this information so that we can get a better understanding of who wasn't able or decided not to join our study.

All of the information that you provide in the screening interview will be kept confidential and stored securely. Any information such as your initials and date of birth that could identify you will be kept separately from the rest of the information and answers you provide.

With your permission, we would like to be able to contact the person or agency that referred you to the study and let them know if you were enrolled in the study or not and to which of the study groups. We will not sure any of the information you have provided to us with them – only if you are in the study or not and, if you are, which group you are in. We would like to do this in order to make sure that you continue to receive all of the existing community services that you need.

If now or in the future you have any questions related to this screening interview or the research study you may contact Kate Mason, Research Coordinator, Centre for Research on Inner City Health, St. Michael's Hospital at 416-864-6060 x 6513.

Verbal Consent Check List

→ Has the study screening process been explained to your satisfaction?

→ Do you understand that you have the right to choose to not be screened for this study?

→ Do you understand the potential risks, harms and discomforts of participating in this screening?

→ Do you understand the benefits of participating in this screening?

→ Do you understand that you have not waived any of your legal rights nor released the investigators, sponsors, or involved institutions from their legal and professional duties?

→ Do you understand that you may ask now or in the future any questions about the screening process or the study?

→ Do you feel assured that information relating to you and your health care will be kept confidential and that no information will be released or printed that would disclose your personal identity without my permission unless required by law?

→ Have you had enough time consider this information?

→ Do you have any additional questions right now?

→ Have your questions about the study been adequately answered?

→ Would you like to participate in this study?

Begin screening process when all of the above questions have been answered "Yes" *[except for 'Do you have any additional questions right now?' → proceed to next question only after participant has no more questions]*

If you do not give us permission to keep the information you provide in this screening interview, please initial here and I will then proceed.

Initials________

If you do not give us permission to contact the person or agency that referred you to the study, please initial here and I will then proceed.

Initials________

With your permission, I will proceed.

Appendix Q: Question 23A - Additional persons with access to personal health information.

Access to the data will be limited to authorized users only, using a multi-level, permissions protocol that will specify roles and types of data access permitted, using the need to know principle.

In addition to the Investigators listed in Question 23B, the remaining Co-Investigators (listed in 3B) will have access to health information data for the purposes of data analysis but will not have access to personal identifiers. Research Coordinators and Assistants employed by the Centre for Research on Inner City Health, with specific roles such as participant follow-up or chart review will have access to the data for such specified purposes. The St. Michael's Hospital Research Ethics Board will also have access for monitoring purposes.

Geoff Couldrey, BAH, IS Dip., (Information Systems Manager, Mental Health Commission of Canada) has responsibility for access management of users of the information system at the national and site-specific levels. He is responsible for overseeing all security related matters nationally and at each site, including adherence to security policies and secure data system storage and back-up. It is not necessary for Mr. Couldrey to view personal health data per se for any reason, however, while in the performance of his duties he will have access to such data.

At the national level, Dr. Carol Adair, MSc, PhD, (National Research Team Lead, Measurement; Mental Health Commission of Canada/University of Calgary), who is responsible for the multi-site study design and data collection, will have access to the personal health information data of the study in her oversight of the study operations at the national level including day-to-day activities, monitoring recruitment, participant duplication across sites, and identifying site issues that might impact the study overall. Dr. Jayne Barker (Director of Policy and Research, Mental Health Commission of Canada) and Dr. David Striener (National Data Analysis Lead, Mental Health Commission of Canada) will also have access to the full data set at the national level for data analysis purposes but will not access identifiable data.

The technical staff at HealthDiary (the vendor providing the study's information system and data storage) do not access records but require access to servers and applications related to our study and, as such, could potentially access personal health data. They include:

- Jerry Legierski, President, HealthDiary Inc.
- Steve Hancock, Network Services Manager, HealthDiary Inc/BIT Incorporated
- Gerald Karmadi, Development Manager, HealthDiary Inc.
- Moe Islam, Senior Developer, HealthDiary Inc.

Appendix R: Additional Consent Measures

This form will be filled out as the participants go through the consent process to ensure that each section is clearly understood.

Research Demonstration Project in Mental Health and Homelessness Study
RESEARCH CONSENT QUESTIONNAIRE

clear	re-explained	
**VOLUNTARY**		
☐	☐	Do you have to participate in this research study?
☐	☐	Once you have signed the consent form, do you have to stay in the study until the very end?
☐	☐	If you decide not to enter the research study will it effect your ability to access any of the services you normally would?
**RISKS AND BENEFITS**		
☐	☐	What are the benefits of being in the study?
☐	☐	What are the risks of being in the study?
**CONFIDENTIALITY**		
☐	☐	Will the information you provide to us be kept absolutely confidential?
**TIME REQUIRED**		
☐	☐	How long will you be enrolled in the study?
☐	☐	How many interviews will you have to do? How long will they take?
**REIMBURSEMENT**		
☐	☐	Will you be paid for entering the study?
**QUESTIONS**		
☐	☐	If you have questions about the study, who should you ask?
☐	☐	If you have questions about being involved in a research study in general, who should you call?

Appendix R: Additional Consent Measures

This form will be reviewed with each participant prior to signing the consent form. A copy will be left with each participant.

Research Demonstration Project in Mental Health and Homelessness Study

WHAT WOULD YOU BE DOING IF YOU DECIDE TO PARTICIPATE IN THIS STUDY?

EVERYONE WHO ENROLLS IN THE STUDY:

1. Doing a survey interview every 3 months for the next 2 years about your health, housing situation, use of health care and other social services, and overall well-being.

2. Calling the Research Office once a month for the next 2 years to provide a quick update about your whereabouts.

3. Allowing researchers to contact the people, service providers, or provincial agencies that you have given us permission to contact if we can not get in touch with you and need to follow up with you do another research interview.

ONLY IF YOU WISH:

4. Allowing researchers to access information about your health care use, shelter use, justice system contacts, social assistance benefits, and prescription drug use from provincial and city databases.

5. Participating in other kinds of research interviews. For example, telling researchers about important life moments in your own words.

ONLY IF YOU ARE RANDOMLY ASSIGNED TO RECEIVE HOUSING AND SUPPORT SERVICES:

6. Meeting with your case manager or a member of your Assertive Community Treatment team at least once a week.

IT'S YOUR CHOICE!

- You do not have to participate in this study if you don't want to.
- You will still be able to access all of the services you normally would whether you are in the study or not.
- Your information will be kept strictly confidential and very secure. Only the researchers will see it.
- You can stop being in the study any time you wish.

If you have any questions about the study at any time, you can contact Kate Mason, Research Coordinator at 416-864-6060 ext 6513.

Procedures for Ensuring Participants' Immediate Health and Safety & Interviewer Security and Safety

Procedures for Ensuring Interviewers' Safety & Security
Procedures will be in place to ensure the security and safety of all interviewers. Whenever possible, interviews will take place at the field office where the intervention service providers will also have their offices. For interviews that take place outside of this location, interviewers are required to call the project phone line at the beginning and ending of each interview. Interviewer calls at the beginning of each interview will be made in the presence of the participant in order to maintain transparency. The Centre for Research on Inner City Health's Survey Research Unit will maintain a schedule detailing upcoming interview times, locations, and the names of interviewers assigned to each of interview. This mechanism will keep the study team aware of interviewer locations at all times. If interviewers are using their own cell phones to confirm interviews with participants, it is required that they use the Call Blocking feature on their cell phones. This call blocking feature allows you to prevent your name and number from being displayed on the current outgoing call.

Additional protocols will ensure the safety and security of project interviewers for interviews taking place outside of the field office and include the following:

Preparing & Planning the Interviews

- For participants in the intervention group, the Research Coordinator will discuss with the study service providers any particular concerns or strategies for individual participants or housing situations.
- Each interviewer will be equipped with a project cell phone unless s/he has his/her own cell phone. This phone will ONLY be used for project purposes. Interviewers should always use the project cell phone for communication with the respondents if necessary. This way, the project number can be traced but the interviewers themselves cannot be.
- If, after setting up an appointment, interviewers feel uncomfortable conducting the interview alone, they will be directed to speak to the Research Coordinator and arrangements will be made for them to be accompanied.
- Once an interview has been set up, the interviewer will be contacted by phone or email with the date, time and location of the interview.
- Interviewers should not accept any food, beverages or gifts from participants. To avoid potentially insulting the participant, interviewers are to bring their own bottle of water to drink throughout the interviewer. However, food should never be consumed during an interview.

Safety & Security While Conducting Face-to-Face Interviews: Tips for Interviewers

- Beware of dogs or other animals at the door. Do not enter the premises until the participant has removed a barking dog to another room. Placing your foot at the bottom of a screen door will often prevent a dog from lunging at a door and pushing it open.

- When entering an apartment or other multi-residential building, make note of the location of the building superintendent. Also make a note of the locations of fire exits.
- Observe how the front door lock operates, so that you could let yourself out if necessary.
- Sit so you are closer to the door than the participant.
- Always keep a cell phone next to you during an interview. You must answer your phone if St. Michael's Hospital is calling you, even if you are in the middle of the interview.
- If the interviewer feels uncomfortable or unsafe upon arrival, they are to inform the participant that they ***forgot the lap top password*** and need to reschedule the interview. If the participant states that they should continue regardless, inform them that the interview cannot be conducted without getting the laptop password. The interviewer is to then leave the location immediately and contact the Survey Research Unit. If the interviewer needs to place the call in front of the participant, tell the Research Coordinator that you ***forgot the lap top password*** and they will understand this code.
- If the interviewer feels uncomfortable anytime during the interview and feel that their safety is being compromised, they are to inform the participant that they ***forgot the paper and pencil part of the survey*** and cannot continue with the interview.
- Use extreme caution if faced with violent or intoxicated respondents. Leave immediately, fill-out an incident report and report the case to the Research Coordinator. If a respondent becomes belligerent during the course of the interview, end the interview early by stating that you ***forgot the paper survey***, do everything to avoid provoking the respondent and leave the premises. Then call the Research Coordinator and the police if appropriate.
- After you have introduced yourself and entered the premises, call the Research Coordinator to inform her/him that you have arrived. This will show the participant that others know where you are. If you feel unsafe in the street before making contact, you may want to call earlier as well.
- Participants should be paid once the interview is completed. If an interview is terminated before completion the participant should be fully compensated, especially if the participant is agitated or aggressive. If an interview is terminated in this fashion it is often best to feign interview completion rather than to disclose premature termination.
- After you have completed and left the premises, call the Research Coordinator to inform her/him that you have left the premises.
- Interview times will vary in length at different points in the study. If the Research Coordinator has not received a telephone call from you indicating the completion of the interview within thirty minutes of the expected completion time at the point you are interviewing, s/he will set in motion the emergency procedures as listed below.
- The study team will be on-call during all hours that interviewers are in the field conducting interviews.

Procedures for Ensuring Participants' Immediate Health and Safety

Because the questionnaire asks participants questions regarding their emotional state, it is possible that some participants may experience some mild negative responses, such as sadness or regret, or in some instances identify an unresolved health need. In rare cases, participants may experience emotional upset, and even less frequently, lapse into crisis (i.e. pose a threat to

themselves or others). We have a protocol for dealing with such situations and we will train all interviewers who will be interacting with study participants in this protocol.

Protocol for interviewers will be as follows:

- If interviewers identify participants as being at least mildly upset, interviewers will ask participants if they want to skip questions, take a short break, discontinue the interview or continue at a later date, or withdraw from the study.
- In cases that participants want to withdraw, interviewers will thank them for their time and provide them the honorarium. Interviewers will also provide the resource sheet, offer to explain the resources on the sheet, and offer to be present if participants want to contact the listed resources immediately.
- In cases that participants want to discontinue the interview and continue at a later date, interviewers will schedule another appointment with the participant. Interviewers will also provide the resource sheet, offer to explain the resources on the sheet, and offer to be present if participants want to contact the listed resources immediately.
- In cases that participants would like to take a short break or skip the questions, interviewers will comply with the request. Interviewers will also provide the resource sheet, will offer to explain the resources on the sheet, and offer to be present if participants want to contact the listed resources immediately.

Questions that deal with suicide and other sensitive topics will have specific procedures which will be once the core measures have been finalized. A safety/risk protocol has been established by the national research team which will give Research Assistants a back-up clinician to contact based on a specific operational decision around responses on risk items, a referral card for a contact clinician, and a number for crisis services. An example of the type of procedure that will be implemented for such questions is outlined below:

- For a question about suicidal ideation, if the respondent replies with a response between 'a little of the time' to 'all of the time' the interviewer will ask if the respondent is feeling that way right now.
- If the respondent states that they are not, continue with the interview.
- If the respondent states that they are currently feeling that way, interviewers will ask the respondent if they would like them to call either a clinician, crisis intervention centre or 911 for assistance. If the respondent states they would like the interviewer to call for assistance, he/she will stay with them until assistance arrives and will contact the Research Coordinator to inform them of the situation. The Research Coordinator will travel to the respective interviewer's location to assist in the situation if needed.
- If the respondent states that they do not want anyone to be called, interviewers will ask them if they would like to continue the interview and do so accordingly. Participants will be referred to the mental health services in the brochure when providing the honorarium to the participant.
- If at any point the interviewer feels that their safety is compromised they are to leave the location immediately, using any exit strategy that they feel is necessary.

Appendix S: Safety Protocol

Procedures for Ensuring Participants' Confidentiality

Interviews will only be conducted in a private setting. Before beginning an interview, interviewers must remind participants that the interview must be conducted in a private setting. The participant will be free to reschedule (or relocate) the interview to a time that may be more private, convenient and safe. Interviewers will avoid asking participants to answer questions in front of others in order to maintain confidentiality. If participants have visitors, the interviewers will ask participants if it would be preferable for the interviewer to return. Even if the participant stays that they are comfortable with another person being around, the interviewer must indicate that some of the questions may be of a personal nature and that privacy is necessary for the completion of the interview. If the participant objects, ask to reschedule (or relocate) the interview to ensure privacy.

Circumstances Involving the Identification of Intimate Partner Violence (IPV)

The act of revealing details of abuse to someone outside the family can expose participants to further risk. For these reasons, it is critical to maintain the confidentiality of information collected during the interview. It is important that interviewers stop the interview immediately when a third party enters the room, the interviewer is to remind the respondent that they cannot continue the interview until they are alone. Once the third party has exited the room, the interviewer is to ask the respondent if they feel comfortable continuing the section and give them the option to fill out a paper copy of that section. This is given as an option in case the participant fears that their partner may over hear them answering the questions.

If a participant becomes distressed or asks the interviewer for help in overcoming their situation, the interviewer should consult the list of resources on IPV and identify one or more organizations where victims can seek shelter, counselling or any other kind of help which is closest to the neighbourhood in which the participant resides. If the participant asks for assistance in contacting resources, the interviewer may stay with the participant while they make the appropriate calls. Please note, that if the participant asks the interviewer to call the police for them, the interviewer can fulfill this request. If the participant does not ask for help, but intimate partner violence is suspected, the interviewer is to ensure that they explain the relevant resources in the brochure at the end of the survey with the participant. This is to be done very casually and not in a way that leads the participant to believe the interviewer thinks they need to use the resources. In order to do this, when you are explaining the brochure at the end of the interview, make sure you point relevant resources and inform the participant that they "might find these resources helpful".

Health care professionals/providers and staff affiliated with St. Michael's Hospital or the University of Toronto are not obligated both legally and professionally to report suspected or verified partner violence. Accordingly, interviewers will not give specific information on sources of help to a participant unless the participant asks for assistance.

Circumstances Involving Verified or Suspected Child Abuse

Any suspected incidences of child abuse must be documented in an incident report and passed on to the Research Coordinator immediately. If a participant indicates that a child they know is

being abused by them, a partner/spouse, or any other party, or if you come into contact with a child that you assume is suffering from abuse interviewers will be instructed to pass this information on to the Research Coordinator right away.

Circumstances Involving Verified or Suspected Elder Abuse
If a participant indicates that an elderly person they know is being abused by them, a partner/spouse, or any other party, interviewers will be instructed to document and pass this information on to the Research Coordinator right away.

Documenting Incidents
If a problem should arise during an interview please document it on the following form and return it to the Research Coordinator as soon as possible. You will be given one of these forms with your interview package for each interview you conduct.

STANDARD EMERGENCY PROTOCOL

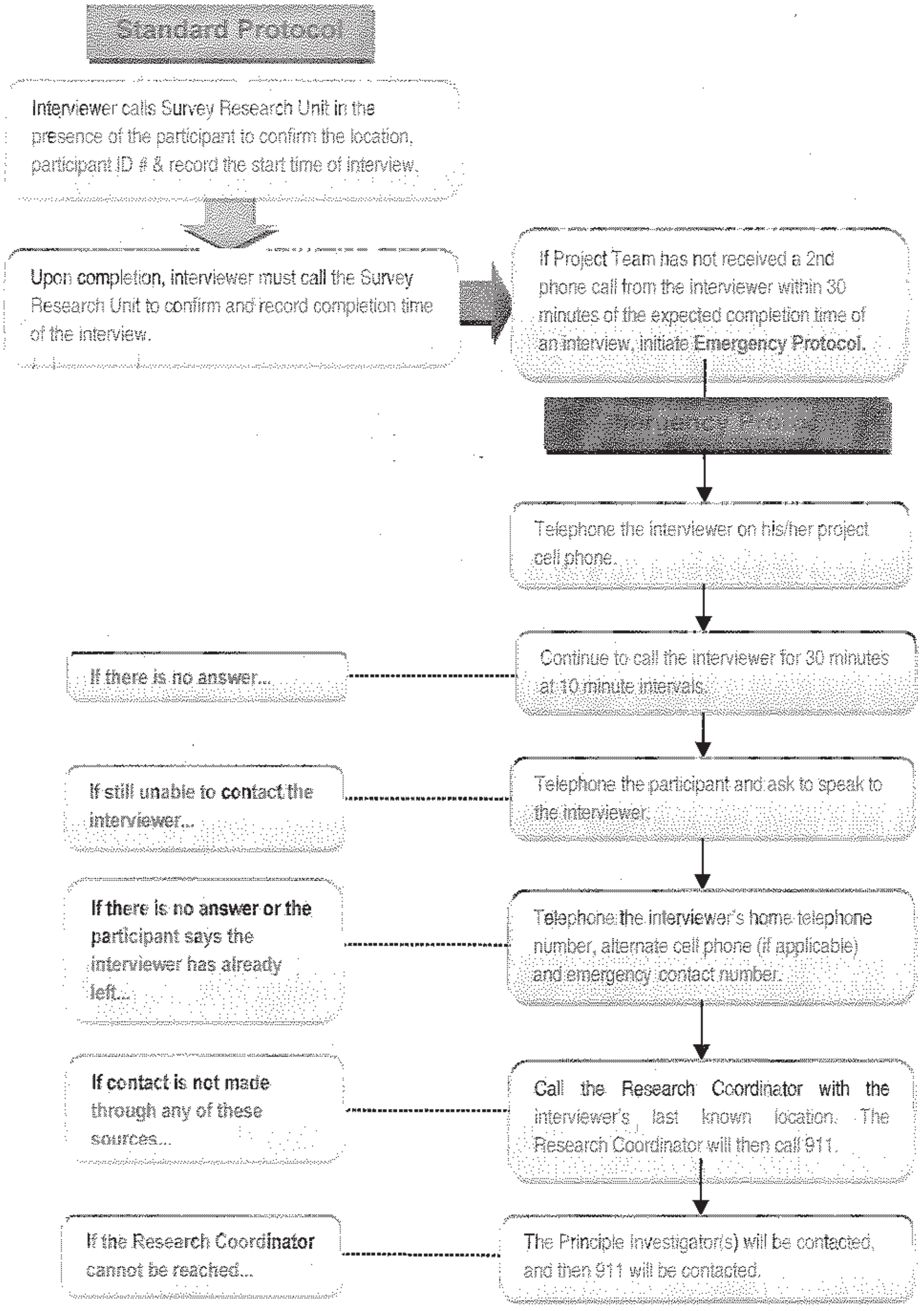

Incident Report

**General information**	
Interviewer Name:	
Participant ID:	
**Description of the event**	
Date of Event (MM/DD/YYYY)	Time of Event:
Date Reported	Time Reported:
Location of Event:	
What happened? (Description of the event and how it occurred)	
Were you injured? (Description of injury, including parts of the body)	
What factors contributed to the event?	
How could the event have been avoided?	

Appendix T: Budget

"At Home" Research Demonstration Project on Homelessness and Mental Health
Toronto Site Research Budget

	2009 - 2010	2010 - 2011	2011 - 2012	2012 - 2013	Total
Research Staff	493,250	701,958	689,657	635,485	**2,520,350**
Operating Costs	188,450	231,155	233,640	178,582	**831,827**
General & Administrative	68,550	56,303	53,851	56,955	**235,659**
**TOTAL**	**750,250**	**989,416**	**977,148**	**871,022**	**3,587,836**

Research Staff: includes salaries for research coordinators, research assistants, and survey interviewers
Operating Costs: includes participant honoraria, travel costs for interviewers, translation costs, 1-800 number, data entry
General & Administrative: Includes meetings, travel for research meetings, materials, office supplies, IT supplies, services, communication, equipment <$

;10,000

AT HOME STUDY
COMMUNITY RESOURCES INFORMATION GUIDE

This sheet contains information about agencies and programs you may want to contact about your health or well-being. There are more services and programs available to you than those listed below. If you don't see what you're looking for please contact the City of Toronto's Assessment & Referral Centre at 1-877-338-3398.

Quick Reference

211 Community Information
Dial 2-1-1 for information on community, social, health and government services in Toronto or visit www.211toronto.ca.

Shelter & Housing

Emergency Shelter:
Assessment and Referral Centre: 67 Adelaide Street East. Open from 8:30 pm to 7:30 am. Safe place for people who are looking for supports or shelter.
Referral line operates 24 hours/day.

- Single adults, youth, couples: 416-339-4766 / 1-877-338-3398
- Central Family Intake: 416-397-5637

Streets to Homes:
City program to assist people to find housing.
Call 416-392-0900 or Drop-In to the office at 112 Elizabeth St. (west of Bay, off Dundas) from Monday to Friday: 9am to 3pm

Street Help Line:
Referrals for people who are homeless including counselling, health care, and access to long-term housing. Free from any pay phone. Tel: 866 - 392 - 3777

Mental Health

Crisis Lines:

- **Distress Centres of Toronto** – 24 hours
- Telephone response to persons in need of immediate emotional support, crisis intervention or suicide prevention
- 416-408-HELP

- **Gerstein Crises Centre**
- 416-929-5200

- **Anishnawbe Health**
- 416-891-8606

- **Centre for Addiction & Mental Health**
- 416-535-8501 x 6885

Community Mental Health Agencies:

Toronto North Support Services
Case management and outreach services.
132 Railside Road, Unit 2 / Tel. 416-499-5969

COTA Health
Case management, supportive housing, outreach and other services.
Tel. 416-785-2779 / 1-888-785-2779

Across Boundaries
Offers a range of support and services to people of colour.
51 Clarkson Avenue / Tel. 416-787- 3007

Parkdale Activity Recreation Centre (PARC)
Drop-in and support services.
1499 Queen Street West

Street Health
Case management and mental health support.
338 Dundas Street East / Tel. 416-921-8668

Sound Times
Member-run consumer/survivor support agency.
280 Parliament Street / Tel: 416-979-1700

Community Care Resources
Access point for health and personal support
Tel: 1-866-243-0061

Anishnawbe Health
Mental health and traditional healing: 416-920-2606 / 179 Gerrard Street East
Mental health and addictions services: 416-657-0379 / 22 Vaughan Road

Health Services

Shelters & Drop-Ins with Psychiatrists & Doctors:

- No health card needed
- For more location, please visit: www.icha-toronto.ca

- 416 Drop-In (416 Dundas St E) – Psychiatrist on Mondays from 1 to 4 pm- Family Doctors on Tuesday and Thursdays
- Agincourt Community Services (4155 Sheppard Ave E #100) – Psychiatrist on Thursday from 10 am to 1 pm
- Canadian Centre for Victims of Torture (194 Jarvis St 2nd Floor) – Psychiatrist on Wednesday from 9:30 am to 4:30 pm.
- Evangel Hall (552 Adelaide St W) – Family doctor on Tuesday & Friday from 9:30 am to 12:30 pm.

- Corner Drop-In (260 August Ave) – Psychiatrist on Tuesday from 9:30 am to 1:30 pm. Family Doctor on Tuesday mornings.
- Seaton House – Family Doctors & Psychiatrists available weekdays.
- Women's Residence (674 Dundas St W) – Psychiatrist on Mondays from 2 to 5 pm, Family Doctor on Friday mornings.
- Evergreen Youth Centre (381 Yonge St) – Psychiatrist on Thursdays from 1 to 5 pm.
- Newtonbrook Twaiwanese Centre (4 Wellesley Place) – Family Doctor on Tuesday from 1 to 5 pm. Psychiatrist once a month on Thursday afternoon.

Sherbourne Health Centre Unscheduled Care Clinics:
No appointment or ID required. Nurses, Doctors and Resource Workers available.
333 Sherbourne St. just north of Gerrard / Tel. 416 324 4160
Tuesdays → 12:30 a.m. – 4:00 p.m.
Fridays → 9:00 a.m. – 12:00 p.m.

Regent Park Community Health Centre:
Urgent care hours every afternoon
WOW Clinic (no appointment needed) - Wednesdays 10:30 am -12:30 pm
Tel. 416-364-2261

Access Alliance Multicultural Health and Community Services
Programs and Services for immigrants and refugees living in Toronto.
Main Office: 340 College St # 500 / Tel: 416-324-8677

East Office: 3040 Danforth Ave., #6 / Tel: 416-693-8677
West Office: 881 Jane St., #200B / Tel: 416-760-8677

Street Health Nursing Clinics:
No appointment or ID required.
Tuesdays & Thursdays: 12 noon to 2 pm - All Saints Church (Dundas/Sherbourne)

Anishnawbe Health Toronto (Aboriginal clients):
Primary health care: 416-360-0486 / 225 Queen Street East

St. Michael's Family Practice Units (need an OHIP card):

410 Sherbourne St.	Tel: 416-867-3728 or 416-864-3096
61 Queen St. East	Tel: 416-867-7428 or 416-867-7426
140 Esplanade	Tel: 416-864-6000

Telehealth Ontario:
Confidential telephone health advice or general health information from a Registered Nurse
Toll Free: 1-866-797-0000. 24 hours a day, 7 days a week.

MDOT - Mobile Multi-Disciplinary Outreach Team:
Assessment, consultation and access to medical treatment.
132 Railside Road #2, North York / Tel: 416-646-6649

Lice & Scabies:
Harrison Baths – 15 Stephanie Street (Queen St West/McCaul St) / 416-392-7984
Nurse and personal support work to treat lice and scabies. Laundry. Medical referrals.
Tuesdays & Thursdays - 12 noon to 3 pm

Identification Replacement

Street Health workers will help you replace or get **identification documents** like birth certificates, Ontario Health Cards, Social Insurance Cards, and Landed Immigration Documents. No charge. Must be homeless. Street Health will also receive and store a person's mail.

Call Linda at ID Safe: 416-925-0043 or visit 338 Dundas Street East (just east of Sherbourne).

Dental Care

Faculty of Dentistry, University of Toronto
101 Elm Street (Sept-June/adults)
416-979-4927

Regent Park Dental Clinic
465 Dundas St. East
416-364-7019

Queen West Community Health Centre
168 Bathurst Street
416-703-8481

Toronto Public Health Dental Services
(416) 392-0907 – staff will direct you to a local clinic that fits your needs

Women's Health

Women's Health in Women's Hands
Community Health Centre that provides healthcare to black women and women of colour.

Assaulted Women's Helpline – 24 hours/7days
416-863-0511

Toronto Rape Crisis Centre
416-597-8808

Sexual Health

Bay Centre for Birth Control
416-351-3700

City of Toronto's **AIDS and Sexual Health Hotline:** 416-392-2437

Hassle Free Clinic
Provides free medical and counseling services in areas of sexual health. Located at 66 Gerrard Street East (at Church). Tel: 416-922-0566 (Women's Clinic) or 416-922-0603 (Men's Clinic).

- No health card required. Separate hours for men and women. Trans men/women welcome at both. Anonymous HIV testing

Alcohol & Drug Use

Detox Centres:
Residential, day and community services must all be accessed through 1-866-366-9513

Metro Addiction Assessment Referral Service
Information about treatment services and in-person assessment
416-599-1448 / 175 College Street

The Works: Toronto Public Health harm reduction program
Located at: 277 Victoria Street (near Dundas Square), from 10 - 5 Monday to Friday.
Telephone: 416 392-0520 (from 8:30 a.m. to 12:00 a.m. Monday to Friday)

Services provided include:

- Distribution of needles and other safer drug use materials
- Testing for Hepatitis A & B, C, HIV, and Syphilis
- Immunization against Hepatitis A, B, Influenza, Pneumococcal, and Tetanus & Diphtheria (Td)
- Methadone Maintenance
- Information and education about safer drug use and safer sexual practices
- Client Support, referrals and assistance in accessing services

Agencies that distribute safer crack kits:
Street Health (Dundas/Sherbourne)
Parkdale Community Health Centre (Queen/Dufferin)
Queen West Community Health Centre (Queen/Bathurst)
Regent Park Community Health Centre (Dundas/Parliament)
Sherbourne Health Centre – Health Bus
South Riverdale Community Health Centre (Queen/Carlaw)
The Works (Dundas/Victoria)

Income & Employment

Ontario Works (OW)
Toronto Employment and Social Services Information Unit
Tel: 416-392-2956

Ontario Disability Support Program (ODSP)
Ontario Ministry of Community and Social Services
Information Line →1-800-808-2268
Downtown Office – 385 Yonge St, 2nd Floor

Sex Professionals of Canada (SPOC)
SPOC is a political and social group that provides resources and referrals for sex workers.
For more information contact Wendy at 416-731-1389

Appendix V

Figure 1: Randomization Flow Chart

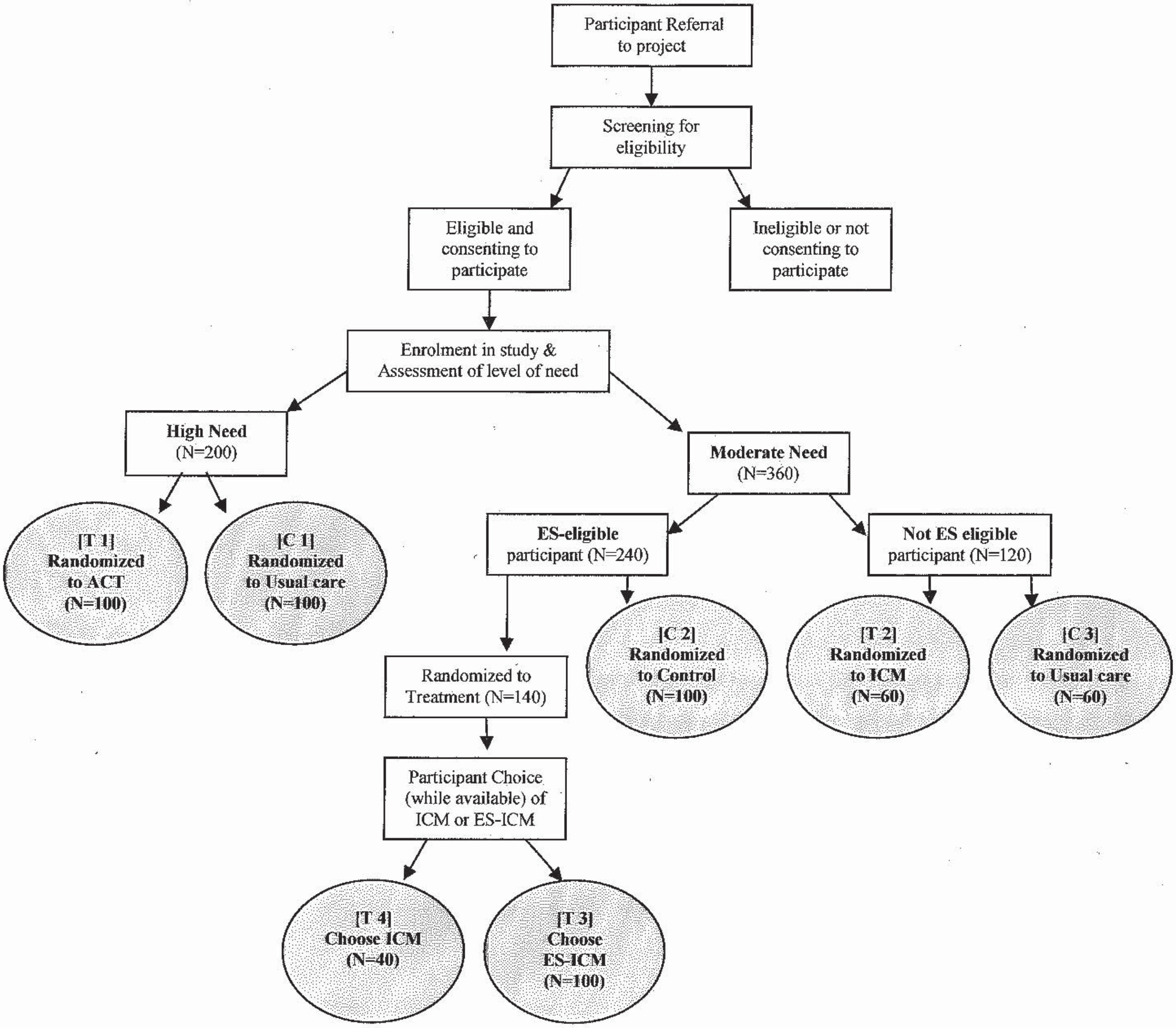

(N=): number of participants

ES: Ethno-Specific

ICM: Intensive Case Management

ACT: Assertive Community Treatment

Questions:

1) Among High Needs: ACT v. ICM
2) Moderate Needs: ICM vs UC → T2 <> C3; T4 <> C2.
3) Moderate Needs: ES-ICM vs. UC → only for those ES eligible T3 = C2
